# Electrochemical Dehydrogenative Acetalization Protection of Alcohols with Tetrahydrofuran

**DOI:** 10.1002/celc.202101155

**Published:** 2021-10-21

**Authors:** Raolin Huang, Congjun Yu, Frederic W. Patureau

**Affiliations:** ^1^ Institute of Organic Chemistry RWTH Aachen University Landoltweg 1 52074 Aachen

**Keywords:** cross dehydrogenative coupling, dehydrogenative C−O bond formation, electro-oxidative method, oxidative acetalization, alcohols

## Abstract

A mild, facile, and environmentally friendly electrochemical protocol for the C(sp^3^)−H/O−H cross dehydrogenative coupling between various alcohols and tetrahydrofuran with H_2_ evolution is herein reported. This synthetic strategy does not require external oxidants nor catalysts. The broad functional group compatibility includes hydroxyl, halogens, olefins as well as an alkyne. Initial mechanistic investigations were conducted. The method provides a green and efficient hydroxyl group protection.

## Graphical abstract

**Electro‐protection**: A mild, facile, and environmentally friendly electrochemical protocol for the C(sp^3^)−H/O−H cross dehydrogenative coupling between alcohols and tetrahydrofuran with H_2_ evolution is herein reported.

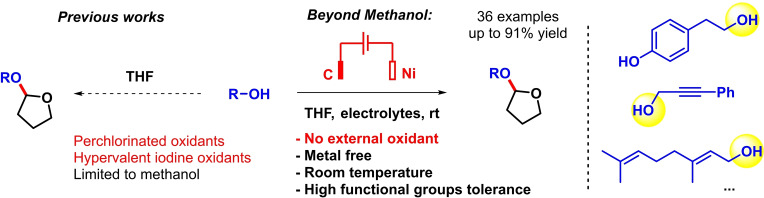

The conversion of alcohols into acetals represents a very effective functional group protection, which is easily removed under acidic conditions.[^1^, ^2^] In this field, tetrahydrofuranylation is a privileged protection strategy because of optimal lability/stability properties, and constitutes an interesting alternative to the usual but reputed less labile THP protecting group.^[3]^ Moroever, acetals are widely found in pharmaceuticals and fragrances and are also common synthetic intermediates.[^4^, ^5^, ^6^, ^7^, ^8^] Yet, the construction of this functional group usually requires inconvenient, sometimes highly toxic additives, especially from the inexpensive tetrahydrofuran (THF) commodity. In this context, dehydrogenative tetrahydrofuranylation reactions are usually carried out at high temperature in the presence of catalytic amounts of transition metals (Fe, Cu salts) in combination with chemical oxidants such as DTBP.[^9^, ^10^] Metal‐free procedures have also been developed.[^11^, ^12^, ^13^, ^14^] Some early protocols utilized CCl_4_ or similar perhaloalkanes to promote the reaction.[^15^, ^16^] However, their toxicity makes such methods unattractive. Hypervalent iodine compounds can be utilized as well as terminal oxidants in this reaction with high temperatures or microwaves.^[17]^ Alternatively, photocatalytic methods have also been developed.[^18^, ^19^, ^20^] A representative selection is shown in Scheme 1a–c. Meanwhile, the field of electro‐oxidative synthetic methods has considerably expanded over the last few years, due notably to significant atom economy advantages over more traditional (chemical) methods.[^21^, ^22^, ^23^, ^24^, ^25^] Nevertheless, in spite of early seminal works demonstrating anodic oxidative C−O bond formation, especially by Shono with methanol applied as a solvent, relatively few anodic cross‐dehydrogenative C−O bond forming methods have been developed for organic synthesis (Scheme 1d).[^26^, ^27^, ^28^, ^29^, ^30^] We report herein a mild, facile and environmentally friendly electrochemical protocol for the O−H/C−H cross‐coupling between various functionalized alcohols and tetrahydrofuran with H_2_ evolution in an undivided cell under constant current conditions (Scheme 1e).

**Scheme 1:**
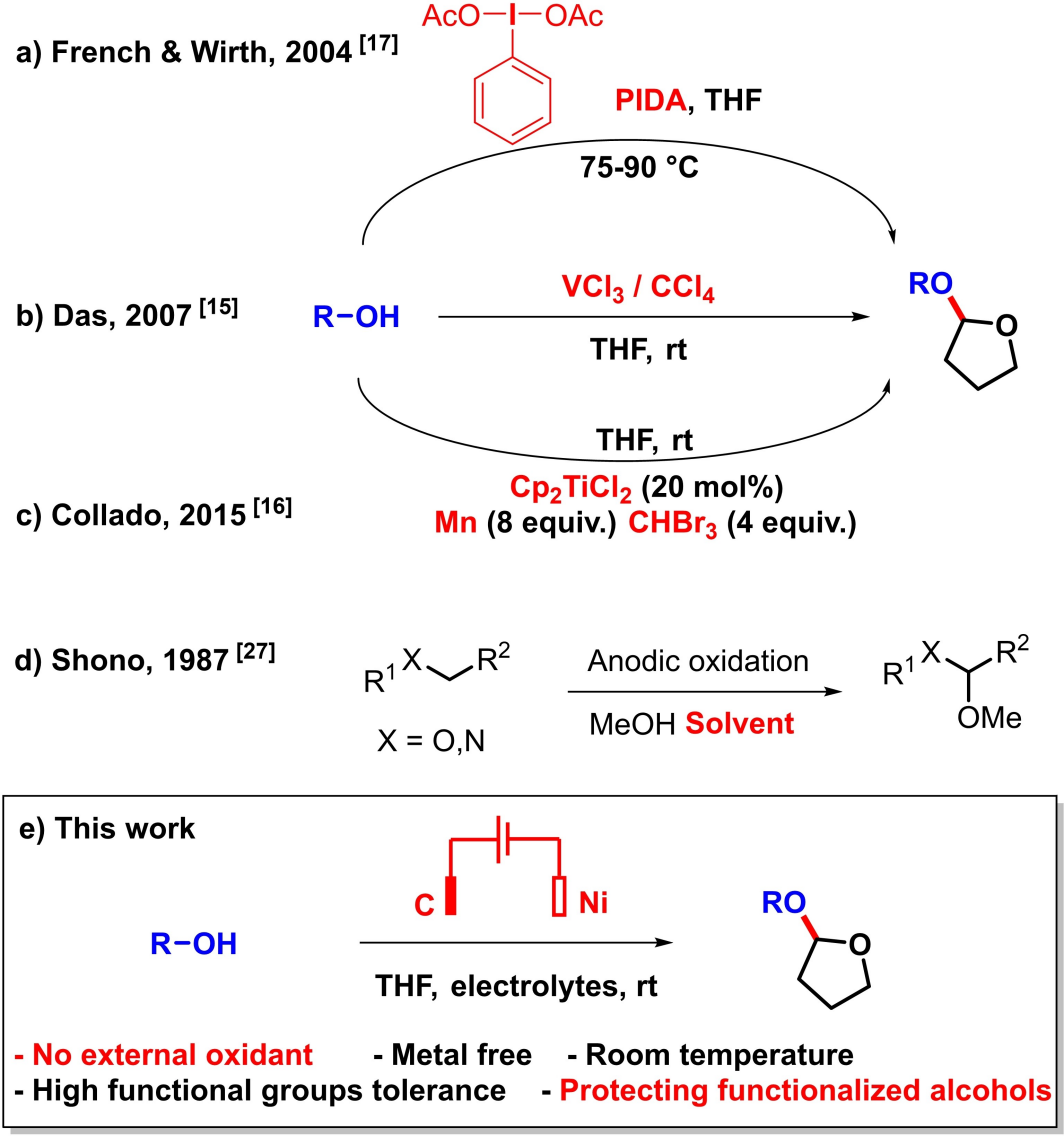
Selected cross‐dehydrogenative C−O bond formation between alcohols and tetrahydrofuran.

The electro‐oxidative method was first optimized for the dehydrogenative acetalization of phenyl ethanol **1 aa** and tetrahydrofuran (product **2 aa**, Table 1). The reaction was carried out in an undivided cell equipped with a graphite anode and a nickel cathode,^[31]^ while a constant current was utilized. An isolated yield of 62 % was initially obtained when the reaction was performed at room temperature in 5 mL of THF with 1 equivalent of nBu_4_NBF_4_ as the electrolyte (entry 1, Table 1, see the SI for electrolyte screening). The product was not detected without current (entry 2). When 50 mol% of acetic acid was added to the electrolysis, the acetal cross dehydrogenative coupling product was obtained in 76 % isolated yield (entry 3). Reducing the current to 8 mA and the use of ethyl acetate as a co‐solvent reduced the isolated yield to 48 % (entry 4). However, increasing the current to 15 mA caused an esterification byproduct to occur. Moreover, replacement of ethyl acetate with cumene (entry 5), DMSO (entry 6), DMF (entry 7) or toluene (entry 8) completely shut down the dehydrogenative acetalization reaction. In addition, other electrode materials like Ag (entry 9), Zn (entry 10) and Mg (entry 11) performed poorly as the cathode. Al (entry 12), Pt (entry 13) and stainless steel (entry 14) cathodes resulted in little effect in the reaction. The reaction benefits from both the addition of acid and avoiding cathode materials with high hydrogen overpotentials. This suggests that an efficient hydrogen evolution reaction is important for cell performance. Reducing the current to 13 mA (entry 15) or increasing the current to 20 mA (entry 16), effected only a slight reduction in yield.

**Table 1:** Screening of reactions conditions.^[a]^

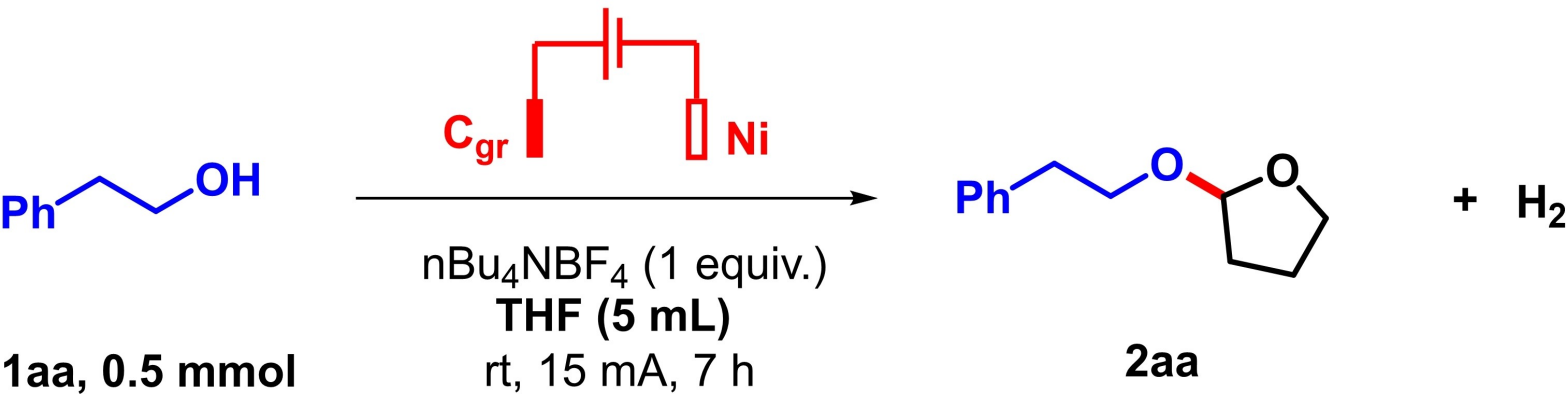		
Entry	Variation from the optimal conditions	Isolated yield [%]^[a]^
1	None.	62
2	No current.	0
**3**	**AcOH (0.5 equiv.)**	**76**
4	Ethyl acetate as co‐solvent ^[b]^	48
5	Cumene as co‐solvent ^[b]^	0
6	DMSO as co‐solvent ^[b]^	0
7	DMF as co‐solvent ^[b]^	0
8	Toluene as co‐solvent ^[b]^	0
9	Ag‐plate as cathode	38
10	Zn‐plate as cathode	45
11	Mg‐plate as cathode	54
12	Al‐plate as cathode	60
13	Pt‐plate as cathode	63
14	Stainless steel as cathode	58
15	13 mA	57
16	20 mA	57

[a] Reaction conditions: undivided cell, **1 a** (0.5 mmol), THF (5 mL), nBu_4_NBF_4_ (0.5 mmol), rt, 15 mA, 7 h. [b] Current at 8 mA, THF (2 mL), co‐solvent (3 mL).

With the optimal conditions in hand, the substrate scope was explored for the electrochemical dehydrogenative acetalization of alcohols with THF. The results are showed in Scheme 2. Alcohols reacted smoothly to afford moderate to excellent yields of the corresponding acetal products (**2 aa**–**2 bj**, 37 to 91 % isolated yields, 36 examples). It was found that a variety of different functional groups could be tolerated in the alcohol's scaffold. We first tested the alcohols with aryl groups (**2 aa**–**2 ax**, **2 az**, **2 bb**, **2 bc**). Therein, a broad variety of functional groups were very well tolerated (methyl, methoxy, F, Cl. Br, CF_3_, dimethylamine). Impressively, even an unprotected phenol was well tolerated (**2 ac**, 86 %). In the latter example, acetalization at the aliphatic hydroxyl group, as opposed to the phenolic hydroxyl group, was confirmed with 2D HSQC and HMBC NMR experiments (see SI). The reason why such an electro‐oxidation sensitive functional group such as a phenol would be tolerated is unclear. This result was moreover found to be in line with the relatively poor performance of 4‐tertbutylphenol as a substrate, which only gave a trace (<10 %) of the corresponding acetal product under standard conditions, together with some unreacted starting material.

**Scheme 2:**
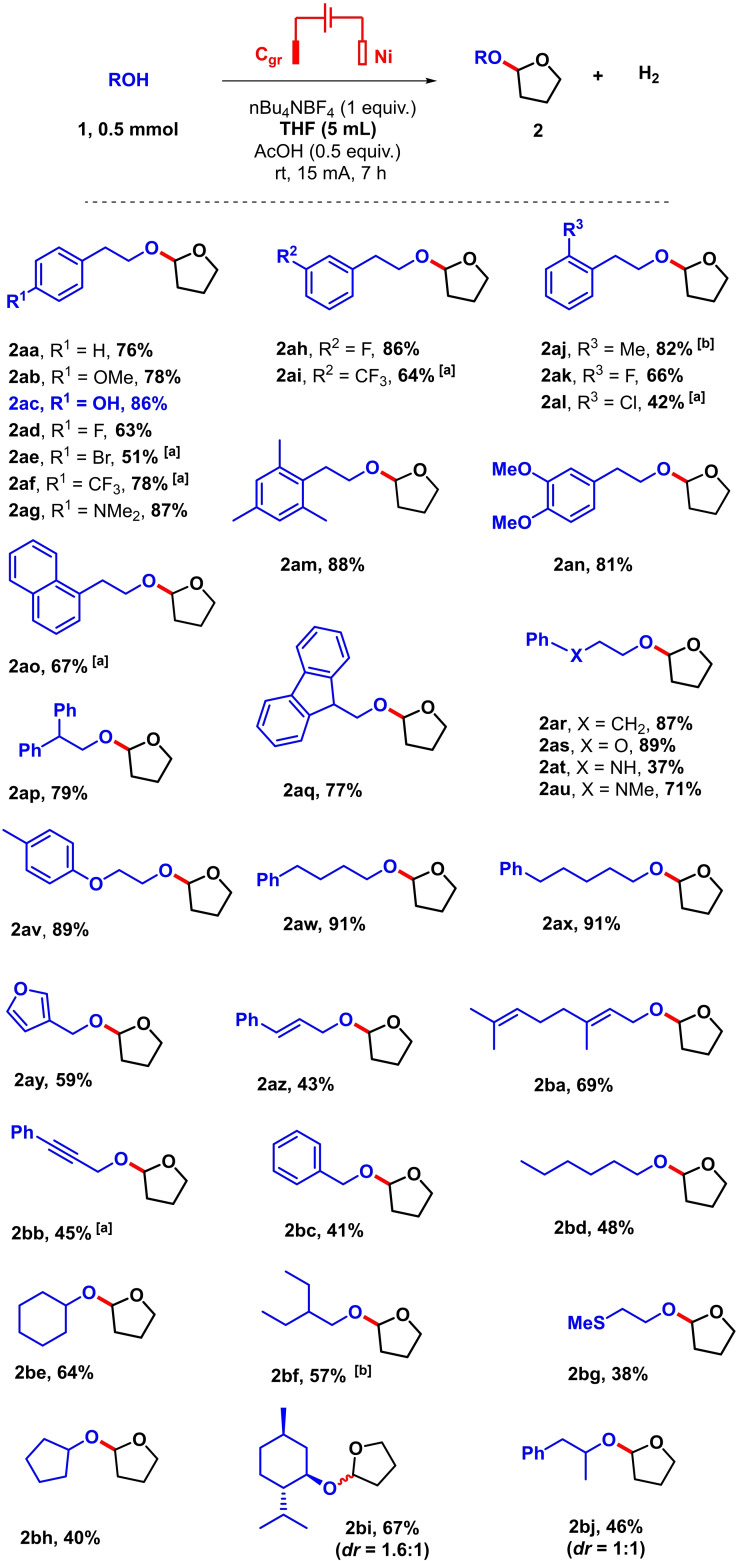
Substrate scope, isolated yields. Reaction conditions: undivided cell, (anode: graphite: 52*8*2 mm, of which 20*8*2 mm immerged, cathode: nickel: 52*8*2 mm, of which 20*8*2 mm immerged), alcohol (0.5 mmol), THF (5 mL), nBu_4_NBF_4_ (0.5 mmol), AcOH (0.25 mmol), rt, 15 mA, 7 h. [a] current was 8 mA. [b] Reaction for 6 h.

The replacement of the phenyl moiety with a naphthyl or fluorenyl moiety gave the products in 67 % and 77 % yields respectively (**2 ao** and **2 aq**). Redox labile groups, such as alkynes and alkenes were tolerated to some extent (**2 az**, **2 ba**, **2 bb**). A heterocyclic alcohol as well as thioether‐containing alcohol were likewise tolerated (**2 ay**, **2 bg**), as were a series of plainly aliphatic alcohols (**2 bd**, **2 be**, **2 bf**). Interestingly, secondary alcohols were also found competent coupling partners (**2 be**, **2 bh**, **2 bi**, **2 bj**). Even sterically hindered (−)‐Menthol converted well with a 67 % isolated yield for product **2 bi**. A mild excess for one of the two possible diastereomers was moreover noted (*dr*=1.6 : 1), although its relative configuration could not be determined at this stage. Tertiary alcohols such as *tert*‐amyl alcohol led to poorly useful and complex mixtures of yet unidentified products, while perfluorinated alcohols such as HFIP (hexafluoroisopropanol) did not react at all (neither product nor byproducts were detected).

A series of key control and mechanistic experiments were thereafter performed (Scheme 3). First, a series of cyclic and non‐cyclic ethers susceptible to be engaged as solvents in these reactions were explored (Scheme 3A). To our surprise, none delivered more than 10 % of the expected corresponding acetal coupling products, indicating that the herein described method is highly specific to THF. Alternatively, some of the acetal products might not be stable under the reaction conditions. The kinetic isotope effect (KIE) was next measured between THF and THF‐d_8_ (KIE=k_H_/k_D_). A KIE of 1.5 was thus found (Scheme 3B). Finally, when alpha methyl tetrahydrofuran was engaged as the solvent of the reaction, the corresponding acetal coupling product could be obtained in encouraging 41 % isolated yield (**3 ad**, Scheme 3C). Also encouraging was the observed diastereomeric ratio of 3.1 to 1. Unfortunately, NOESY measurements did not allow to assign the relative configuration of either the major or minor isomer. A trace of a byproduct was moreover observed, which could not be isolated nor identified at this stage. In general, it should be noted that the Faradaic efficiency in this method remains modest. Indeed, for the highest yielding example of Scheme 2 (**2 ax**, 91 %), a Faradaic efficiency of only 23 % was calculated,^[32]^ indicating the probable importance of yet unidentified side reactions.

**Scheme 3:**
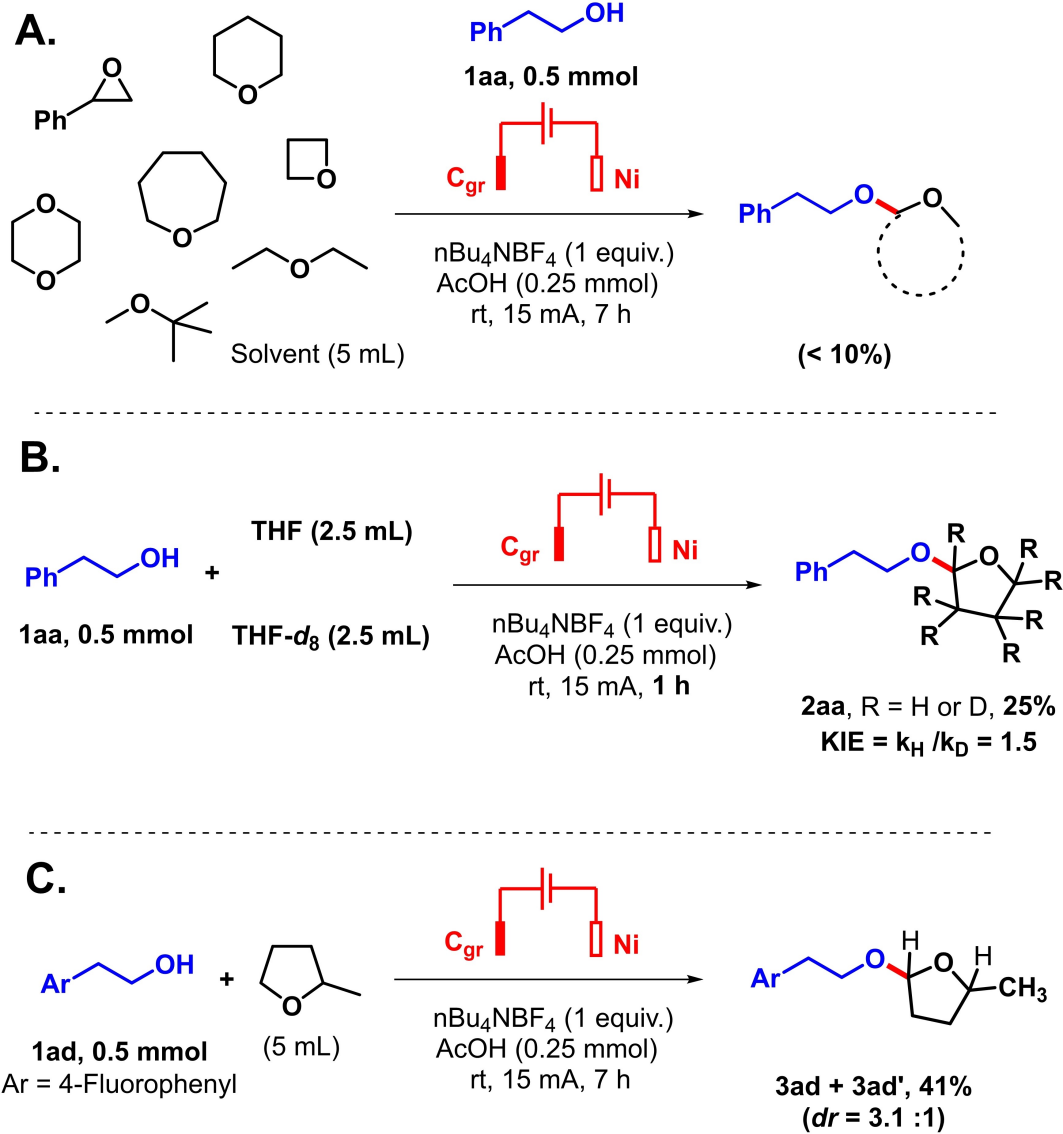
Control and mechanistic experiments.

Based on these findings, as well as from literature precedents, a mechanism is proposed in Scheme 4. First, one electron oxidation would occur at the THF solvent, followed by the release of a proton. Interception of the latter THF radical species **I** with the alkoxy radical is one possible path forward,[^33^, ^34^] which could then take place. Indeed, THF and alcohols such as methanol have similar potential windows as organic solvents for electrochemical reactions.^[35]^ However, in view of the large excess of THF solvent compared to the alcohol, further one electron oxidation of species **I** towards cationic THF species **II** seems also possible. The alcohol coupling partner would then capture cationic species **II** to form the cross dehydrogenative acetal coupling product associated to the release of another proton.

**Scheme 4:**
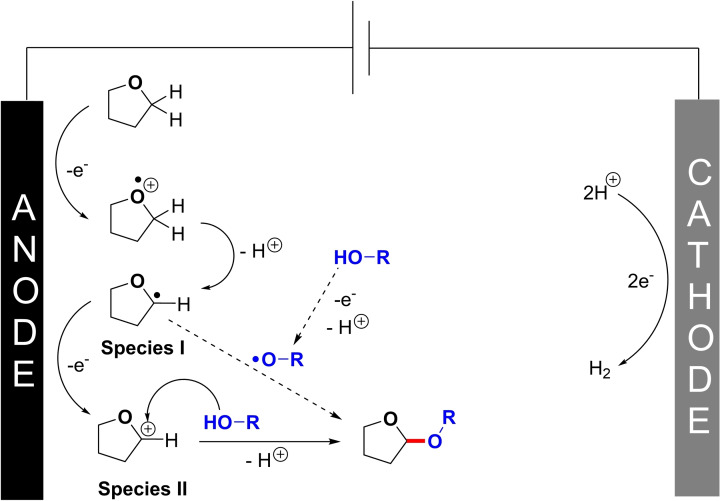
Proposed mechanism.

In summary, we developed a mild, facile, and environmentally friendly electrochemical protocol for the C−H/O−H cross‐coupling between alcohols and tetrahydrofuran with H_2_ evolution in an undivided cell under constant current conditions. The interesting functional group compatibility makes this method attractive for the sustainable electrochemical dehydrogenative protection of functionalized alcohols into acetals.

## Conflict of interest

The authors declare no conflict of interest.

## Supporting information

As a service to our authors and readers, this journal provides supporting information supplied by the authors. Such materials are peer reviewed and may be re‐organized for online delivery, but are not copy‐edited or typeset. Technical support issues arising from supporting information (other than missing files) should be addressed to the authors.

Supporting Information
