# Supplementary material for: Electrochemical Dehydrogenative Acetalization Protection of Alcohols with Tetrahydrofuran
Source: ChemElectroChem. 2021 Oct 21;8(20):3943–6. doi: 10.1002/celc.202101155 (PMC8596688; doi:10.1002/celc.202101155)
Supplement: Supplementary file 1 — Supporting Information [file CELC-8-3943-s001.pdf]

ChemElectroChem

Supporting Information

Electrochemical Dehydrogenative Acetalization Protection of Alcohols with Tetrahydrofuran

Raolin Huang, Congjun Yu, and Frederic W. Patureau*

Table of contents

1. General information S2
2. The equipment for the electro-oxidative reactions S2
3. General procedure for the electrolysis S3
4. Optimization of the reaction conditions S3
5. Product characterization S4
6. Intermolecular Competing Kinetic Isotope Effect S22
7. NMR Spectra for Products S24

1. General information

All the utilized chemicals were purchased from Sigma-Aldrich, abcr, Alfa Alfa, TCI, Fisher or chemPUR. Flash chromatography was performed on silica gel (60M, 0.04-0.063 mm) by standard technique. All the electrodes were purchased from IKA. NMR spectra was obtained an Agilent VNMRS 400 using $CDCl_3$ as solvent. Chemical shifts are given in ppm and couping constants (*J*) in Hz. The following abbreviations were used for signal coupling are: s (singlet), d (doublet), t (triplet), q (quartet) and m (multiplet) as well as combinations of them. High resolution mass spectra (HRMS) were obtained on ThermoFisher Scientific LTQ Orbitrap XL spectrometer. IR spectra were recorded on a PerkinElmer 100 FT-IR spectrometer with an UATR Diamond KRS-5 unit.

2. The equipment for the electro-oxidative reactions

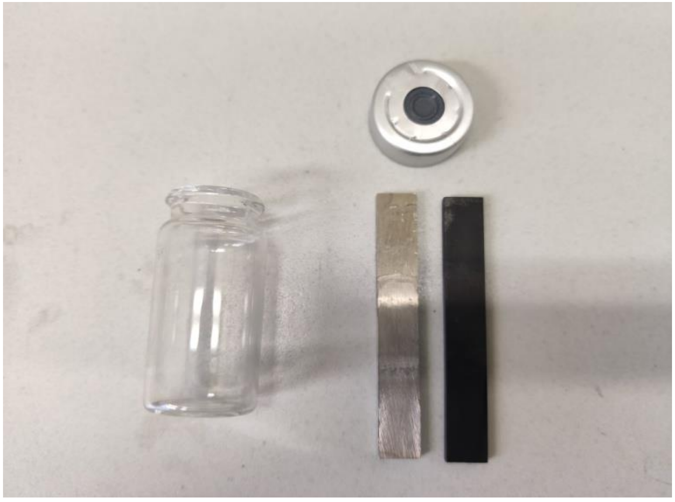

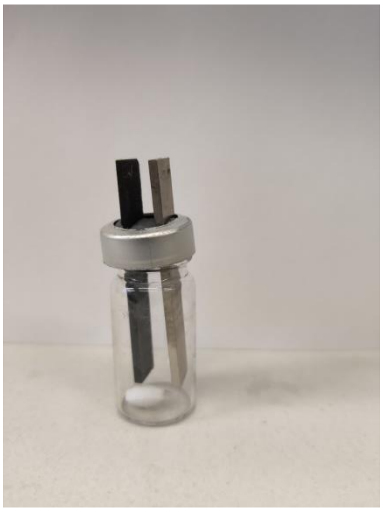

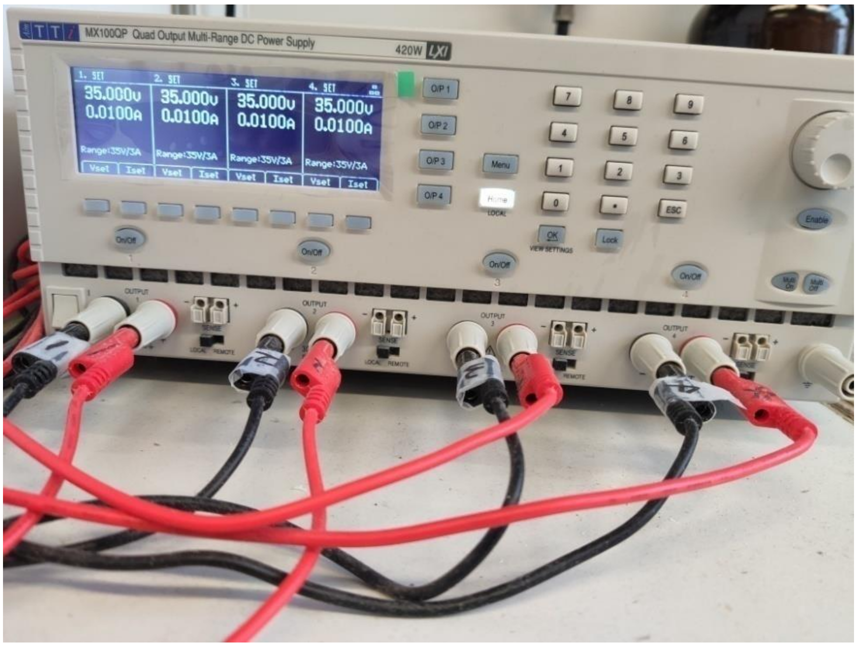

3. General procedure for the electrolysis

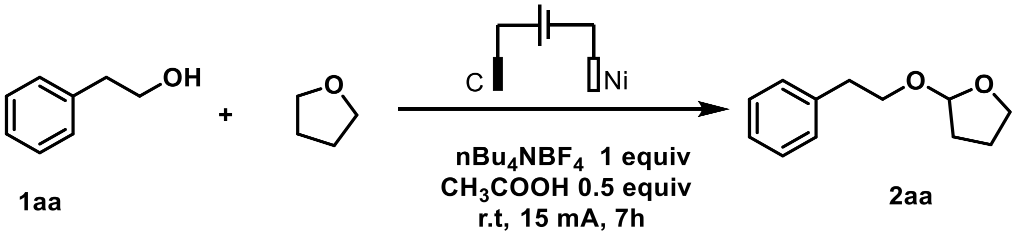

The phenethyl alcohol **1** (0.5 mmol, 1 equiv), nBu_4NBF_4 (0.5 mmol, 1 equiv), CH_3COOH (0.25 mmol, 0.5 equiv), THF (5 mL, 0.1 M) were added into a reaction vial. Then the reaction vial was equipped with a graphite anode and a nickel cathode. And the electrolysis was carried out in air atmosphere at room temperature using a constant current of 15 mA for 7 hours. The reaction solvent was removed under reduced pressure and the crude products were chromatographed through silica gel eluting with ethyl acetate/pentane to give the desired product.

4. Optimization of the reaction conditions

a) Screening of solvents

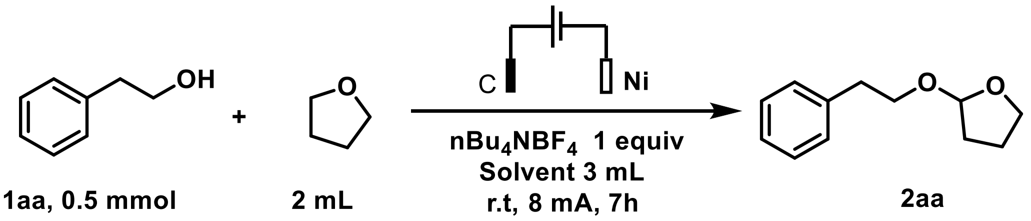

Entry	Solvent	Isolated yield(%)
1	DMA	0
2	Acetone	0
3	CH_3CN	19[a]
4	DCE	0
5	DMF	0
6	CH_2Cl_2	0

[a] graphite as anode.

b) Screening of Electrolytes

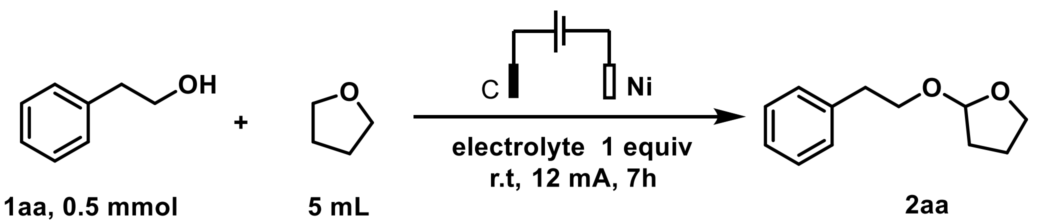

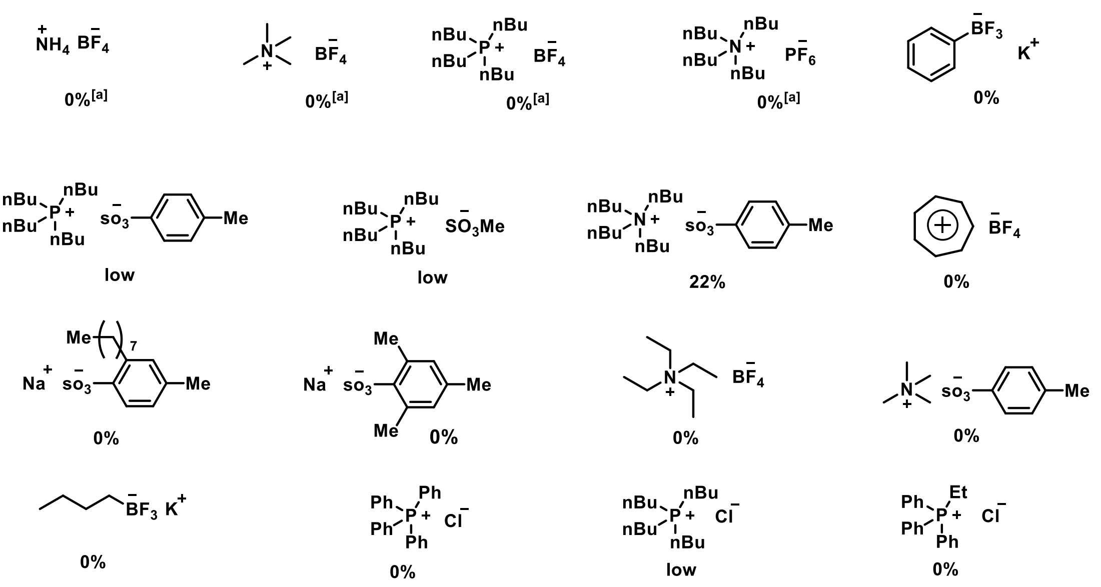

[a] Current was 8 mA

c) Screening of current

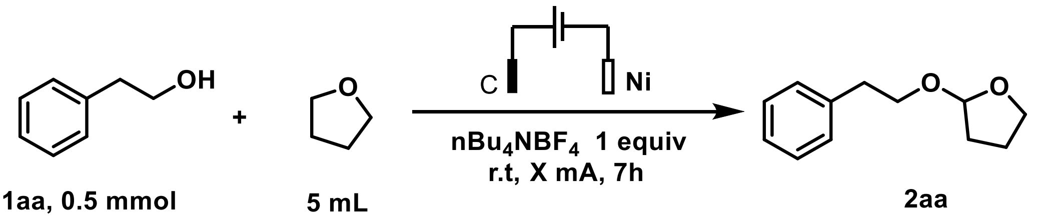

Entry	Current	Isolated yield(%)
1	9	52
2	10	53
3	12	48
4	14	50
5	17	65
6	18	63
7	19	46

5. Product characterization

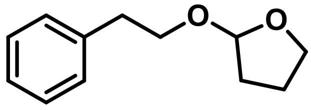

2-phenethoxytetrahydrofuran (2aa)

Colorless oil; Isolated yield: 76%, 73.1 mg. PE:EA = 15:1

^{1}H NMR (400 MHz, $CDCl_3$) δ 7.39 – 7.07 (m, 5H), 5.12 (dd, *J* = 3.8, 2.3 Hz, 1H), 3.99 – 3.75 (m, 3H), 3.61 (dt, *J* = 9.7, 7.2 Hz, 1H), 2.88 (t, *J* = 7.2 Hz, 2H), 2.10 – 1.68 (m, 4H).

^{13}C NMR (101 MHz, $CDCl_3$) δ 139.21, 129.00, 128.34, 126.19, 103.87, 67.99, 66.94, 36.46, 32.45, 23.55 ppm.

ESI-HRMS: $[M+Na]^+$ *m/z*: calculated for $[C_{12}H_{16}O_2Na]^+$ 215.10425, found 215.10400

IR (neat, cm^{-1}): 3062, 3027, 2877, 1602, 1495, 1453, 1350, 1184, 1090, 1034.

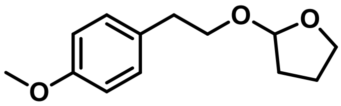

2-(4-methoxyphenethoxy)tetrahydrofuran (2ab)

Yellow oil; Isolated yield: 78%, 86.2 mg. PE:EA = 10:1.

^{1}H NMR (400 MHz, $CDCl_3$) δ 7.14 (d, *J* = 8.6 Hz, 2H), 6.83 (d, *J* = 8.6 Hz, 2H), 5.12 (dd, *J* = 4.0, 2.3 Hz, 1H), 3.89-3.81 (m, 3H), 3.78 (s, 3H), 3.57 (dt, *J* = 9.7, 7.2 Hz, 1H), 2.82 (t, *J* = 7.2 Hz, 2H), 2.06 – 1.73(m, 4H).

^{13}C NMR (101 MHz, $CDCl_3$) δ 158.08, 131.25, 129.91, 113.77, 103.87, 68.27, 66.94, 55.29, 35.54, 32.44, 23.56 ppm.

ESI-HRMS: $[M+Na]^+$ *m/z*: calculated for $[C_{13}H_{18}O_3Na]^+$ 245.11482, found 245.11429.

IR (neat, cm^{-1}): 2940, 1612, 1511, 1460, 1244, 1179, 1091, 1032, 980, 822.

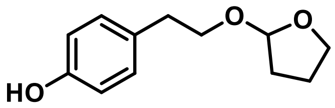

4-(2-((tetrahydrofuran-2-yl)oxy)ethyl)phenol (2ac)

Yellow oil; Isolated yield: 86%, 89.6 mg. PE:EA = 5:1.

^{1}H NMR (400 MHz, $CDCl_3$) δ 7.04 (d, *J* = 8.4 Hz, 2H), 6.72 (d, *J* = 8.4 Hz, 2H), 6.12 (s, 1H), 5.15 (dd, *J* = 3.8, 2.5 Hz, 1H), 3.95 – 3.79 (m, 3H), 3.58 (dt, *J* = 9.6, 7.2 Hz, 1H), 2.79 (t, *J* = 7.3 Hz, 2H), 2.07 – 1.76 (m, 4H).

^{13}C NMR (101 MHz, $CDCl_3$) δ 154.40, 130.78, 130.08, 115.27, 103.96, 68.52, 67.08, 35.46, 32.39, 23.55 ppm.

ESI-HRMS: $[M+Na]^+$ *m/z*: calculated for $[C_{12}H_{16}O_3Na]^+$ 231.09917, found 231.09900.

IR (neat, cm^{-1}): 3344, 2943, 1612, 1513, 1227, 1089, 1027, 978, 918, 827.

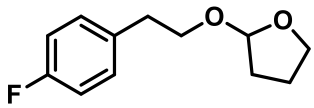

2-(4-fluorophenethoxy)tetrahydrofuran (2ad)

Colorless oil; Isolated yield: 63%, 65.9 mg. PE:EA = 15:1.

^{1}H NMR (400 MHz, $CDCl_3$) δ 7.22 – 7.12 (m, 2H), 7.01– 6.89 (m, 2H), 5.10 (dd, *J* = 4.1, 1.8 Hz, 1H), 3.91 – 3.72 (m, 3H), 3.57 (dt, *J* = 9.7, 7.0 Hz, 1H), 2.84 (t, *J* = 7.0 Hz, 2H), 2.03 – 1.73 (m, 4H).

^{19}F NMR (376 MHz, $CDCl_3$) δ -117.37 – -117.59 (m).

^{13}C NMR (101 MHz, $CDCl_3$) δ 161.59 (d, *J* = 243.8 Hz), 134.98 (d, *J* = 3.0 Hz), 130.40 (d, *J* = 7.6 Hz), 115.10 (d, *J* = 21.1 Hz), 103.91, 67.92, 67.00, 35.64, 32.46, 23.57 ppm.

ESI-HRMS: $[M+Na]^+$ *m/z*: calculated for $[C_{12}H_{15}O_2FNa]^+$ 233.09483, found 233.09483.

IR (neat, cm^{-1}): 2921, 1603, 1509, 1458, 1221, 1093, 1035, 979, 920, 827.

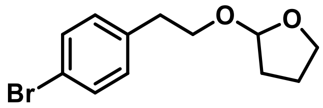

2-(4-bromophenethoxy)tetrahydrofuran (2ae)

Colorless oil; Isolated yield: 51%, 68.8 mg. PE:EA = 15:1.

^{1}H NMR (400 MHz, $CDCl_3$) δ 7.39 (d, *J* = 8.4 Hz, 2H), 7.09 (d, *J* = 8.4 Hz, 2H), 5.09 (dd, *J* = 4.2, 1.7 Hz, 1H), 3.90 – 3.71 (m, 3H), 3.57 (dt, *J* = 9.8, 6.9 Hz, 1H), 2.81 (t, *J* = 6.9 Hz, 2H), 2.02 – 1.73 (m, 4H).

^{13}C NMR (101 MHz, $CDCl_3$) δ 138.36, 131.38, 130.79, 120.01, 103.90, 67.54, 67.01, 35.84, 32.44, 23.56 ppm.

APCI-HRMS: $[M+H]^+$ *m/z*: calculated for $[C_{12}H_{16}O_2{}^{79}Br]^+$ 271.03282, found 271.03360.

IR (neat, cm^{-1}): 2916, 1897, 1486, 1348, 1184, 1092, 1035, 980, 919, 809.

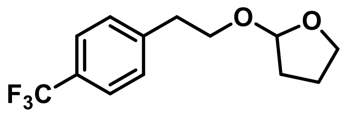

2-(4-(trifluoromethyl)phenethoxy)tetrahydrofuran (2af)

Colorless oil; Isolated yield: 78%, 101 mg. PE:EA = 15:1.

^{1}H NMR (400 MHz, $CDCl_3$) δ 7.53 (d, *J* = 8.0 Hz, 2H), 7.33 (d, *J* = 8.0 Hz, 2H), 5.10 (dd, *J* = 4.2, 1.7 Hz, 1H), 3.97 – 3.72 (m, 3H), 3.62 (dt, *J* = 9.8, 6.8 Hz, 1H), 2.92 (t, *J* = 6.8 Hz, 2H), 2.02 – 1.73 (m, 4H).

^{19}F NMR (376 MHz, $CDCl_3$) δ -62.39.

^{13}C NMR (101 MHz, $CDCl_3$) δ 143.65, 129.34, 128.55 (q, *J* = 32.1 Hz), 125.22 (q, *J* = 3.9 Hz), 124.48 (q, *J* = 272.9 Hz) 103.93, 67.29, 67.03, 36.24, 32.44, 23.54 ppm.

APCI-HRMS: $[M+H]^+$ *m/z*: calculated for $[C_{13}H_{16}O_2F_3]^+$ 261.10969, found 261.11077.

IR (neat, cm^{-1}): 2880, 1802, 1618, 1417, 1323, 1162, 1117, 1064, 1037, 981, 920, 834, 733.

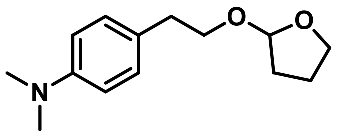

N,N-dimethyl-4-(2-((tetrahydrofuran-2-yl)oxy)ethyl)aniline (2ag)

yellow oil; Isolated yield: 87%, 102.5 mg. PE:EA = 15:1.

^{1}H NMR (400 MHz, $CDCl_3$) δ 7.12 (d, *J* = 8.9 Hz, 2H), 6.72 (d, *J* = 8.7 Hz, 2H), 5.15 (dd, *J* = 3.7, 2.4 Hz, 1H), 3.94 – 3.80 (m, 3H), 3.59 (dt, *J* = 9.6, 7.4 Hz, 1H), 2.93 (s, 6H), 2.82 (t, *J* = 7.4 Hz, 2H), 2.08 – 1.77 (m, 4H).

^{13}C NMR (101 MHz, $CDCl_3$) δ 149.30, 129.55, 127.13, 112.97, 103.86, 68.54, 66.89, 40.92, 35.40, 32.43, 23.56 ppm.

ESI-HRMS: $[M+H]^+$ *m/z*: calculated for $[C_{14}H_{22}O_2N]^+$ 236.16451, found 236.16434.

IR (neat, cm^{-1}): 2877, 2326, 1678, 1517, 1452, 1342, 1118, 1090, 1033, 977, 920, 812, 727.

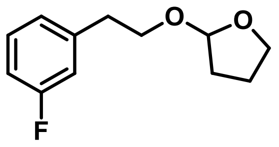

2-(3-fluorophenethoxy)tetrahydrofuran (2ah)

Colorless oil; Isolated yield: 86%, 90.1 mg. PE:EA = 15:1.

^{1}H NMR (400 MHz, $CDCl_3$) δ 7.28-7.15 (m, 1H), 7.01 – 6.82 (m, 3H), 5.09 (dd, *J* = 4.0, 2.1 Hz, 1H), 3.93 – 3.72 (m, 3H), 3.58 (dt, *J* = 9.7, 6.9 Hz, 1H), 2.85 (t, *J* = 6.9 Hz, 2H), 2.02 – 1.72 (m, 4H).

^{19}F NMR (376 MHz, $CDCl_3$) δ -114.01 (td, *J* = 9.6, 6.1 Hz).

^{13}C NMR (101 MHz, $CDCl_3$) δ 162.89 (d, *J* = 245.0 Hz), 141.95 (d, *J* = 7.1 Hz), 129.69 (d, *J* = 8.3 Hz), 124.64 (d, *J* = 2.7 Hz), 115.86 (d, *J* = 21.1 Hz), 113.04 (d, *J* = 21.0 Hz), 103.89, 67.45, 67.00, 36.12 (d, *J* = 1.8 Hz), 32.44, 23.53 ppm.

ESI-HRMS: $[M+Na]^+$ *m/z*: calculated for $[C_{12}H_{15}O_2FNa]^+$ 233.09483, found 233.09467.

IR (neat, cm^{-1}): 2919, 1615, 1449, 1247, 1091, 1035, 918, 863, 781.

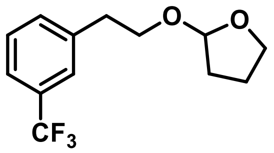

2-(3-(trifluoromethyl)phenethoxy)tetrahydrofuran (2ai)

Colorless oil; Isolated yield: 64%, 83.6 mg. PE:EA = 15:1.

^{1}H NMR (400 MHz, $CDCl_3$) δ 7.52 – 7.35 (m, 4H), 5.10 (dd, *J* = 3.9, 1.9 Hz, 1H), 3.90 (dt, *J* = 9.7, 6.9 Hz, 1H), 3.86 – 3.73 (m, 2H), 3.62 (dt, *J* = 9.7, 6.6 Hz, 1H), 2.92 (t, *J* = 6.8 Hz, 2H), 2.06 – 1.67 (m, 4H).

^{19}F NMR (376 MHz, $CDCl_3$) δ -62.63.

^{13}C NMR (101 MHz, Chloroform-*d*) δ 140.43, 132.45, 130.60 (q, *J* = 31.8 Hz), 128.71, 125.84 (q, *J* = 3.8 Hz), 124.40 (q, *J* = 273.2 Hz), 123.07 (q, *J* = 3.7 Hz), 103.92, 67.29, 67.03, 36.16, 32.45, 23.50.

ESI-HRMS: [M+Na]$^+$ *m/z*: calculated for $[C_{13}H_{15}O_2F_3Na]^+$ 283.09164, found 283.09100.

IR (neat, cm^{-1}): 2919, 1734, 1597, 1488, 1449, 1325, 1163, 1121, 1072, 1036, 981, 797, 702, 660.

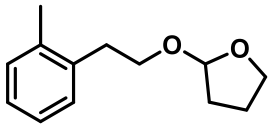

2-(2-methylphenethoxy)tetrahydrofuran (2aj)

Colorless oil; Isolated yield: 82%, 84.4 mg. PE:EA = 15:1.

^{1}H NMR (400 MHz, $CDCl_3$) δ 7.23 – 7.09 (m, 4H), 5.15 (dd, *J* = 4.1, 2.2 Hz, 1H), 3.93 – 3.83 (m, 3H), 3.61 (dt, *J* = 9.8, 7.4 Hz, 1H), 2.91 (t, *J* = 7.5 Hz, 2H), 2.35 (s, 3H), 2.07 – 1.76 (m, 4H).

^{13}C NMR (101 MHz, $CDCl_3$) δ 137.20, 136.48, 130.17, 129.42, 126.36, 125.96, 103.87, 67.02, 66.95, 33.68, 32.48, 23.56, 19.52 ppm.

ESI-HRMS: [M+Na]$^+$ *m/z*: calculated for $[C_{13}H_{18}O_2Na]^+$ 229.11990, found 229.12080.

IR (neat, cm^{-1}): 3305, 2948, 1912, 1662, 1457, 1184, 1091, 1034, 977, 919, 846, 746, 677.

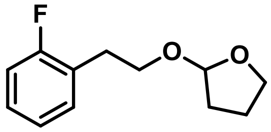

2-(2-fluorophenethoxy)tetrahydrofuran (2ak)

Colorless oil; Isolated yield: 66%, 69.4 mg. PE:EA = 15:1.

^{1}H NMR (400 MHz, $CDCl_3$) δ 7.30 – 7.09 (m, 2H), 7.07 – 6.93(m, 2H), 5.10 (dd, *J* = 4.0, 2.1 Hz, 1H), 3.92 – 3.72 (m, 3H), 3.61 (dt, *J* = 9.7, 7.0 Hz, 1H), 2.90 (t, *J* = 7.1, 2H), 2.04 – 1.65 (m, 4H).

^{19}F NMR (376 MHz, $CDCl_3$) δ -118.60 (q, *J* = 8.1, 7.6 Hz).

^{13}C NMR (101 MHz, $CDCl_3$) δ 161.26 (d, *J* = 245.0 Hz), 131.17 (d, *J* = 5.0 Hz), 127.83 (d, *J* = 8.2 Hz), 125.92 (d, *J* =16.0 Hz), 123.79 (d, *J* = 3.5 Hz), 115.09 (d, *J* = 22.4 Hz), 103.72, 66.85, 66.40 (d, *J* = 1.4 Hz), 32.33, 29.49 (d, *J* = 2.3 Hz), 23.41 ppm.

ESI-HRMS: [M+Na]$^+$ *m/z*: calculated for $[C_{12}H_{15}O_2FNa]^+$ 233.09483, found 233.09479.

IR (neat, cm^{-1}): 2879, 1585, 1491, 1351, 1228, 1182, 1093, 1036, 977, 755

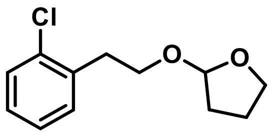

2-(2-chlorophenethoxy)tetrahydrofuran (2al)

Colorless oil; Isolated yield: 42%, 47.8 mg. PE:EA = 15:1.

^{1}H NMR (400 MHz, $CDCl_3$) δ 7.32 (dd, *J* = 7.6, 1.7 Hz, 1H), 7.25 (dd, *J* = 7.2, 2.2 Hz, 1H), 7.14 (pd, *J* = 7.3, 1.8 Hz, 2H), 5.11 (dd, *J* = 4.0, 2.1 Hz, 1H), 3.91 – 3.74 (m, 3H), 3.63 (dt, *J* = 9.8, 7.1 Hz, 1H), 3.00 (t, *J* = 7.1 Hz, 2H), 2.04 – 1.71 (m, 4H).

^{13}C NMR (101 MHz, $CDCl_3$) δ 136.77, 134.33, 131.13, 129.48, 127.75, 126.72, 103.89, 67.01, 66.10, 34.08, 32.48, 23.56 ppm.

ESI-HRMS: $[M+Na]^+$ *m/z*: calculated for $[C_{12}H_{15}O_2ClNa]^+$ 249.06528, found 249.06500.

IR (neat, cm^{-1}): 3063, 2878, 1740, 1571, 1474, 1442, 1349, 1292, 1184, 1119, 1092, 1036, 977, 920, 750.

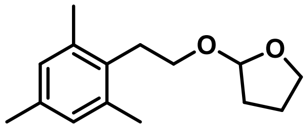

2-(2,4,6-trimethylphenethoxy)tetrahydrofuran (2am)

Colorless oil; Isolated yield: 88%, 102.8 mg. PE:EA = 15:1.

^{1}H NMR (400 MHz, $CDCl_3$) δ 6.87 (s, 2H), 5.18 (dd, *J* = 4.0, 2.1 Hz, 1H), 3.91 (ddd, *J* = 7.4, 6.2, 2.5 Hz, 2H), 3.80 – 3.69 (m, 1H), 3.52 (td, *J* = 9.3, 6.8 Hz, 1H), 2.95 (ddd, *J* = 9.6, 6.6, 2.9 Hz, 2H), 2.36 (s, 6H), 2.29 (s, 3H), 2.11 – 1.79 (m, 4H).

^{13}C NMR (101 MHz, $CDCl_3$) δ 136.77, 135.50, 132.14, 128.92, 103.82, 66.91, 65.86, 32.50, 30.01, 23.55, 20.90, 19.95 ppm.

ESI-HRMS: $[M+Na]^+$ *m/z*: calculated for $[C_{15}H_{22}O_2Na]^+$ 257.15120, found 257.15116

IR (neat, cm^{-1}): 2954, 2912, 1612, 1456, 1348, 1183, 1089, 1035, 919, 850.

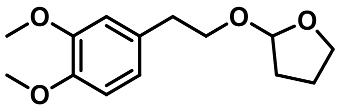

2-(3,4-dimethoxyphenethoxy)tetrahydrofuran (2an)

Yellow oil; Isolated yield: 81%, 102.2 mg. PE:EA = 5:1.

^{1}H NMR (400 MHz, $CDCl_3$) δ 6.81 – 6.68 (m, 3H), 5.09 (dd, *J* = 4.1, 2.1 Hz, 1H), 3.90-3.82 (m, 9H), 3.56 (dt, *J* = 9.7, 7.2 Hz, 1H), 2.79 (t, *J* = 7.2 Hz, 2H), 2.01 – 1.71 (m, 4H).

^{13}C NMR (101 MHz, $CDCl_3$) δ 148.68, 147.38, 131.75, 120.76, 112.23, 111.12, 103.82, 68.15, 66.86, 55.87, 55.77, 35.93, 32.37, 23.49 ppm.

ESI-HRMS: $[M+Na]^+$ *m/z*: calculated for $[C_{14}H_{20}O_4Na]^+$ 275.12538, found 275.12481.

IR (neat, cm^{-1}): 2937, 1590, 1513, 1459, 1343, 1260, 1090, 1031, 984, 918.

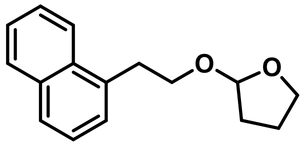

2-(2-(naphthalen-1-yl)ethoxy)tetrahydrofuran (2ao)

Colorless oil; Isolated yield: 67%, 81.5 mg. PE:EA = 15:1.

^{1}H NMR (400 MHz, $CDCl_3$) δ 8.16 – 8.05 (m, 1H), 7.91 – 7.82 (m, 1H), 7.75 (dd, *J* = 7.6, 1.9 Hz, 1H), 7.51 (dddd, *J* = 18.5, 8.1, 6.8, 1.4 Hz, 2H), 7.46 – 7.35 (m, 2H), 5.17 (dd, *J* = 3.5, 2.7 Hz, 1H), 4.04 (ddd, *J* = 9.8, 8.3, 6.7 Hz, 1H), 3.87 (t, *J* = 6.9 Hz, 2H), 3.79 (ddd, *J* = 9.8, 8.0, 6.9 Hz, 1H), 3.46 – 3.29 (m, 2H), 2.09 – 1.75 (m, 4H).

^{13}C NMR (101 MHz, $CDCl_3$) δ 135.11, 133.90, 132.27, 128.80, 127.06, 126.80, 125.96, 125.59, 125.56, 123.93, 104.02, 67.50, 67.04, 33.52, 32.51, 23.57 ppm.

ESI-HRMS: $[M+Na]^+$ *m/z*: calculated for $[C_{16}H_{18}O_2Na]^+$ 265.11990, found 265.11951.

IR (neat, cm^{-1}): 3046, 2878, 2329, 1806, 1396, 1184, 1119, 1090, 1034, 971, 919, 854, 778, 734.

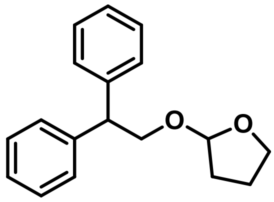

2-(2,2-diphenylethoxy)tetrahydrofuran (2ap)

Colorless oil; Isolated yield: 79%, 105.7 mg. PE:EA = 15:1.

^{1}H NMR (400 MHz, $CDCl_3$) δ 7.36 – 7.15 (m, 10H), 5.15 (dd, *J* = 3.6, 2.0 Hz, 1H), 4.35 – 4.23 (m, 2H), 3.98 – 3.88 (m, 1H), 3.87 – 3.79 (m, 1H), 3.77 – 3.70 (m, 1H), 1.93 – 1.71 (m, 4H).

^{13}C NMR (101 MHz, $CDCl_3$) δ 142.60, 142.46, 128.47, 128.44, 128.43, 128.34, 126.44, 126.37, 103.82, 70.00, 67.02, 51.05, 32.36, 23.46 ppm.

ESI-HRMS: $[M+Na]^+$ *m/z*: calculated for $[C_{18}H_{20}O_2Na]^+$ 291.13555, found 291.13495.

IR (neat, cm^{-1}): 3060, 2882, 1880, 1599, 1492, 1348, 1184, 1093, 1034, 918.

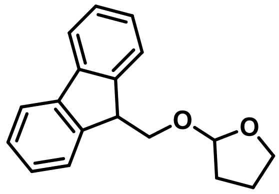

2-((9H-fluoren-9-yl)methoxy)tetrahydrofuran (2aq)

White solid, Isolated yield: 77%, 102.8 mg. PE:EA = 20:1.

^{1}H NMR (400 MHz, $CDCl_3$) δ 7.79 (d, *J* = 7.5 Hz, 2H), 7.71-7.62 (m, 2H), 7.47 – 7.38 (m, 2H), 7.33 (tt, *J* = 7.4, 1.2 Hz, 2H), 5.24 (dd, *J* = 4.8, 1.3 Hz, 1H), 4.20 (t, *J* = 7.5 Hz, 1H), 4.06 (dd, *J* = 9.3, 7.2 Hz, 1H), 4.01 – 3.86 (m, 2H), 3.62 (dd, *J* = 9.3, 8.0 Hz, 1H), 2.19 – 1.82 (m, 4H).

^{13}C NMR (101 MHz, $CDCl_3$) δ 145.39, 144.90, 141.30, 141.28, 127.44, 127.42, 126.92, 126.91, 125.44, 125.22, 119.90, 119.88, 104.18, 69.82, 67.11, 47.99, 32.54, 23.62 ppm.

ESI-HRMS: $[M+Na]^+$ *m/z*: calculated for $[C_{18}H_{18}O_2Na]^+$ 289.11990, found 289.11972.

IR (neat, cm^{-1}): 3038, 2917, 1977, 1740, 1444, 1340, 1192, 1090, 1033, 966, 916, 843, 737.

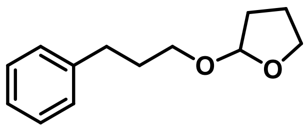

2-(3-phenylpropoxy)tetrahydrofuran (2ar)

Colorless oil; Isolated yield: 87%, 89.5 mg. PE:EA = 15:1.

^{1}H NMR (400 MHz, $CDCl_3$) δ 7.30 (td, *J* = 7.2, 1.7 Hz, 2H), 7.24 – 7.17 (m, 3H), 5.13 (dd, *J* = 4.1, 2.1 Hz, 1H), 3.98-3.84 (m, 2H), 3.71 (dt, *J* = 9.7, 6.5 Hz, 1H), 3.41 (dt, *J* = 9.7, 6.5 Hz, 1H), 2.70 (t, *J* = 8.0 Hz, 2H), 2.10 – 1.77 (m, 6H).

^{13}C NMR (101 MHz, $CDCl_3$) δ 142.12, 128.54, 128.38, 125.81, 103.96, 66.93, 66.55, 32.53, 32.45, 31.48, 23.64 ppm.

ESI-HRMS: $[M+Na]^+$ *m/z*: calculated for $[C_{13}H_{18}O_2Na]^+$ 229.11990, found 229.11987.

IR (neat, cm^{-1}): 3061, 2938, 1889, 1494, 1347, 1092, 1036, 994, 855, 744.

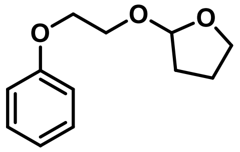

2-(2-phenoxyethoxy)tetrahydrofuran (2as)

Colorless oil; Isolated yield: 89%, 92.7 mg. PE:EA = 10:1.

^{1}H NMR (400 MHz, $CDCl_3$) δ 7.32 – 7.20 (m, 2H), 6.99 – 6.84 (m, 3H), 5.21 (dd, *J* = 3.8, 2.3 Hz, 1H), 4.15-4.04 (m, 2H), 4.00-3.74 (m, 4H), 2.06 – 1.75 (m, 4H).

^{13}C NMR (101 MHz, $CDCl_3$) δ 158.99, 129.52, 120.93, 114.81, 104.29, 67.48, 67.13, 65.64, 32.48, 23.52 ppm.

ESI-HRMS: $[M+Na]^+$ *m/z*: calculated for $[C_{12}H_{16}O_3Na]^+$ 231.09917, found 231.09912

IR (neat, cm^{-1}): 3035, 2927, 2879, 1595, 1493, 1243, 1041, 992, 846, 753.

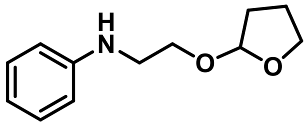

N-(2-((tetrahydrofuran-2-yl)oxy)ethyl)aniline (2at)

yellow oil; Isolated yield: 37%, 38 mg. PE:EA = 8:1.
^{1}H NMR (400 MHz, $CDCl_3$) δ 7.23 – 7.13 (m, 2H), 6.71 (t, J = 7.3 Hz, 1H), 6.67 – 6.58 (m, 2H), 5.16 (dd, J = 4.2, 1.9 Hz, 1H), 4.09 (s, 1H), 3.96 – 3.81 (m, 3H), 3.67 (ddd, J = 10.4, 6.2, 4.4 Hz, 1H), 3.36-3.21 (m, 2H), 2.09 – 1.77 (m, 4H).

^{13}C NMR (101 MHz, $CDCl_3$) δ 148.44, 129.32, 117.60, 113.18, 104.28, 67.17, 66.05, 43.98, 32.48, 23.62 ppm.

ESI-HRMS: $[M+H]^+$ m/z: calculated for $[C_{12}H_{18}O_2N]^+$ 208.13321, found 208.13290.

IR (neat, cm^{-1}): 3051, 2921, 1915, 1602, 1460, 1320, 1182, 1095, 1038, 918.

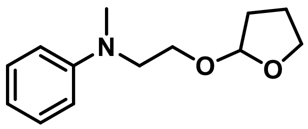

N-methyl-N-(2-((tetrahydrofuran-2-yl)oxy)ethyl)aniline (2au)

yellow oil; Isolated yield: 71%, 78.4 mg. PE:EA = 10:1.

^{1}H NMR (400 MHz, $CDCl_3$) δ 7.26 – 7.18 (m, 2H), 6.78 – 6.66 (m, 3H), 5.10 (dd, J = 4.0, 2.0 Hz, 1H), 3.93 – 3.76 (m, 3H), 3.64 – 3.43 (m, 3H), 2.97 (s, 3H), 2.05 – 1.76 (m, 4H).

^{13}C NMR (101 MHz, $CDCl_3$) δ 149.35, 129.20, 116.24, 112.19, 104.25, 67.03, 64.53, 52.61, 38.85, 32.52, 23.51 ppm.

ESI-HRMS: $[M+H]^+$ m/z: calculated for $[C_{13}H_{20}O_2N]^+$ 222.14886, found 222.14859.

IR (neat, cm^{-1}): 3026, 2880, 2325, 1598, 1504, 1452, 1363, 1189, 1094, 1037, 989, 919, 747, 691.

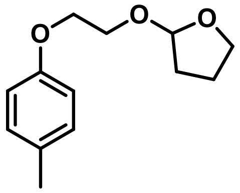

2-(2-(p-tolyloxy)ethoxy)tetrahydrofuran (2av)

Colorless oil; Isolated yield: 89%, 98.4 mg. PE:EA = 8:1.

^{1}H NMR (400 MHz, $CDCl_3$) δ 7.12 – 7.05 (m, 2H), 6.83 (d, *J* = 8.5 Hz, 2H), 5.23 (dd, *J* = 4.2, 1.9 Hz, 1H), 4.14 – 4.04 (m, 2H), 4.01 – 3.85 (m, 3H), 3.84 – 3.75 (m, 1H), 2.29 (s, 3H), 2.07 – 1.76 (m, 4H).

^{13}C NMR (101 MHz, $CDCl_3$) δ 156.83, 129.89, 114.61, 104.21, 67.59, 67.03, 65.63, 32.41, 23.46, 20.53.

ESI-HRMS: $[M+Na]^+$ *m/z*: calculated for $[C_{13}H_{18}O_3Na]^+$ 245.11482, found 245.11432

IR (neat, cm^{-1}): 2924, 2877, 1875, 1613, 1510, 1348, 1289, 1180, 1104, 1992, 919, 813, 740.

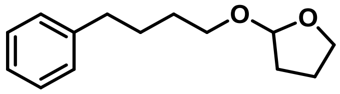

2-(4-phenylbutoxy)tetrahydrofuran (2aw)

Colorless oil; Isolated yield: 91%, 100 mg. PE:EA = 15:1.

^{1}H NMR (400 MHz, $CDCl_3$) δ 7.33-7.27 (m, 2H), 7.24-7.17 (m, 3H), 5.12 (dd, *J* = 4.3, 1.9 Hz, 1H), 3.89 (dt, *J* = 7.6, 6.3 Hz, 2H), 3.71 (dt, *J* = 9.6, 6.5 Hz, 1H), 3.46 – 3.37 (m, 1H), 2.66 (t, *J* = 7.5 Hz, 2H), 2.07 – 1.78 (m, 4H), 1.75 – 1.57 (m, 4H).
^{13}C NMR (101 MHz, $CDCl_3$) δ 142.59, 128.49, 128.33, 125.74, 103.86, 67.07, 66.86, 35.76, 32.40, 29.46, 28.19, 23.60 ppm.

ESI-HRMS: $[M+Na]^+$ *m/z*: calculated for $[C_{14}H_{20}O_2Na]^+$ 243.13555, found 243.13522.

IR (neat, cm^{-1}): 3026, 2933, 2325, 1602, 1453, 1349, 1092, 1037, 919, 743, 698.

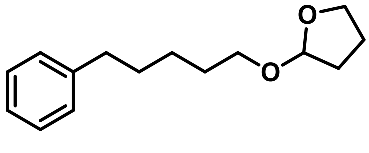

2-((5-phenylpentyl)oxy)tetrahydrofuran (2ax)

Colorless oil; Isolated yield: 91%, 107 mg. PE:EA = 15:1.

^{1}H NMR (400 MHz, $CDCl_3$) δ 7.31-7.23 (m, 2H), 7.21 – 7.14 (m, 3H), 5.10 (dd, *J* = 4.4, 1.8 Hz, 1H), 3.86 (dt, *J* = 7.7, 5.8 Hz, 2H), 3.66 (dt, *J* = 9.6, 6.8 Hz, 1H), 3.37 (dt, *J* = 9.6, 6.6 Hz, 1H), 2.62 (t, *J* = 8.0 Hz, 2H), 2.05 – 1.75 (m, 4H), 1.70 – 1.55 (m, 4H), 1.44 – 1.34 (m, 2H).

^{13}C NMR (101 MHz, $CDCl_3$) δ 142.75, 128.46, 128.31, 125.68, 103.84, 67.19, 66.84, 35.99, 32.41, 31.37, 29.68, 25.95, 23.60 ppm.

ESI-HRMS: $[M+Na]^+$ *m/z*: calculated for $[C_{15}H_{22}O_2Na]^+$ 257.15120, found 257.15107.

IR (neat, cm^{-1}): 3061, 2930, 2860, 1494, 1349, 1093, 1037, 919, 852, 741.

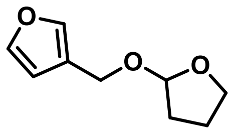

3-(((tetrahydrofuran-2-yl)oxy)methyl)furan (2ay)

Colorless oil; Isolated yield: 59%, 49.8 mg. PE:EA = 15:1.

^{1}H NMR (400 MHz, $CDCl_3$) δ 7.44-7.36 (m, 2H), 6.44 – 6.36 (m, 1H), 5.19 (dd, *J* = 4.0, 2.3 Hz, 1H), 4.55 (dd, *J* = 12.0, 0.9 Hz, 1H), 4.36 (d, *J* = 12.0 Hz, 1H), 3.98 – 3.82 (m, 2H), 2.08 – 1.75 (m, 4H).

^{13}C NMR (101 MHz, $CDCl_3$) δ 143.38, 140.79, 122.34, 110.60, 102.89, 67.11, 60.15, 32.42, 23.58 ppm.

IR (neat, cm^{-1}): 2883, 2326, 1769, 1458, 1185, 1086, 1026, 964, 919, 871, 791, 729.

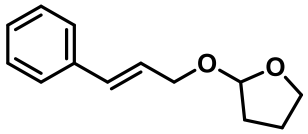

2-(cinnamyloxy)tetrahydrofuran (2az)

Colorless oil; Isolated yield: 43%, 44.1 mg. PE:EA = 15:1.

^{1}H NMR (400 MHz, $CDCl_3$) δ 7.40 – 7.35 (m, 2H), 7.29 (dd, *J* = 8.3, 6.6 Hz, 2H), 7.25 – 7.18 (m, 1H), 6.60 (d, *J* = 16.0 Hz, 1H), 6.34-6.22 (m, 1H), 5.26 (t, *J* = 3.1 Hz, 1H), 4.33 (ddd, *J* = 12.8, 5.7, 1.6 Hz, 1H), 4.12 (ddd, *J* = 12.8, 6.6, 1.4 Hz, 1H), 3.99-3.83 (m, 2H), 2.09 – 1.76 (m, 4H).

^{13}C NMR (101 MHz, $CDCl_3$) δ 136.89, 132.41, 128.61, 127.70, 126.59, 126.16, 103.28, 67.70, 67.13, 32.49, 23.61 ppm.

EI-HRMS: $[M]^+$ *m/z*: calculated for $[C_{13}H_{16}O_2]^+$ 204.11448, found 204.11370.

IR (neat, cm^{-1}): 3027, 2926, 2324, 1184, 1083, 1027, 966, 918, 852, 740, 693.

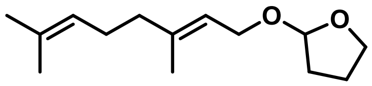

(E)-2-((3,7-dimethylocta-2,6-dien-1-yl)oxy)tetrahydrofuran (2ba)

Colorless oil; Isolated yield: 69%, 77.6 mg. PE:EA = 40:1.

^{1}H NMR (400 MHz, $CDCl_3$) δ 5.35 – 5.29 (m, 1H), 5.14 (dd, *J* = 4.4, 2.1 Hz, 1H), 5.08 (tt, *J* = 6.9, 1.5 Hz, 1H), 4.16 (dd, *J* = 11.8, 6.5 Hz, 1H), 3.96 (dd, *J* = 11.8, 7.3 Hz, 1H), 3.92 – 3.82 (m, 2H), 2.13 – 1.76 (m, 8H), 1.66 (d, *J* = 1.5 Hz, 6H), 1.58 (d, *J* = 1.4 Hz, 3H).

^{13}C NMR (101 MHz, $CDCl_3$) δ 140.38, 131.68, 124.14, 120.65, 103.06, 66.93, 63.58, 39.73, 32.45, 26.48, 25.78, 23.62, 17.77, 16.51 ppm.

ESI-HRMS: $[M+Na]^+$ *m/z*: calculated for $[C_{14}H_{24}O_2Na]^+$ 247.16685, found 247.16672.

IR (neat, cm^{-1}): 2919, 1669, 1447, 1375, 1119, 1082, 1024, 920, 837, 741.

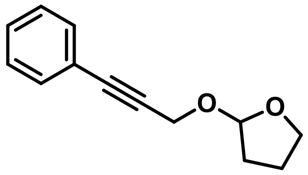

2-((3-phenylprop-2-yn-1-yl)oxy)tetrahydrofuran (2bb)

Colorless oil; Isolated yield: 45%, 45.5 mg. PE:EA = 20:1.

^{1}H NMR (400 MHz, $CDCl_3$) δ 7.45 (dd, *J* = 6.7, 3.0 Hz, 2H), 7.30 (dd, *J* = 5.1, 1.9 Hz, 3H), 5.38 (t, *J* = 3.0 Hz, 1H), 4.51 – 4.37 (m, 2H), 3.99 – 3.86 (m, 2H), 2.10 – 1.94 (m, 3H), 1.92 – 1.81 (m, 1H).

^{13}C NMR (101 MHz, $CDCl_3$) δ 131.81, 128.35, 128.21, 122.70, 102.31, 85.62, 85.15, 67.24, 54.56, 32.30, 23.31.

APCI-HRMS: $[M+H]^+$ *m/z*: calculated for $[C_{13}H_{15}O_2]^+$ 203.10666, found 203.10754

IR (neat, cm^{-1}): 2327, 1886, 1489, 1346, 1115, 1085, 1031, 960, 918, 840, 756, 691.

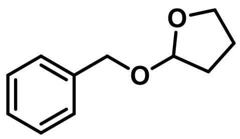

2-(benzyloxy)tetrahydrofuran (2bc)

Colorless oil; Isolated yield: 41%, 36.3 mg. PE:EA = 15:1.

^{1}H NMR (400 MHz, $CDCl_3$) δ 7.37 – 7.20 (m, 5H), 5.21 (dd, *J* = 4.1, 2.1 Hz, 1H), 4.71 (d, *J* = 11.8 Hz, 1H), 4.47 (d, *J* = 11.9 Hz, 1H), 4.01-3.84 (m, 2H), 2.09 – 1.77 (m, 4H).

^{13}C NMR (101 MHz, $CDCl_3$) δ 138.47, 128.48, 127.98, 127.61, 103.22, 68.89, 67.14, 32.47, 23.59.

ESI-HRMS: $[M+Na]^+$ *m/z*: calculated for $[C_{11}H_{14}O_2Na]^+$ 201.08860, found 201.08842.

IR (neat, cm^{-1}): 3063, 2882, 1808, 1454, 1348, 1184, 1088, 1032, 963, 919, 862, 735, 697.

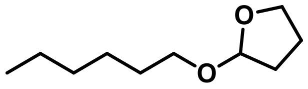

2-(hexyloxy)tetrahydrofuran (2bd)

Colorless oil; Isolated yield: 48%, 41.5 mg. PE:EA = 40:1.

^{1}H NMR (400 MHz, $CDCl_3$) δ 5.09 (dd, *J* = 4.4, 1.7 Hz, 1H), 3.94-3.78 (m, 2H), 3.63 (dt, *J* = 9.6, 6.9 Hz, 1H), 3.35 (dt, *J* = 9.5, 6.7 Hz, 1H), 2.04 – 1.74 (m, 4H), 1.60 – 1.48 (m, 2H), 1.38 – 1.18 (m, 6H), 0.93 – 0.79 (m, 3H).

^{13}C NMR (101 MHz, $CDCl_3$) δ 103.90, 67.45, 66.88, 32.45, 31.78, 29.85, 26.00, 23.66, 22.76, 14.17 ppm.

ESI-HRMS: $[M+Na]^+$ *m/z*: calculated for $[C_{10}H_{20}O_2Na]^+$ 195.13555, found 195.13557.

IR (neat, cm^{-1}): 2927, 2863, 1740, 1459, 1186, 1115, 1091, 1037, 920, 856, 727.

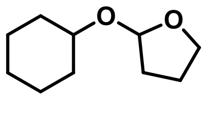

2-(cyclohexyloxy)tetrahydrofuran (2be)

Colorless oil; Isolated yield: 64%, 54.3 mg. PE:EA = 40:1.

^{1}H NMR (400 MHz, $CDCl_3$) δ 5.27 (dd, *J* = 4.8, 1.6 Hz, 1H), 3.92-3.78 (m, 2H), 3.56-3.45(m, 1H), 2.06 – 1.61 (m, 8H), 1.35 – 1.05 (m, 6H).

^{13}C NMR (101 MHz, $CDCl_3$) δ 101.80, 74.71, 66.64, 34.09, 32.71, 32.17, 25.86, 24.61, 24.47, 23.69 ppm.

ESI-HRMS: $[M+Na]^+$ *m/z*: calculated for $[C_{10}H_{18}O_2Na]^+$ 193.11990, found 193.11985.

IR (neat, cm^{-1}): 2929, 2857, 1739, 1450, 1186, 1115, 1088, 1033, 920, 859, 795, 751.

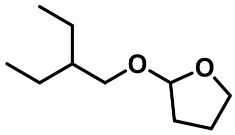

2-(2-ethylbutoxy)tetrahydrofuran (2bf)

Colorless oil; Isolated yield: 57%, 49.4 mg. PE:EA = 40:1.

^{1}H NMR (400 MHz, $CDCl_3$) δ 5.07 (dd, *J* = 4.2, 1.9 Hz, 1H), 3.85 (ddd, *J* = 7.4, 6.1, 3.8 Hz, 2H), 3.55 (dd, *J* = 9.5, 6.1 Hz, 1H), 3.23 (dd, *J* = 9.5, 5.6 Hz, 1H), 2.06 – 1.74 (m, 4H), 1.45 – 1.23 (m, 5H), 0.86 (t, *J* = 7.4 Hz, 6H).

^{13}C NMR (101 MHz, $CDCl_3$) δ 103.99, 69.57, 66.82, 41.17, 32.42, 23.67, 23.52, 23.44, 11.21, 11.09 ppm.

ESI-HRMS: $[M+Na]^+$ *m/z*: calculated for $[C_{10}H_{20}O_2Na]^+$ 195.13555, found 195.13550.

IR (neat, cm^{-1}): 2960, 2875, 1460, 1351, 1186, 1116, 1089, 1035, 981, 919, 860, 774.

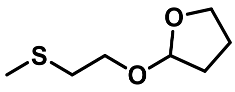

2-(2-(methylthio)ethoxy)tetrahydrofuran (2bg)

Colorless oil; Isolated yield: 38%, 30.6 mg. PE:EA = 40:1.

^{1}H NMR (400 MHz, $CDCl_3$) δ 5.14 (dd, *J* = 4.2, 1.9 Hz, 1H), 3.96 – 3.76 (m, 3H), 3.58 (dt, *J* = 10.2, 6.9 Hz, 1H), 2.67 (t, *J* = 6.7 Hz, 2H), 2.13 (s, 3H), 2.03 – 1.76 (m, 4H).

^{13}C NMR (101 MHz, $CDCl_3$) δ 104.06, 67.07, 66.53, 33.92, 32.49, 23.52, 16.04 ppm.

ESI-HRMS: $[M+Na]^+$ *m/z*: calculated for $[C_7H_{14}O_2NaS]^+$ 185.06067, found 185.06140.

IR (neat, cm^{-1}): 2917, 1782, 1438, 1184, 1118, 1091, 1032, 986, 920, 856, 697.

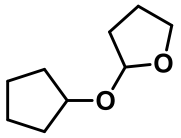

2-(cyclopentyloxy)tetrahydrofuran (2bh)

Yellow oil, 31.2 mg, 40% yield, PE : EA = 50:1

^{1}H NMR (400 MHz, Chloroform-*d*) δ 5.18 (dd, *J* = 4.7, 1.6 Hz, 1H), 4.18 – 4.08 (m, 1H), 3.92-3.77 (m, 2H), 2.05 – 1.43 (m, 12H).

^{13}C NMR (101 MHz, Chloroform-*d*) δ 102.77, 78.44, 66.72, 33.61, 32.68, 32.08, 23.71, 23.60, 23.42.

ESI-HRMS: $[M+Na]^+$*m/z*: calculated for$[C_9H_{16}O_2Na]^+$ 179.10425, found 179.10392.

IR (neat, cm^{-1}): 2952, 2089, 1451, 1339, 1175, 1119, 1074, 1019, 919, 858.

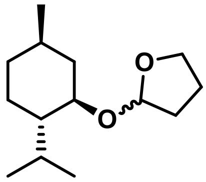

2-(((1R,2S,5R)-2-isopropyl-5-methylcyclohexyl)oxy)tetrahydrofuran (2bi), *dr* = 1.6:1.

Light yellow oil, 76.1 mg, 67% yield, PE : EA = 50:1

^{1}H NMR (400 MHz, Chloroform-*d*) δ 5.29 (d, *J* = 3.6 Hz, 1H of major dia), 5.18 (t, *J* = 3.2 Hz, 1H of minor dia), 3.99– 3.75 (m, 2H of both dias), 3.43 (td, *J* = 10.6 Hz, *J* = 4.2 Hz, 1H of major dia), 3.27 (td, *J* = 10.6 Hz, *J* = 4.4 Hz, 1H of minor dia), 2.20 – 1.92 (m, 3H of both dias), 1.93 – 1.72 (m, 3H of both dias), 1.69-1.60 (m, 2H of both dias), 1.46 – 0.68 (m, 14H of both dias).

^{13}C NMR (101 MHz, Chloroform-*d*) δ 105.47, 99.50, 78.80, 73.70, 66.85, 66.65, 48.87, 48.20, 43.60, 40.18, 34.76, 34.58, 32.73, 32.67, 31.83, 31.55, 25.73, 25.51, 23.76, 23.64, 23.46, 23.29, 22.48, 22.40, 21.26, 21.17, 16.41, 15.66.

ESI-HRMS: [M+Na]$^+$*m/z*: calculated for[$C_{14}H_{26}O_2Na$]$^+$ 249.18250, found 249.18208.

IR (neat, cm^{-1}): 2922, 2088, 1454, 1367, 1182, 1078, 1027, 917, 845, 758.

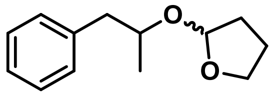

2-((1-phenylpropan-2-yl)oxy)tetrahydrofuran (2bj), *dr* = 1:1.

Colorless oil, 47 mg, 46% yield, PE : EA = 50:1

^{1}H NMR (400 MHz, Chloroform-*d*) δ 7.32 – 7.11 (m, 5H of both dias), 5.21 (m, 1H of one dia), 5.00 (m, 1H of other dia), 4.04 – 3.75 (m, 2H of both dias), 3.64 (m, 1H of one or other dia), 3.32 (m, 1H of other or one dia), 2.90 – 2.55 (m, 2H of both dias), 2.08 – 1.63 (m, 4H of both dias), 1.17 (d, *J* = 6.2 Hz, 3H of one or other dia), 1.12 (d, *J* = 6.2 Hz, 3H of other or one dia).

^{13}C NMR (101 MHz, Chloroform-*d*) δ 139.49, 139.19, 129.64, 129.60, 128.26, 128.09, 126.14, 125.99, 103.45, 100.83, 74.53, 71.89, 66.71, 66.49, 44.14, 43.35, 32.64, 32.47, 23.55, 23.40, 21.76, 19.25.

ESI-HRMS: [M+Na]$^+$*m/z*: calculated for[$C_{13}H_{18}O_2Na$]$^+$ 229.11990, found 229.11958.

IR (neat, cm^{-1}): 2926, 2088, 1494, 1184, 1086, 1004, 917, 847, 742.

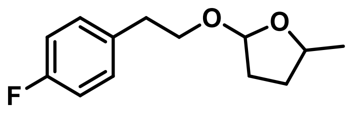

2-(4-fluorophenethoxy)-5-methyltetrahydrofuran (3ad & 3ad', in total 41% isolated yield).

Major product: **3ad**, isolated yield: 31%, 35mg. colorless oil. PE:EA = 50:1.

3ad: **^{1}H NMR** (400 MHz, $CDCl_3$) δ 7.22 – 7.09 (m, 2H), 6.99 – 6.83 (m, 2H), 5.09 (dd, *J* = 5.1, 1.9 Hz, 1H), 4.13-4.02 (m, 1H), 3.85 (dt, *J* = 9.8, 7.1 Hz, 1H), 3.55 (dt, *J* = 9.7, 7.0 Hz, 1H), 2.82 (t, *J* = 7.0 Hz, 2H), 2.10 – 1.95 (m, 2H), 1.86 – 1.75 (m, 1H), 1.40 – 1.28 (m, 1H), 1.19 (d, *J* = 6.1 Hz, 3H).

^{19}F NMR (376 MHz, $CDCl_3$) δ -117.51.

^{13}C NMR (101 MHz, $CDCl_3$) δ 161.43 (d, *J* = 243.5 Hz), 134.83 (d, *J* = 3.4 Hz), 130.24 (d, *J* = 7.5 Hz), 114.93 (d, *J* = 21.0 Hz), 104.05, 73.83, 67.88, 35.50, 32.43, 31.16, 20.80.

IR (neat, cm^{-1}): 2922, 2870, 1603, 1509, 1341, 1222, 1096, 1062, 1006, 952, 891, 826, 760, 703.

Minor product: **3ad'**, isolated yield: 10%, 11 mg. colorless oil.

3ad': **^{1}H NMR** (600 MHz, $CDCl_3$) δ 7.21 – 7.13 (m, 2H), 7.01 – 6.91 (m, 2H), 5.02 (d, *J* = 4.8 Hz, 1H), 4.19-4.12 (m, 1H), 3.87 (dt, *J* = 9.7, 7.1 Hz, 1H), 3.55 (dt, *J* = 9.6, 6.9 Hz, 1H), 2.84 (t, *J* = 7.0 Hz, 2H), 1.99-1.92 (m, 2H), 1.92 – 1.85 (m, 1H), 1.65 – 1.57 (m, 1H), 1.21 (d, *J* = 6.2 Hz, 3H).

^{19}F NMR (565 MHz, $CDCl_3$) δ -117.54.

^{13}C NMR (151 MHz, $CDCl_3$) δ 161.62 (d, *J* = 243.5 Hz), 135.12 (d, *J* = 3.4 Hz), 130.47 (d, *J* = 7.8 Hz), 115.08 (d, *J* = 20.8 Hz), 103.93, 76.75, 67.60, 35.70, 33.77, 31.23, 22.95.

IR (neat, cm^{-1}): 2924, 2868, 1602, 1509, 1346, 1221, 1096, 1059, 990, 891, 828, 706.

6. Intermolecular Competing Kinetic Isotope Effect

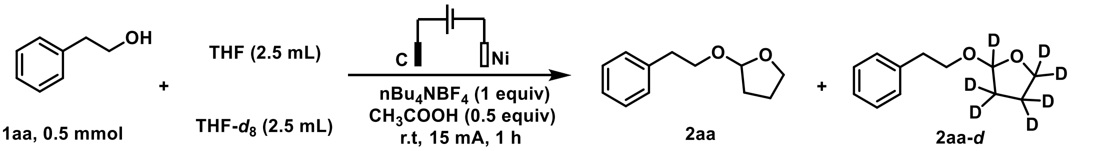

1aa, 0.5 mmol   2aa   2aa-*d*

Following general procedure, Phenethyl alcohol (61.1 mg, 1 equiv), nBu_4NBF_4 (164.6 mg, 1 equiv), CH_3COOH (15 mg, 0.5 equiv), THF (2.5 mL) and THF-d_8 (2.5 mL) were added into a reaction vial. The reaction was carried out in air atmosphere at room temperature for 1 h. Then, the solvent was removed and the crude products were chromatographed through silica gel eluting to give desired products 2 and 2-*d* in a ratio of 60:40, the ratio was detected via ^{1}H NMR shown below.

KIE = K_H/K_D= 0.6/(1-0.6) = 1.5.

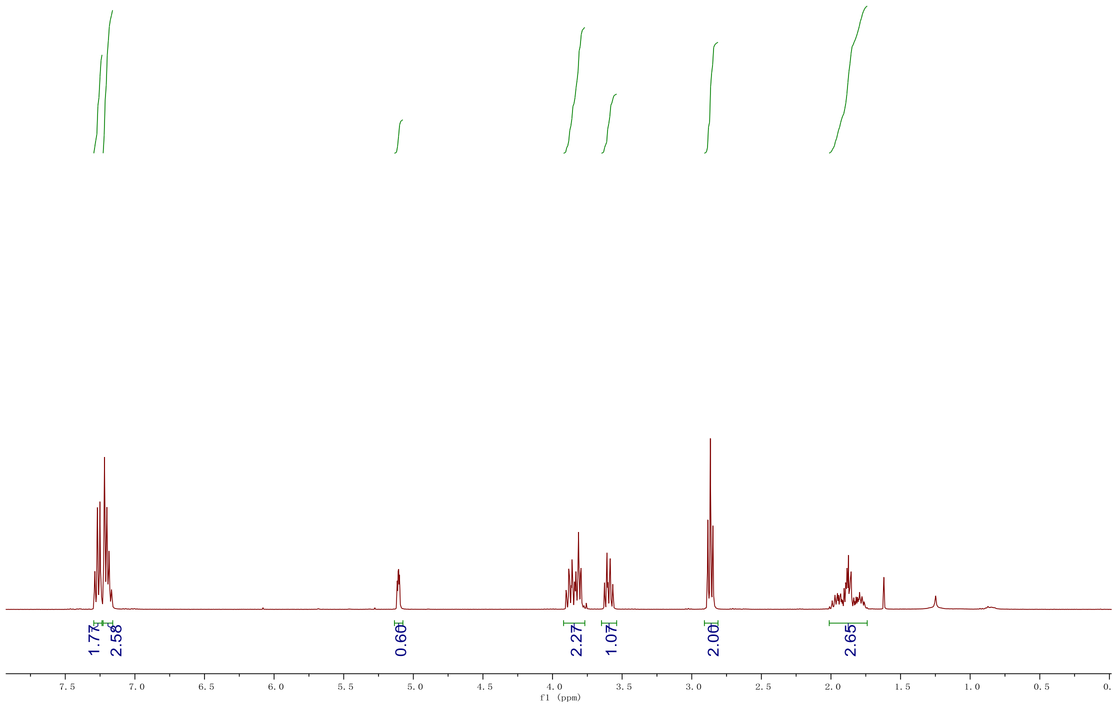
1.77
2.58
0.60
2.27
1.07
2.00
2.65
7.5
7.0
6.5
6.0
5.5
5.0
4.5
4.0
3.5
3.0
2.5
2.0
1.5
1.0
0.5
f1 (ppm)

7. NMR Spectra for Products

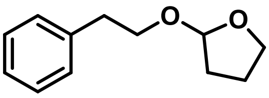

2aa

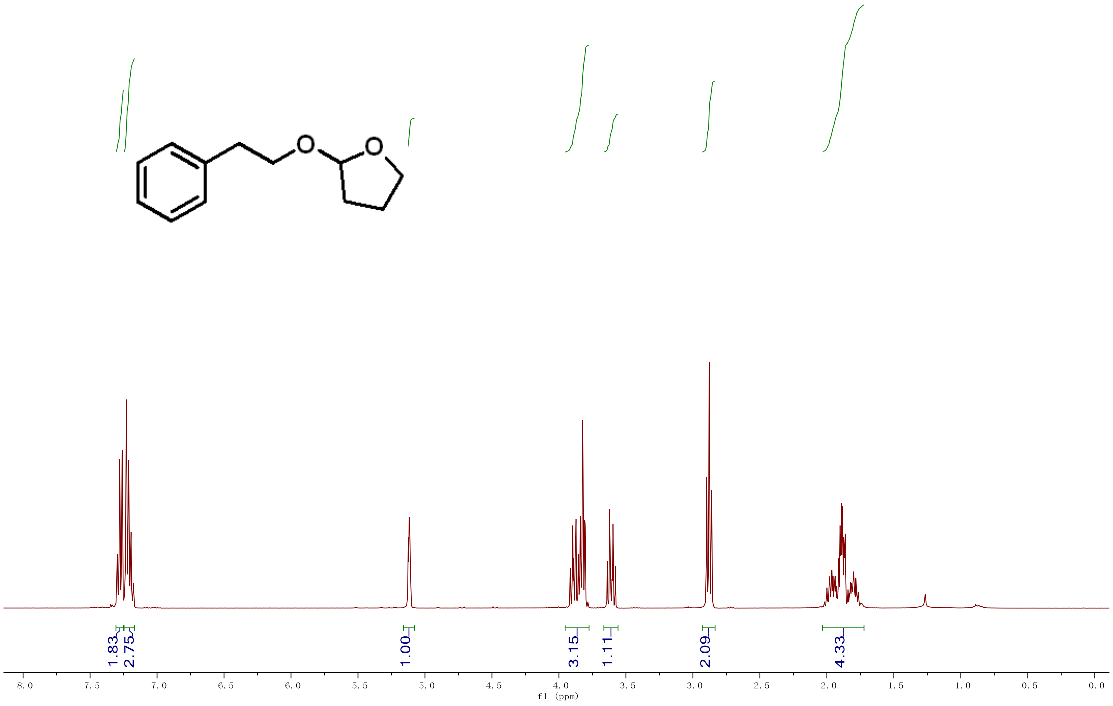

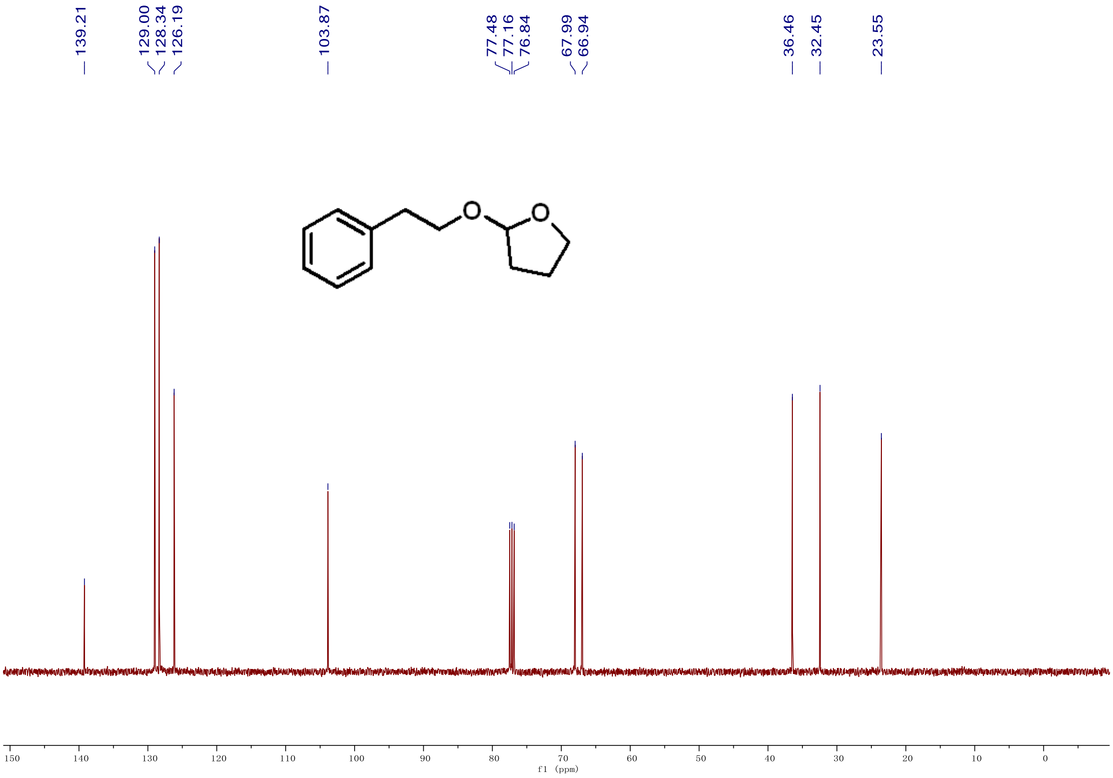

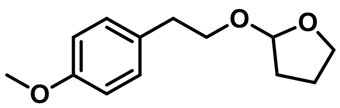

2ab

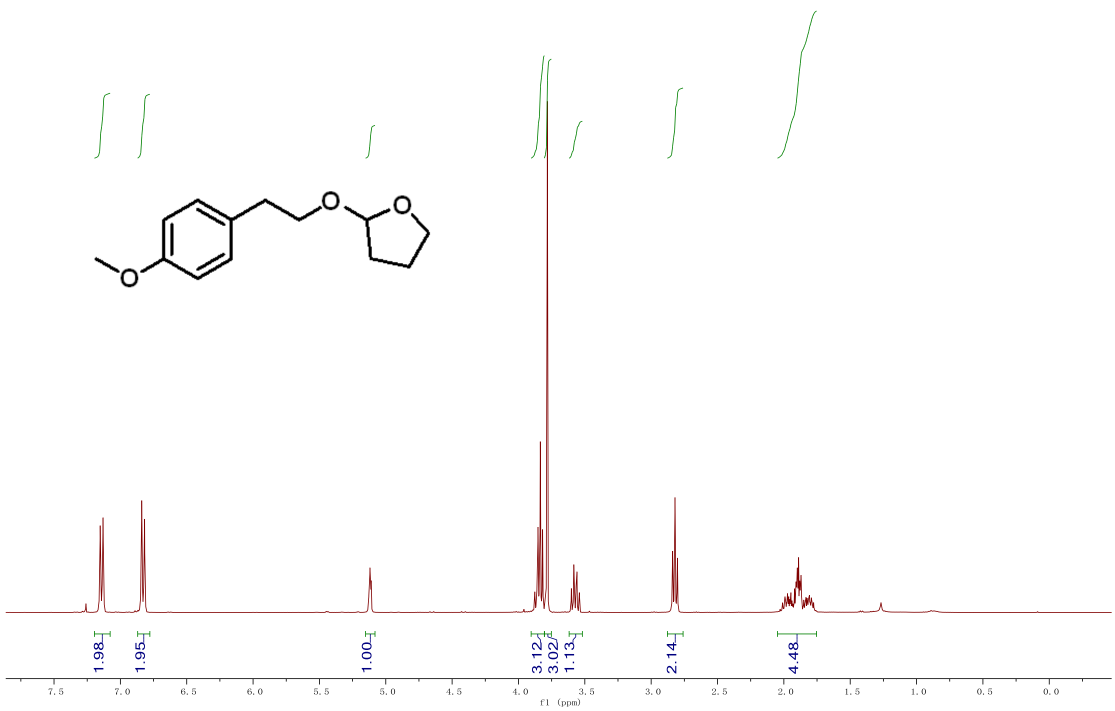

1.98
1.95
1.00
3.12
3.02
1.13
2.14
4.48
7.5 7.0 6.5 6.0 5.5 5.0 4.5 4.0 3.5 3.0 2.5 2.0 1.5 1.0 0.5 0.0
f1 (ppm)

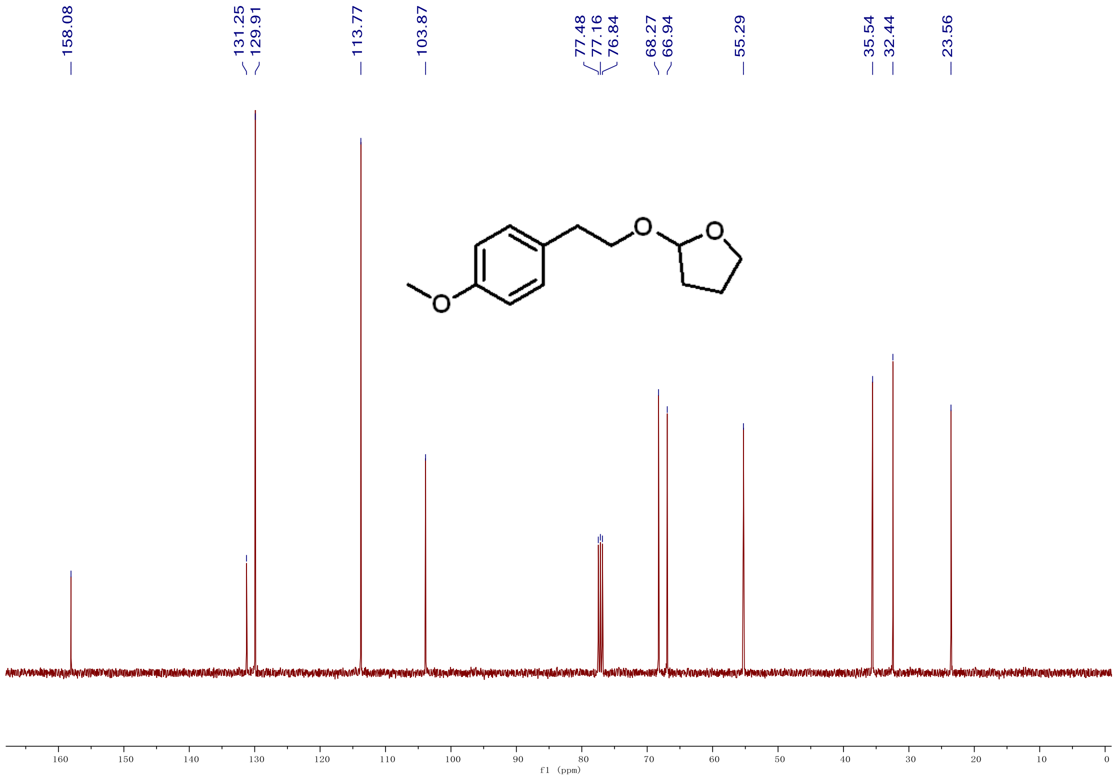

158.08
131.25
129.91
113.77
103.87
77.48
77.16
76.84
68.27
66.94
55.29
35.54
32.44
23.56
160 150 140 130 120 110 100 90 80 70 60 50 40 30 20 10 0
f1 (ppm)

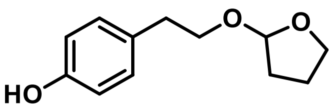

2ac

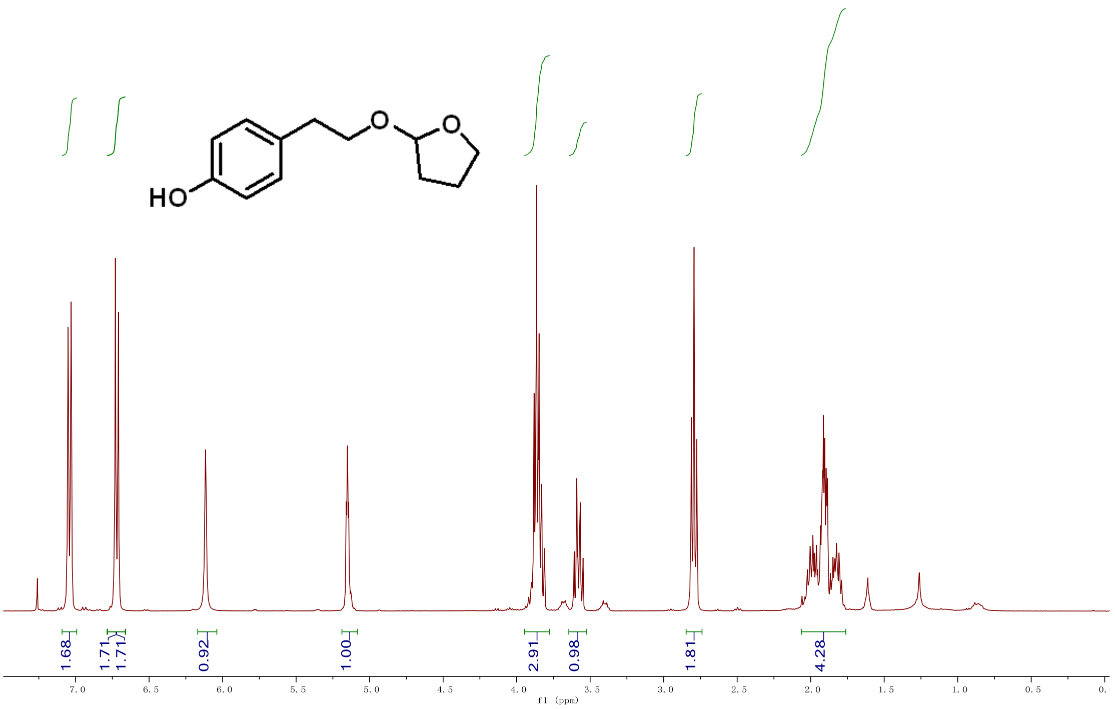

HO
O
O
1.68
1.71
1.71
0.92
1.00
2.91
0.98
1.81
4.28
7.0
6.5
6.0
5.5
5.0
4.5
4.0
3.5
3.0
2.5
2.0
1.5
1.0
0.5
f1 (ppm)

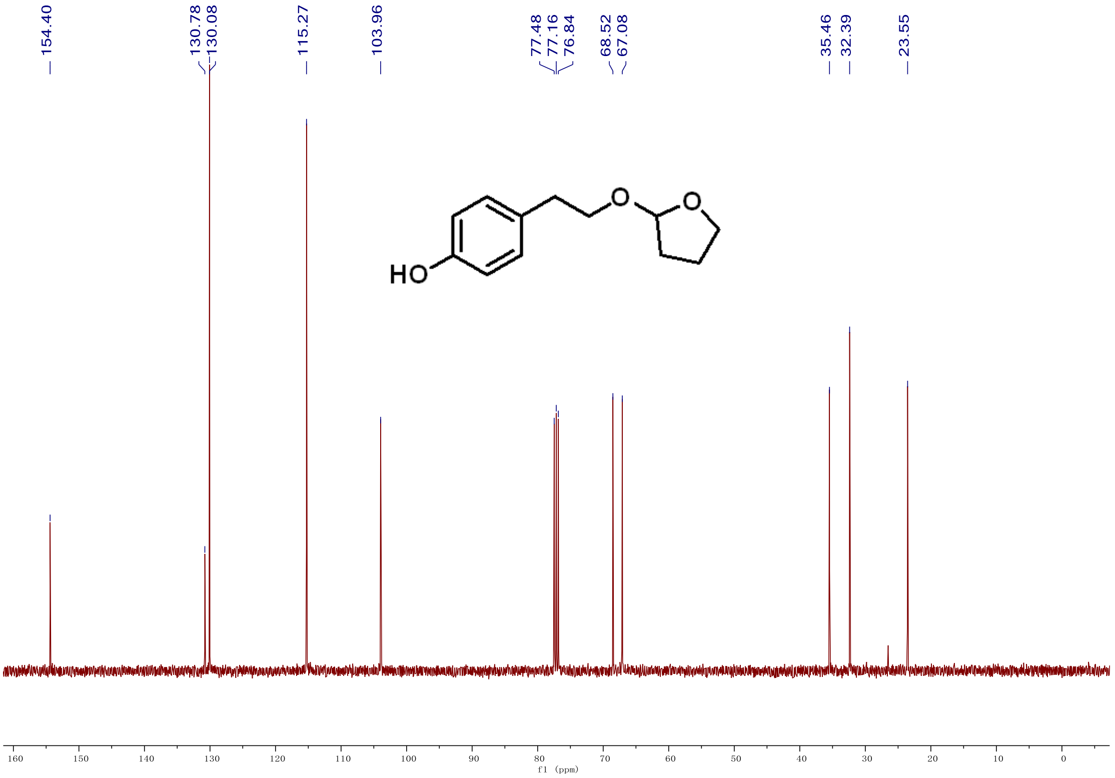

154.40
130.78
130.08
115.27
103.96
77.48
77.16
76.84
68.52
67.08
35.46
32.39
23.55
HO
O
O
160
150
140
130
120
110
100
90
80
70
60
50
40
30
20
10
0
f1 (ppm)

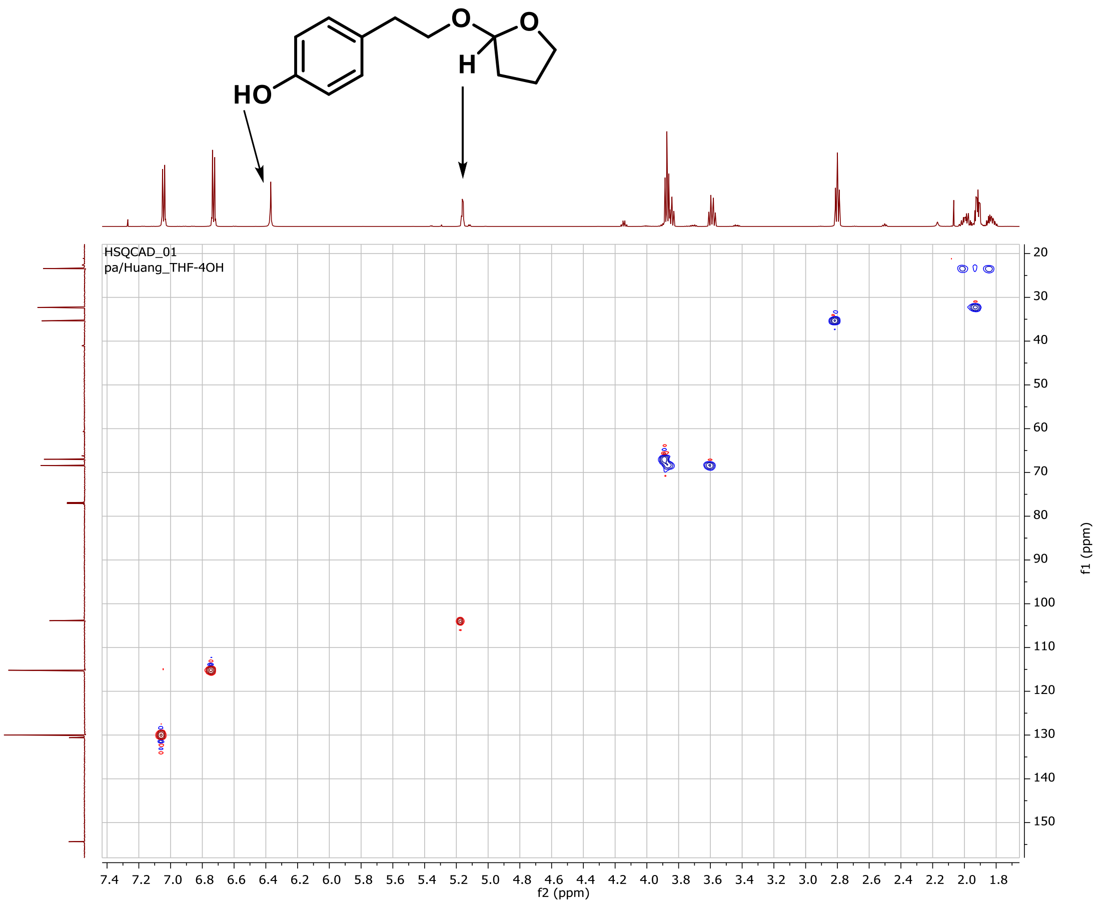
HO
O
O
H
HSQCAD_01
pa/Huang_THF-4OH
f2 (ppm)
f1 (ppm)

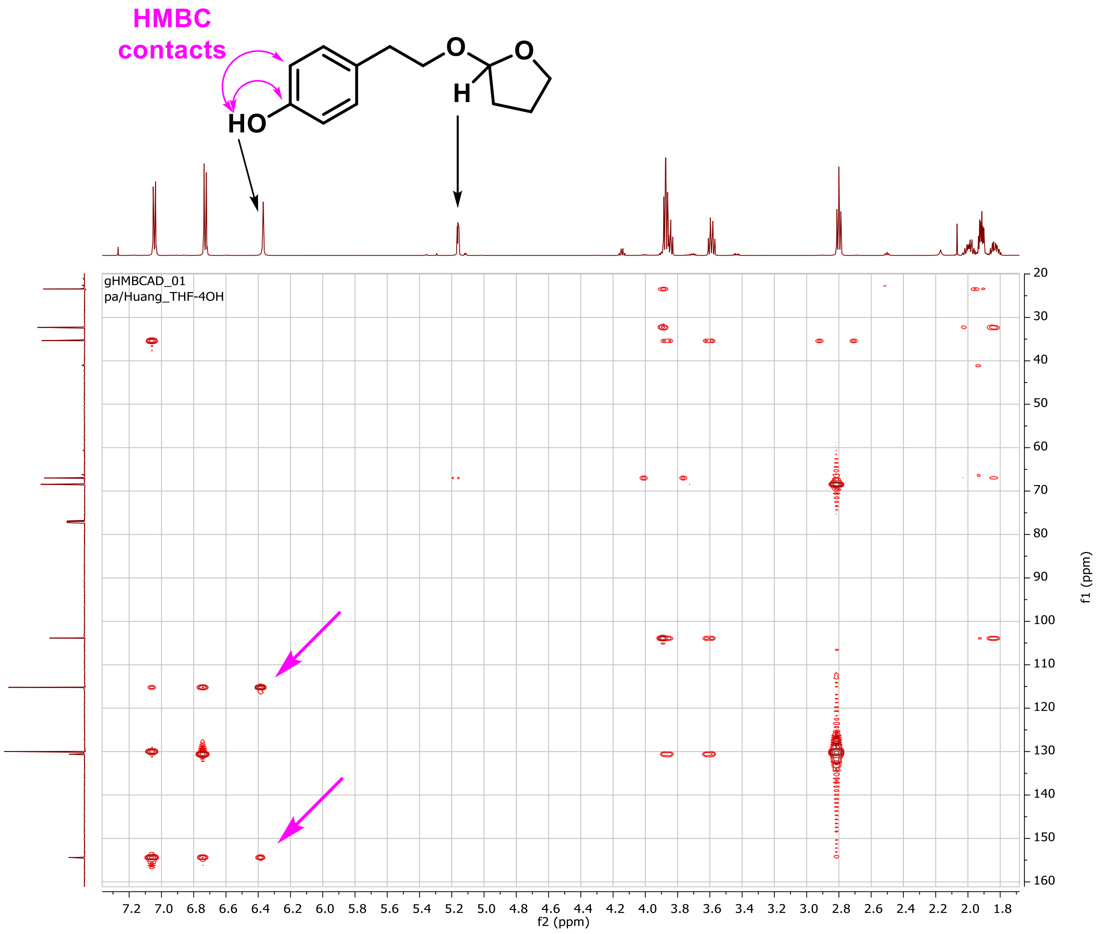
HMBC
contacts
HO
O
O
H
gHMBCAD_01
pa/Huang_THF-4OH
f2 (ppm)
f1 (ppm)

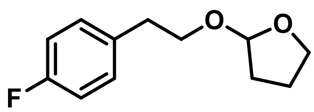

2ad

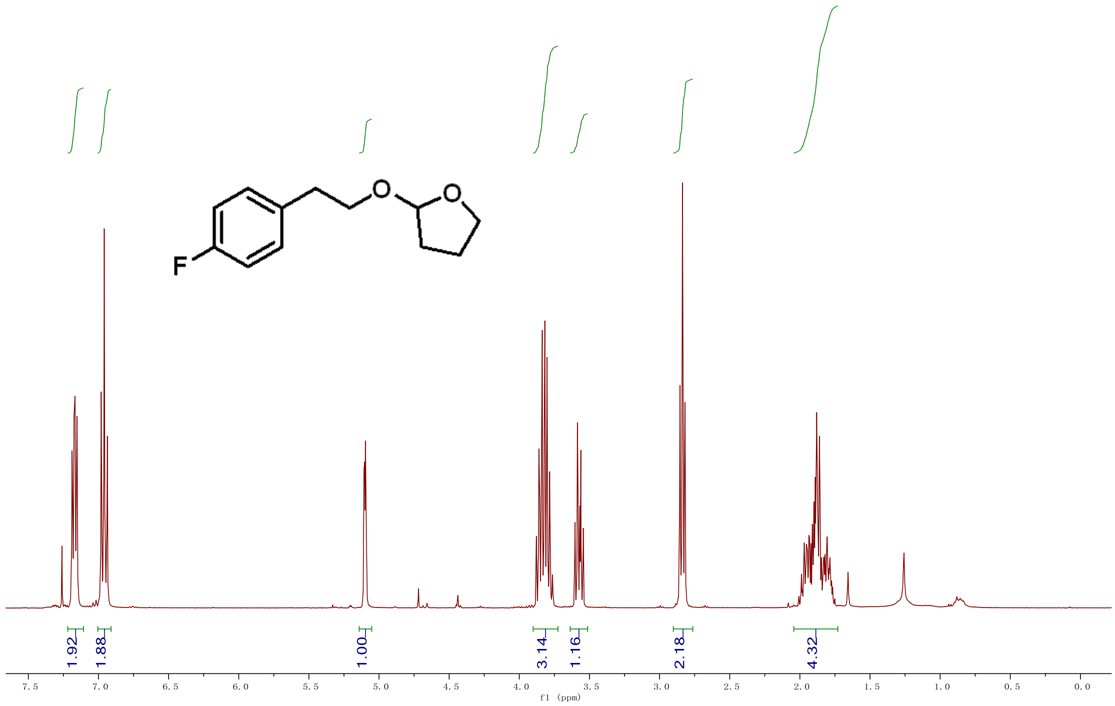

1.92
1.88
1.00
3.14
1.16
2.18
4.32
7.5
7.0
6.5
6.0
5.5
5.0
4.5
4.0
3.5
3.0
2.5
2.0
1.5
1.0
0.5
0.0
f1 (ppm)

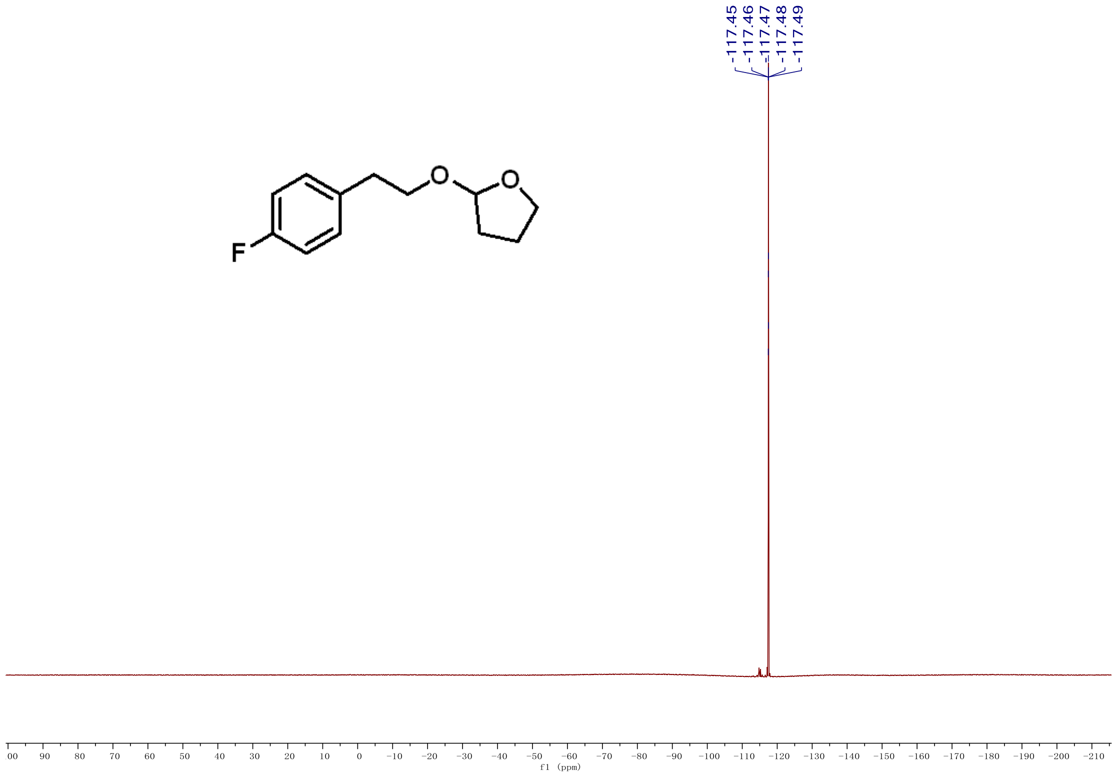

-117.45
-117.46
-117.47
-117.48
-117.49
f1 (ppm)

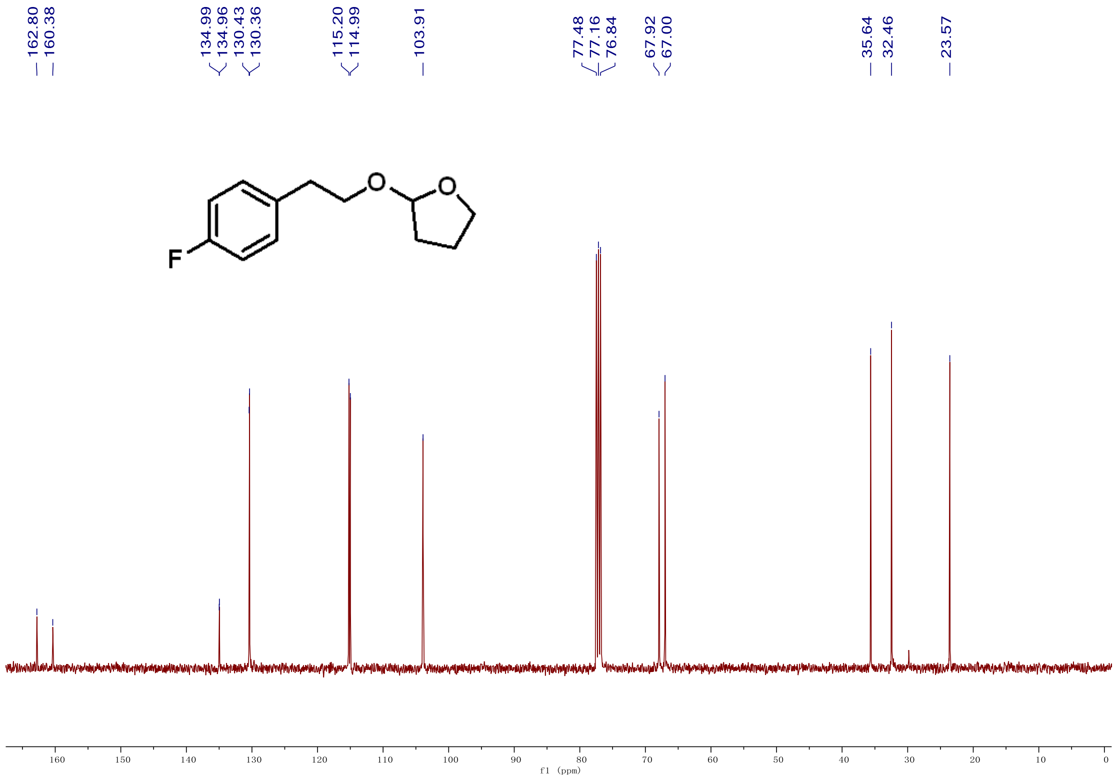
162.80
160.38
134.99
134.96
130.43
130.36
115.20
114.99
103.91
77.48
77.16
76.84
67.92
67.00
35.64
32.46
23.57
F
O
O
f1 (ppm)

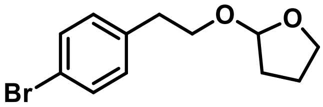
Br
O
O

2ae

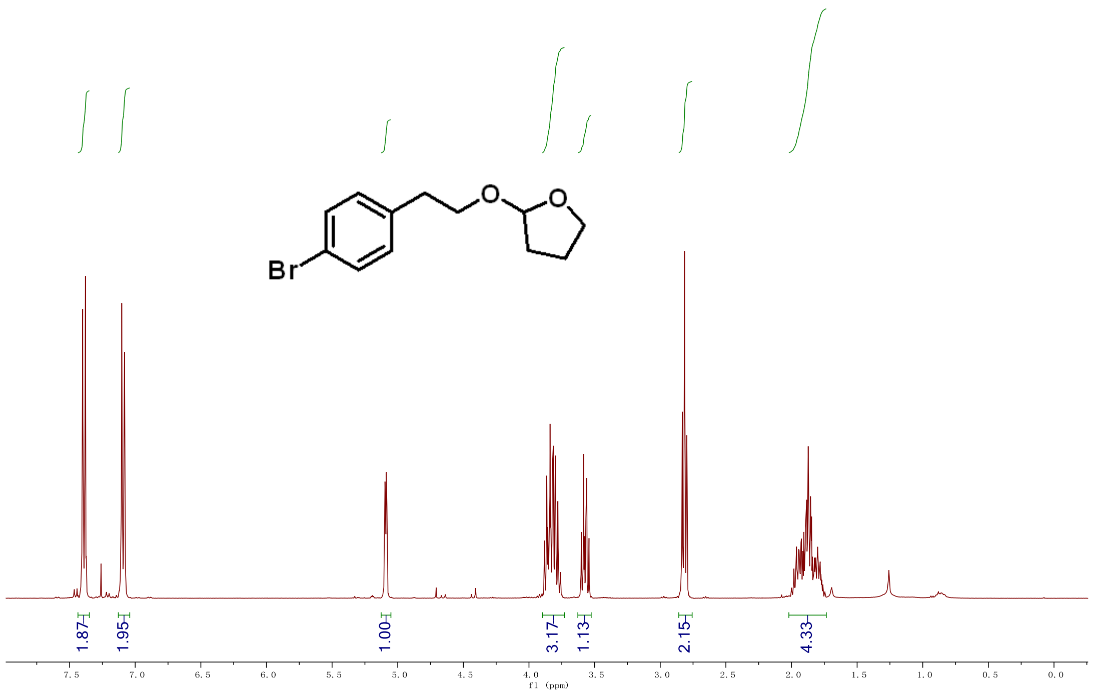
Br
O
O
1.87
1.95
1.00
3.17
1.13
2.15
4.33
f1 (ppm)

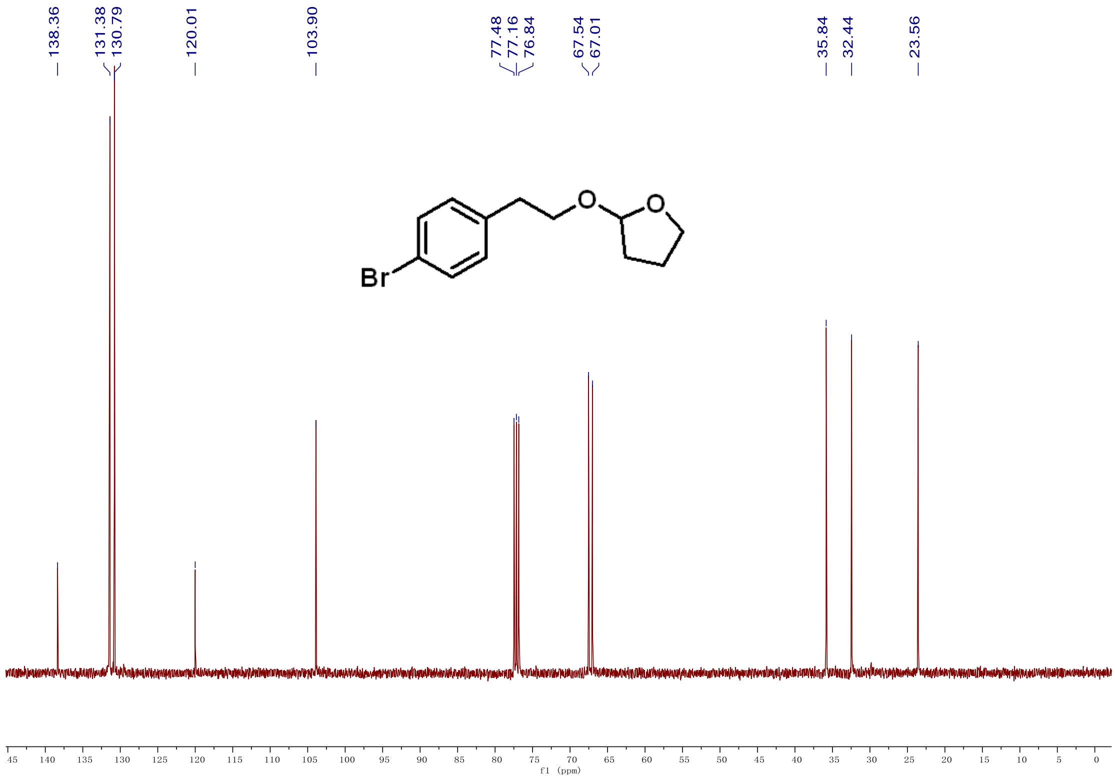

138.36
131.38
130.79
120.01
103.90
77.48
77.16
76.84
67.54
67.01
35.84
32.44
23.56
Br
O
f1 (ppm)

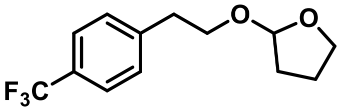

F_3C
O

2af

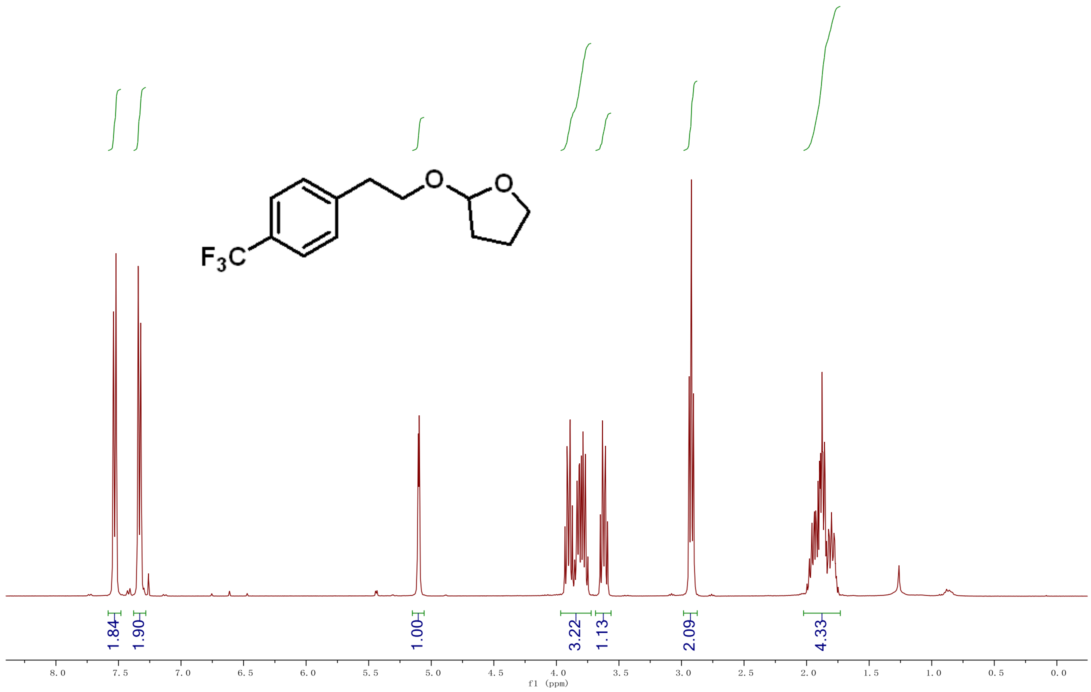

F_3C
O
1.84
1.90
1.00
3.22
1.13
2.09
4.33
f1 (ppm)

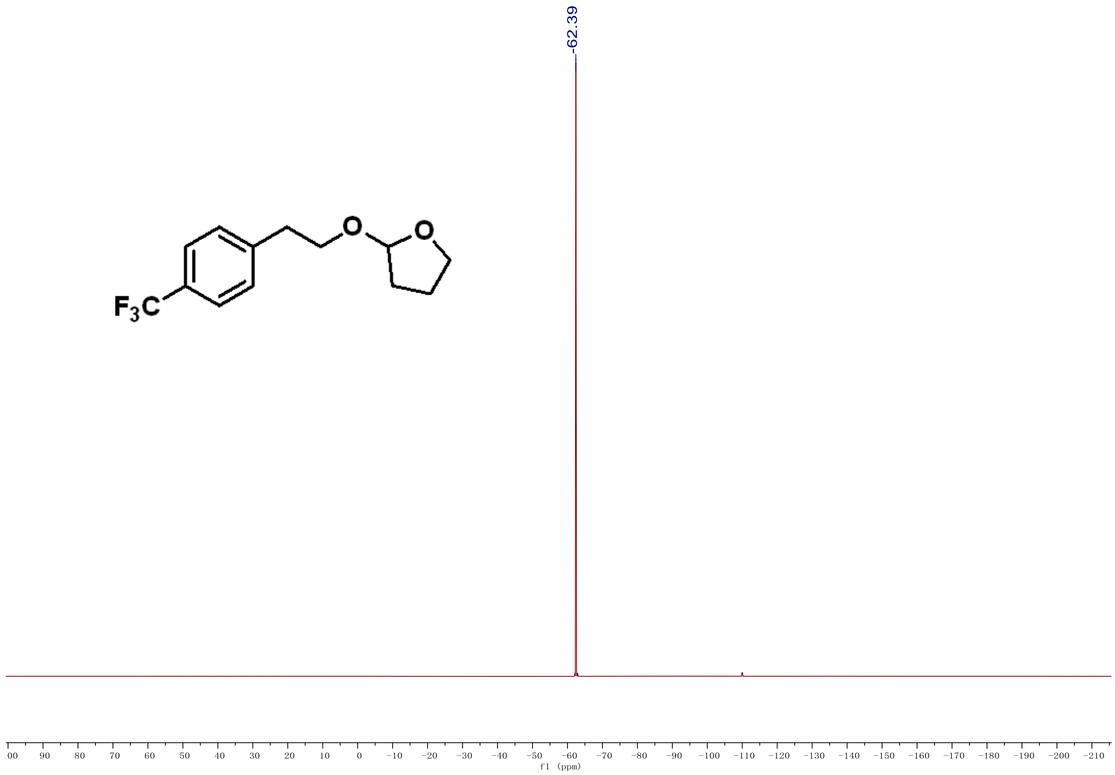
-62.39
F3C
f1 (ppm)

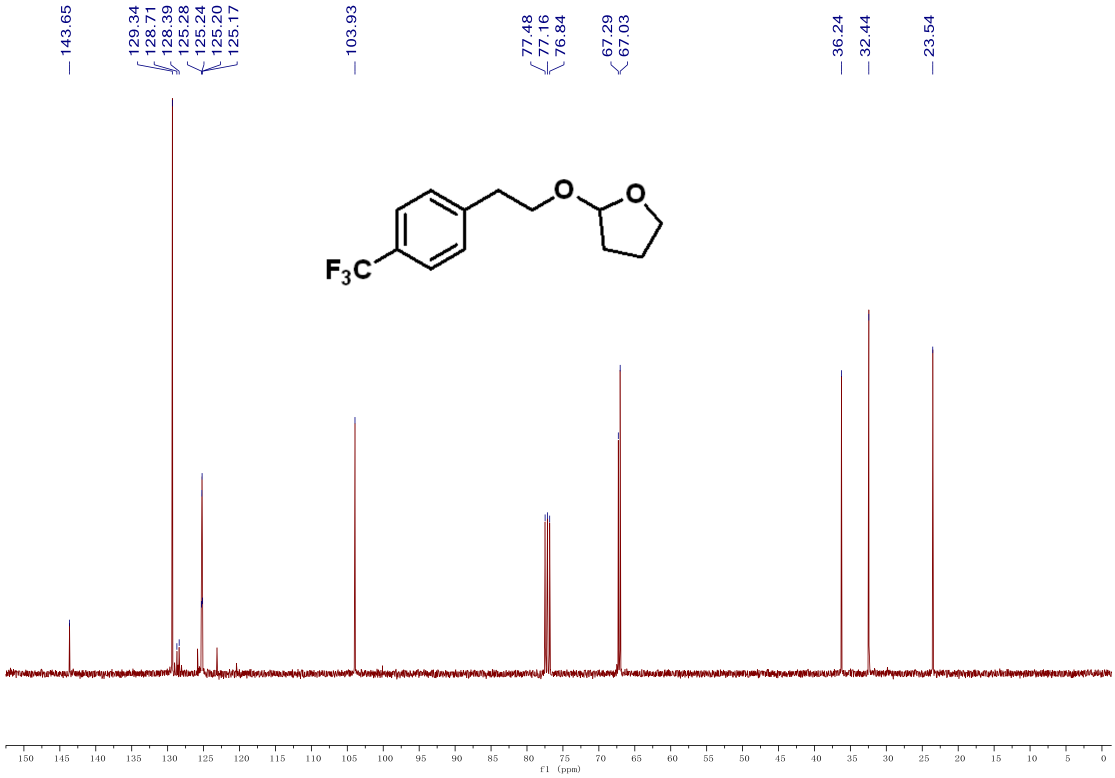
143.65
129.34
128.71
128.39
125.28
125.24
125.20
125.17
103.93
77.48
77.16
76.84
67.29
67.03
36.24
32.44
23.54
F3C
f1 (ppm)

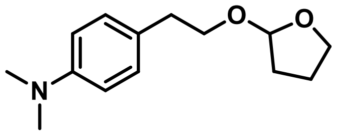

2ag

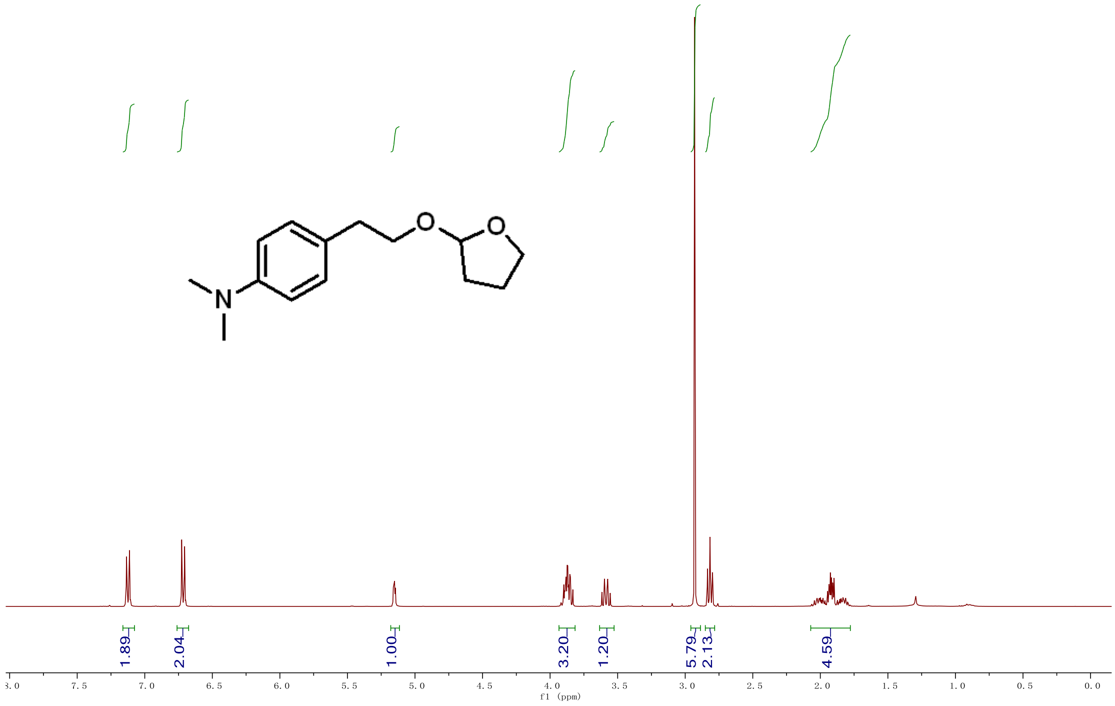

N
O
O
1.89
2.04
1.00
3.20
1.20
5.79
2.13
4.59
8.0 7.5 7.0 6.5 6.0 5.5 5.0 4.5 4.0 3.5 3.0 2.5 2.0 1.5 1.0 0.5 0.0
f1 (ppm)

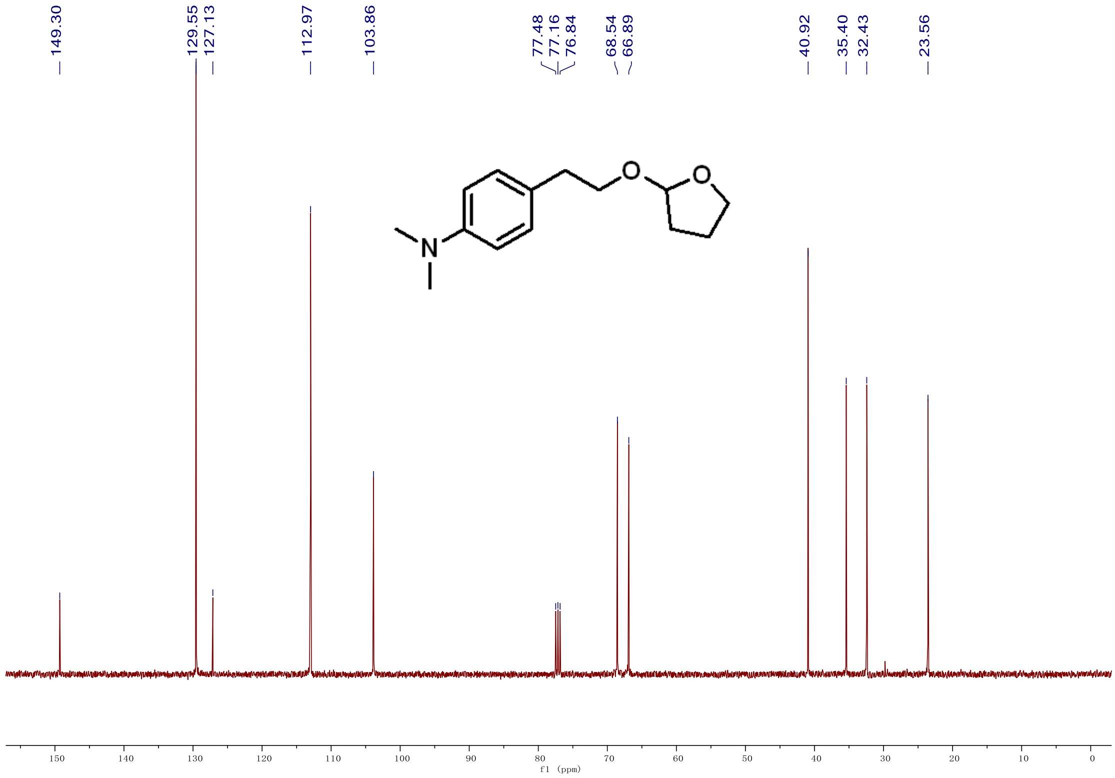

149.30
129.55
127.13
112.97
103.86
77.48
77.16
76.84
68.54
66.89
40.92
35.40
32.43
23.56
N
O
O
150 140 130 120 110 100 90 80 70 60 50 40 30 20 10 0
f1 (ppm)

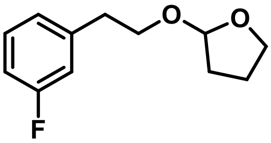

O
O
F

2ah

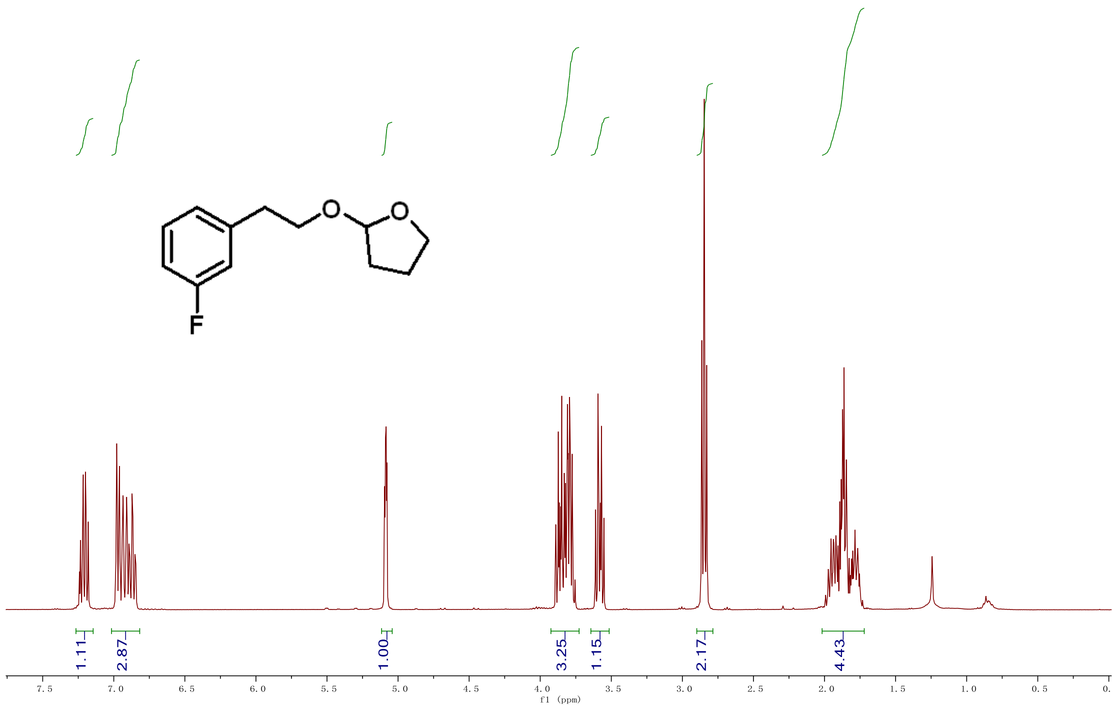
1.11
2.87
1.00
3.25
1.15
2.17
4.43
F
O
O
7.5
7.0
6.5
6.0
5.5
5.0
4.5
4.0
3.5
3.0
2.5
2.0
1.5
1.0
0.5
f1 (ppm)

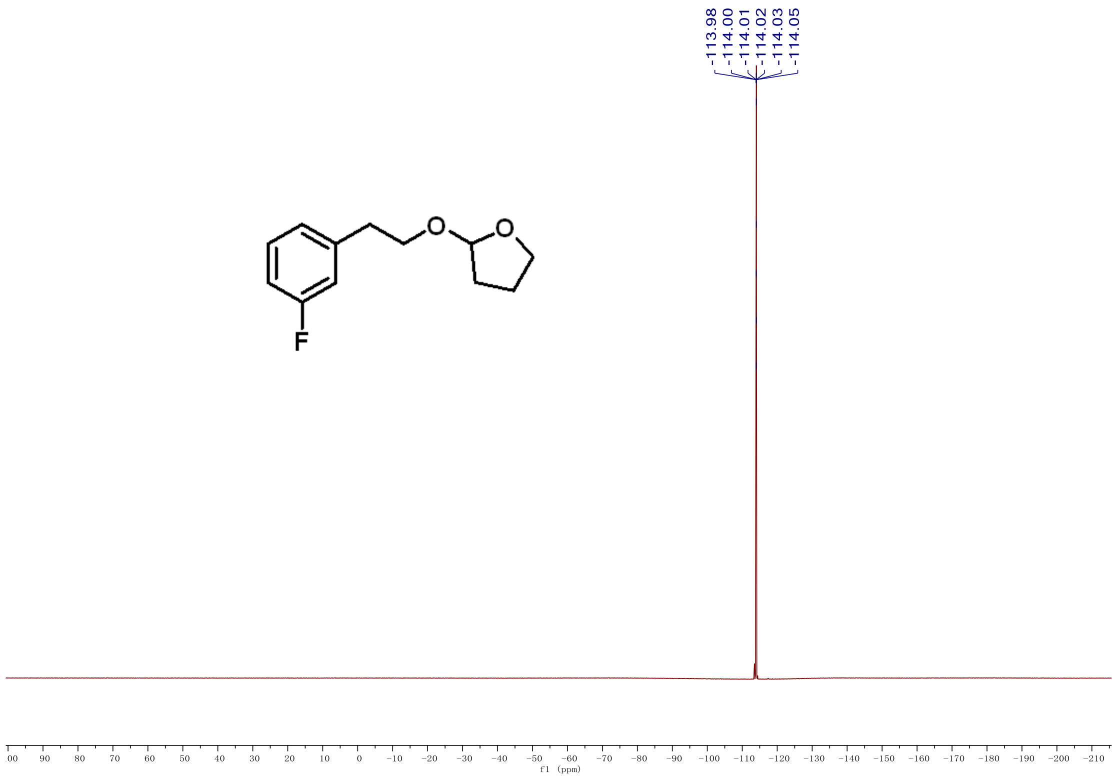
-113.98
-114.00
-114.01
-114.02
-114.03
-114.05
F
O
O
f1 (ppm)

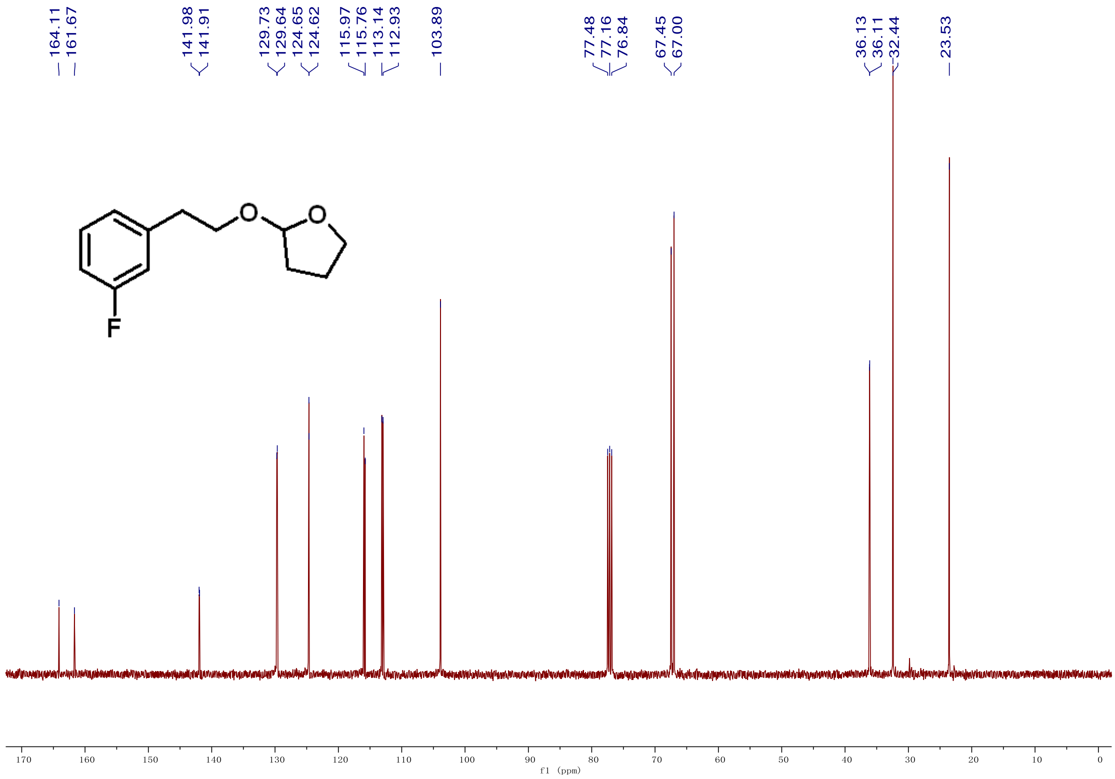
164.11
161.67
141.98
141.91
129.73
129.64
124.65
124.62
115.97
115.76
113.14
112.93
103.89
77.48
77.16
76.84
67.45
67.00
36.13
36.11
32.44
23.53
F
O
f1 (ppm)

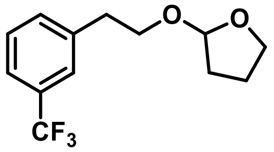
O
CF3

2ai

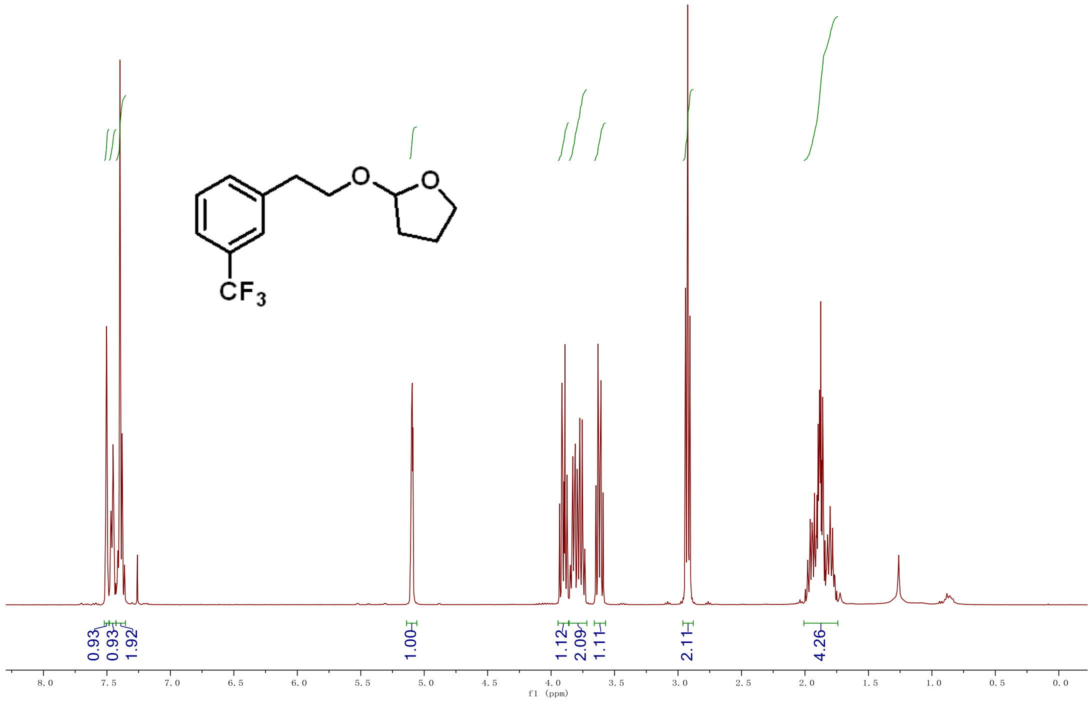
O
CF3
0.93
0.93
1.92
1.00
1.12
2.09
1.11
2.11
4.26
f1 (ppm)

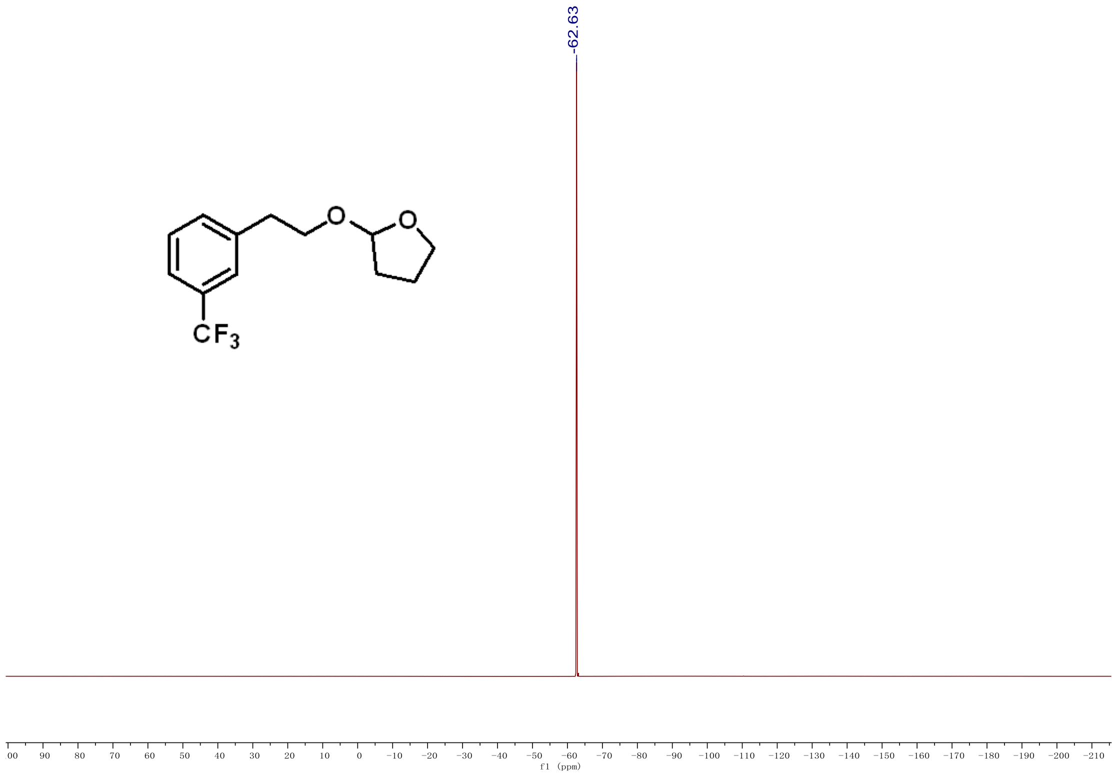
-62.63
CF3
O
O
f1 (ppm)

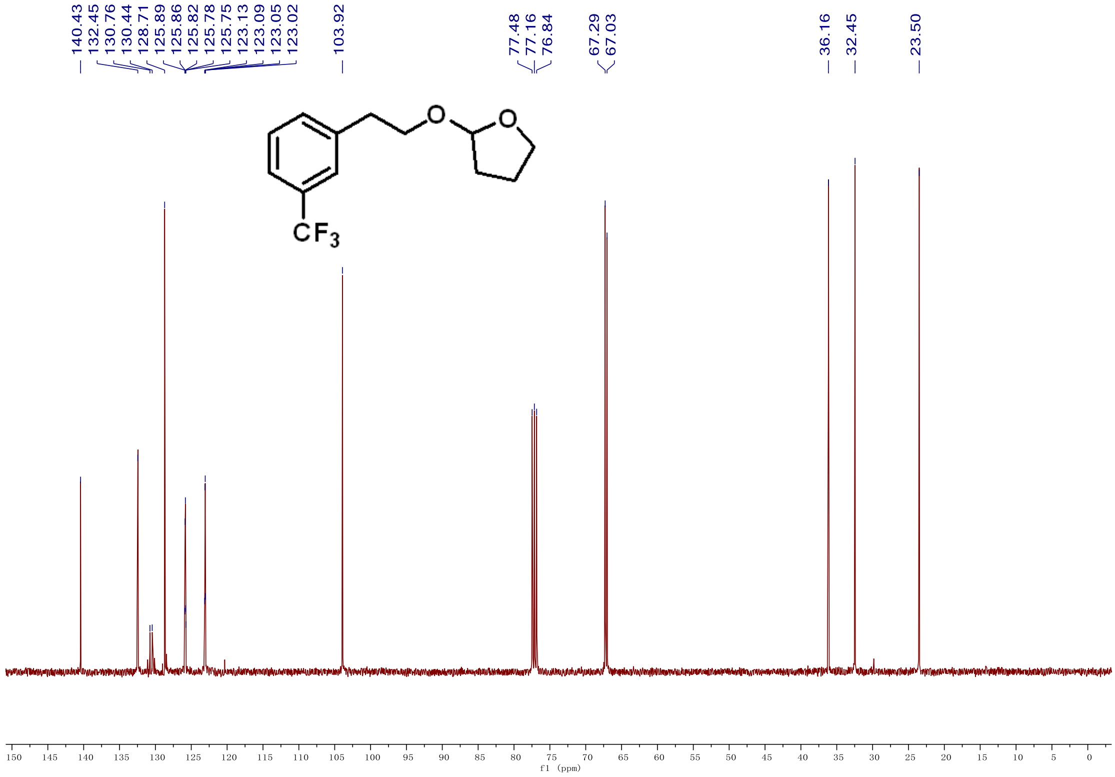
140.43
132.45
130.76
130.44
128.71
125.89
125.86
125.82
125.78
125.75
123.13
123.09
123.05
123.02
103.92
77.48
77.16
76.84
67.29
67.03
36.16
32.45
23.50
CF3
O
O
f1 (ppm)

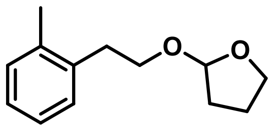
O
O
2aj

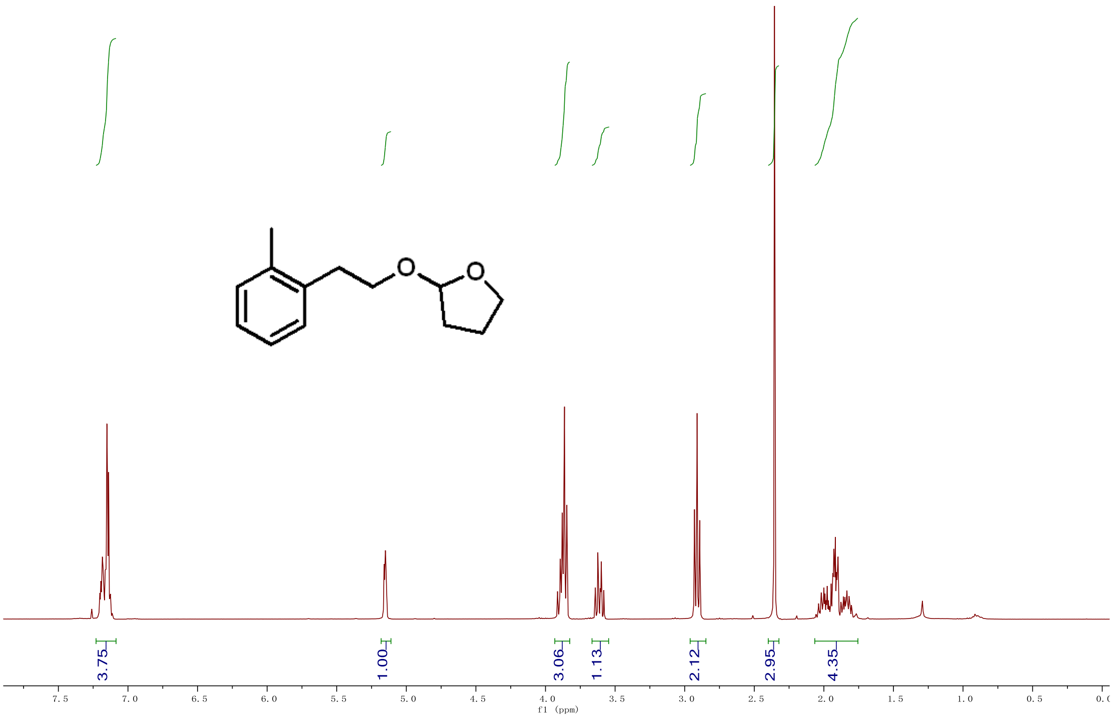
3.75
1.00
3.06
1.13
2.12
2.95
4.35
f1 (ppm)

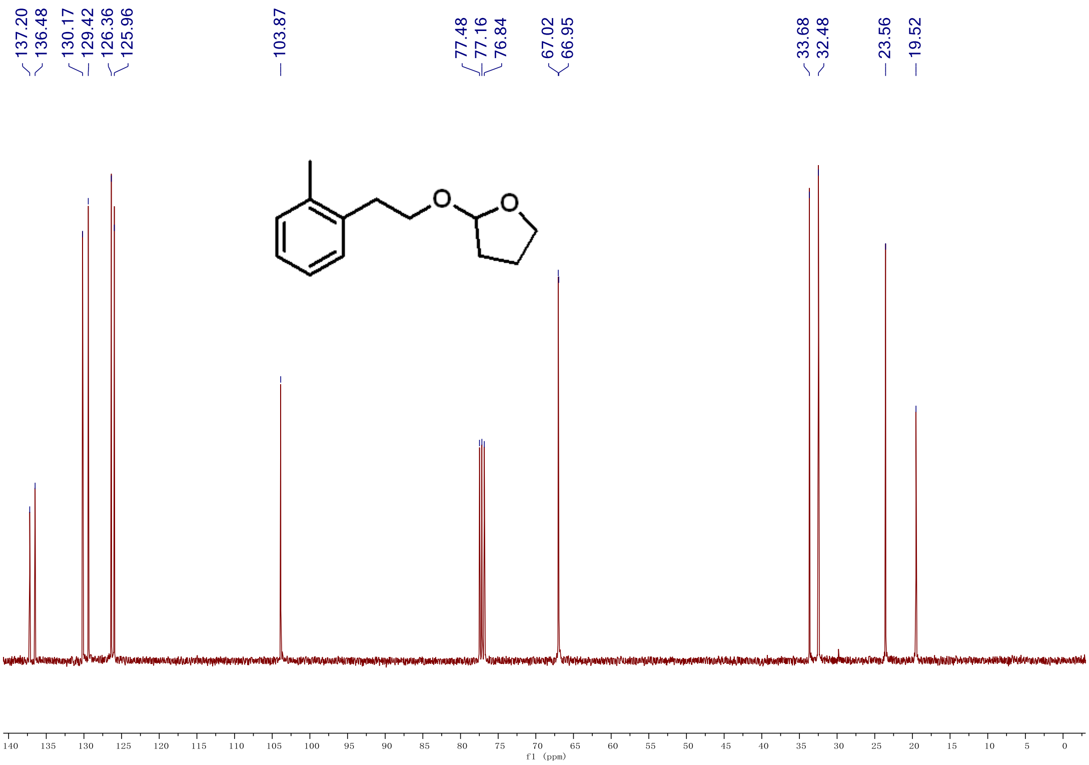
137.20
136.48
130.17
129.42
126.36
125.96
103.87
77.48
77.16
76.84
67.02
66.95
33.68
32.48
23.56
19.52
f1 (ppm)

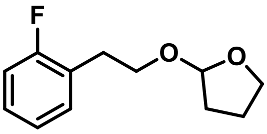
2ak

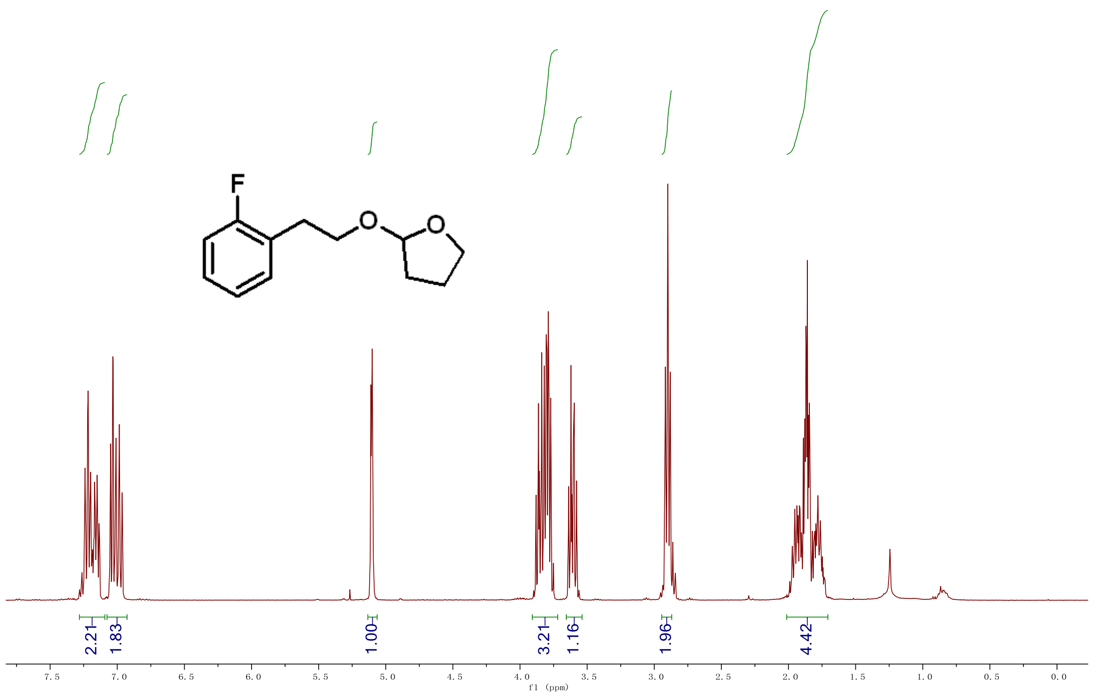
F
O
O
2.21
1.83
1.00
3.21
1.16
1.96
4.42
7.5
7.0
6.5
6.0
5.5
5.0
4.5
4.0
3.5
3.0
2.5
2.0
1.5
1.0
0.5
0.0
f1 (ppm)

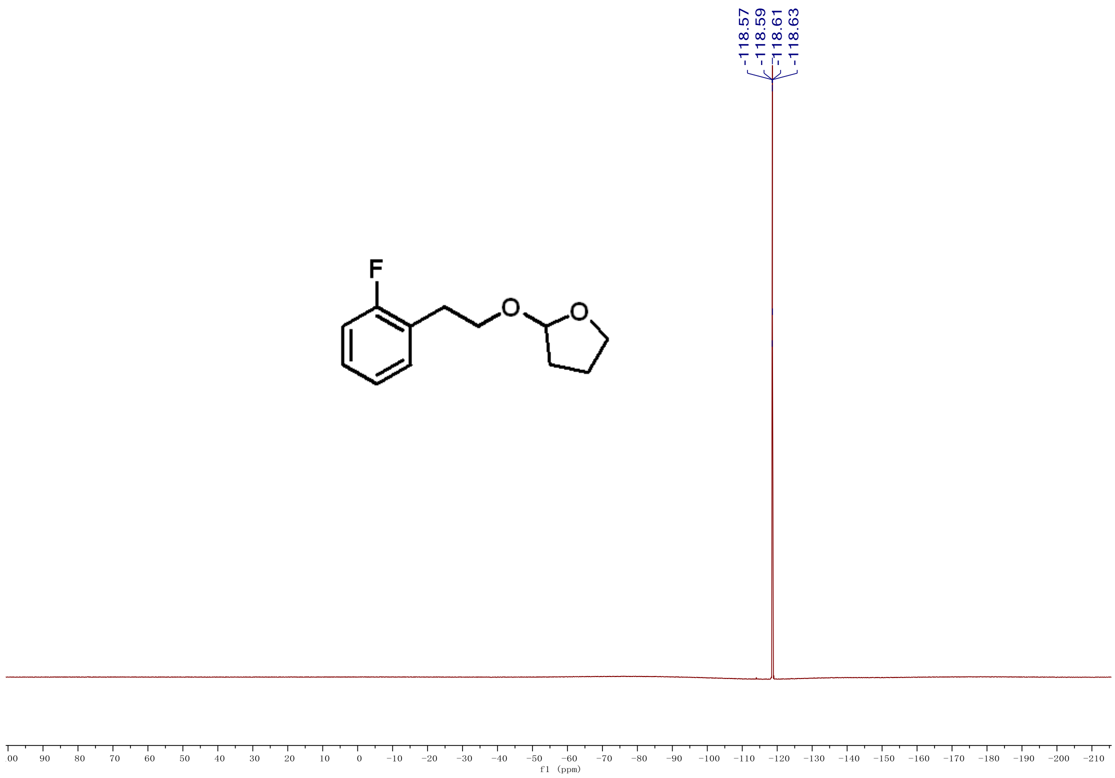
-118.57
-118.59
-118.61
-118.63
F
O
O
f1 (ppm)

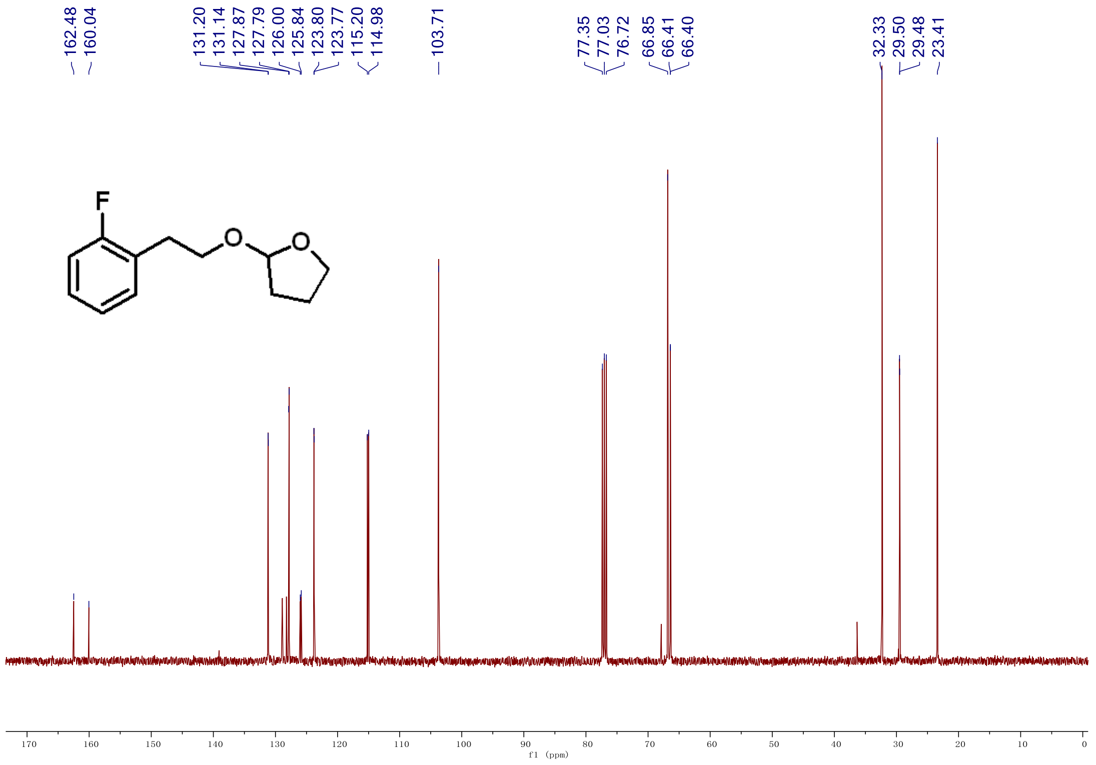
162.48
160.04
131.20
131.14
127.87
127.79
126.00
125.84
123.80
123.77
115.20
114.98
103.71
77.35
77.03
76.72
66.85
66.41
66.40
32.33
29.50
29.48
23.41
F
O
O
f1 (ppm)

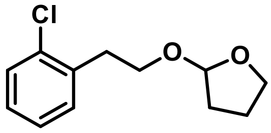

2al

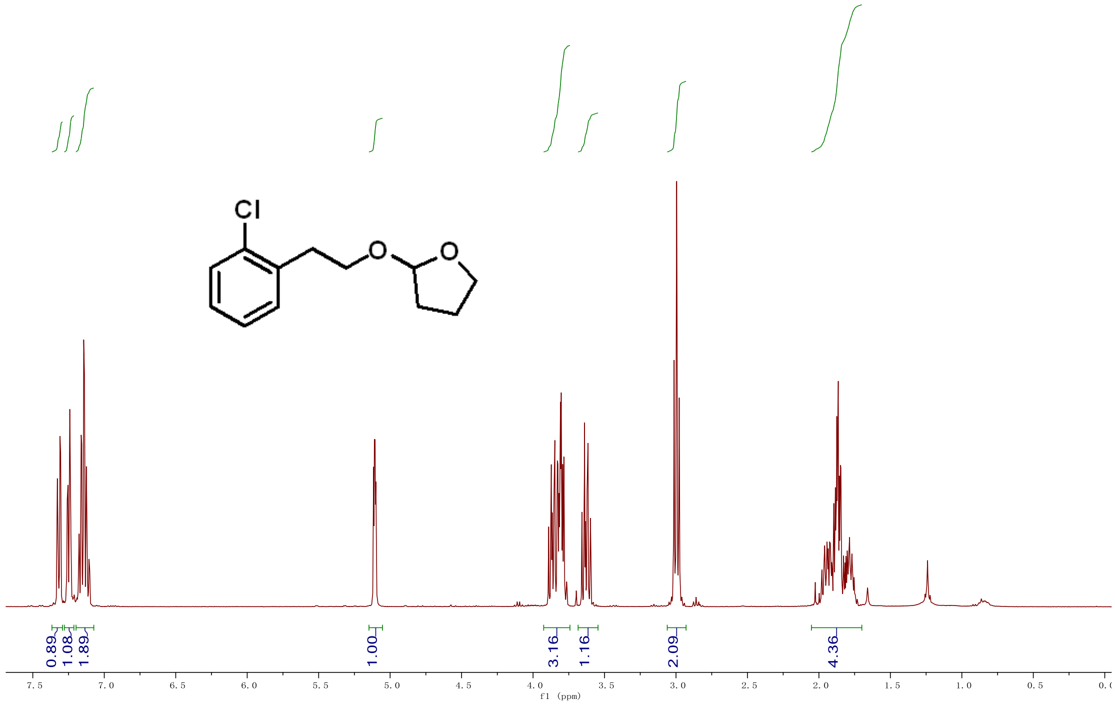
Cl
O
O
0.89
1.08
1.89
1.00
3.16
1.16
2.09
4.36
f1 (ppm)

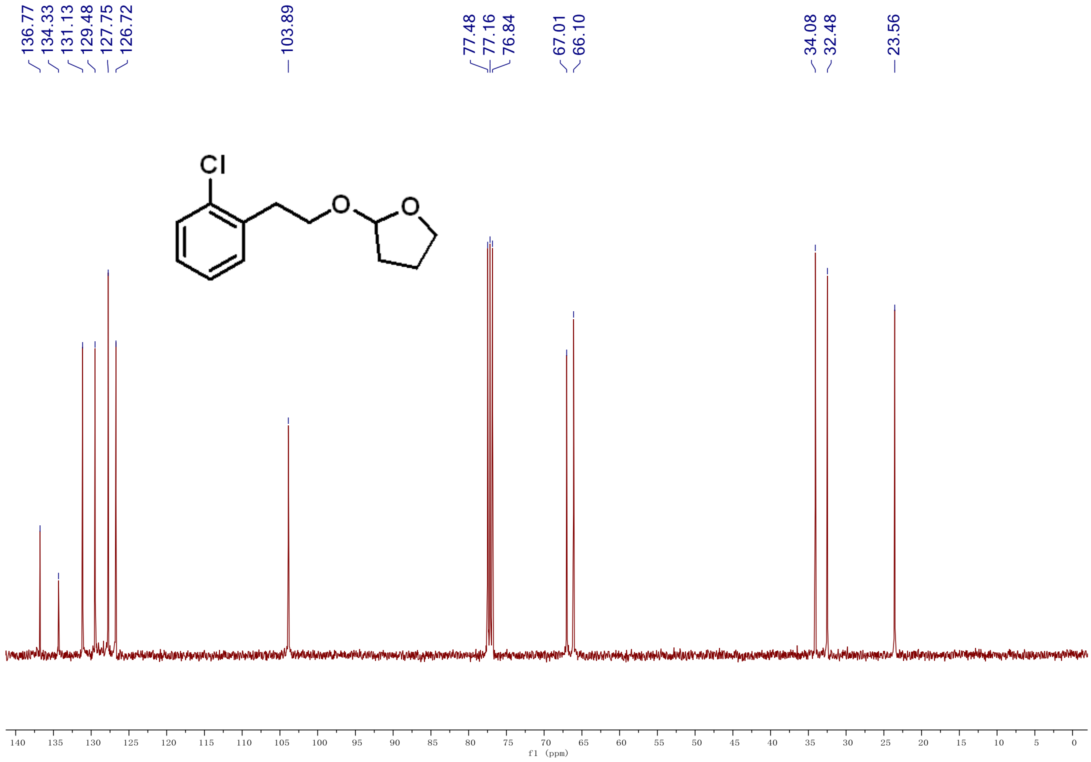
136.77
134.33
131.13
129.48
127.75
126.72
103.89
77.48
77.16
76.84
67.01
66.10
34.08
32.48
23.56
Cl
O
O
140 135 130 125 120 115 110 105 100 95 90 85 80 75 70 65 60 55 50 45 40 35 30 25 20 15 10 5 0
f1 (ppm)

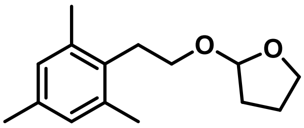
O
O

2am

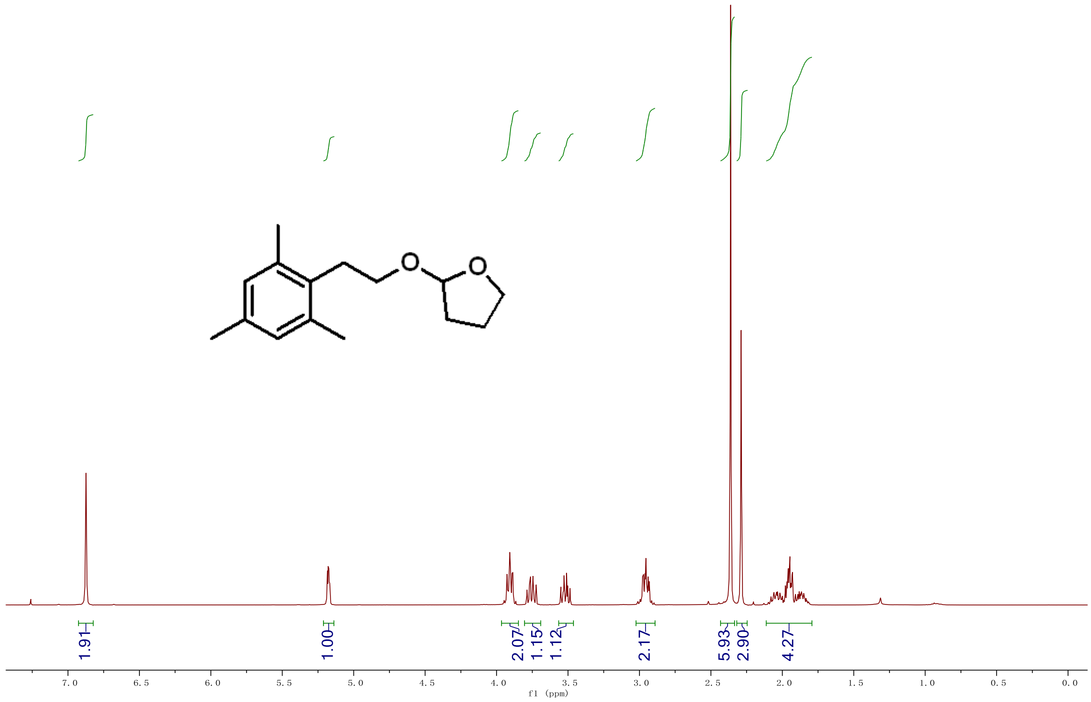
O
O
1.91
1.00
2.07
1.15
1.12
2.17
5.93
2.90
4.27
7.0 6.5 6.0 5.5 5.0 4.5 4.0 3.5 3.0 2.5 2.0 1.5 1.0 0.5 0.0
f1 (ppm)

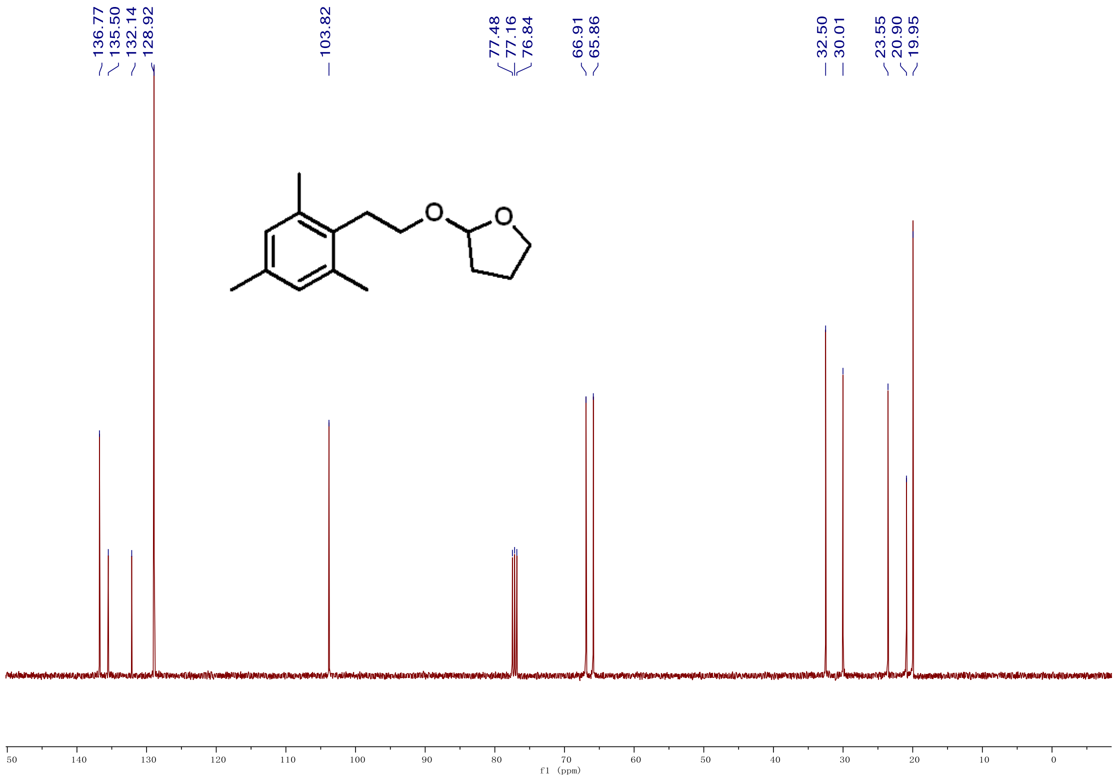
136.77
135.50
132.14
128.92
103.82
77.48
77.16
76.84
66.91
65.86
32.50
30.01
23.55
20.90
19.95
f1 (ppm)

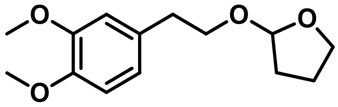

2an

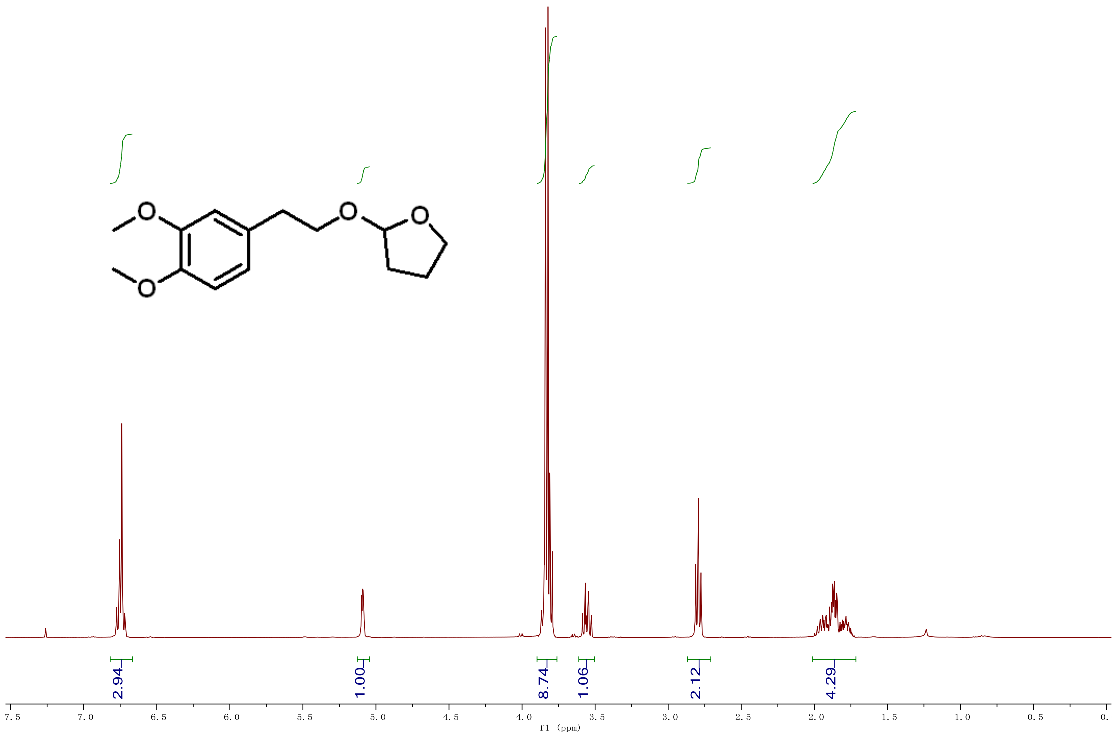
2.94
1.00
8.74
1.06
2.12
4.29
f1 (ppm)

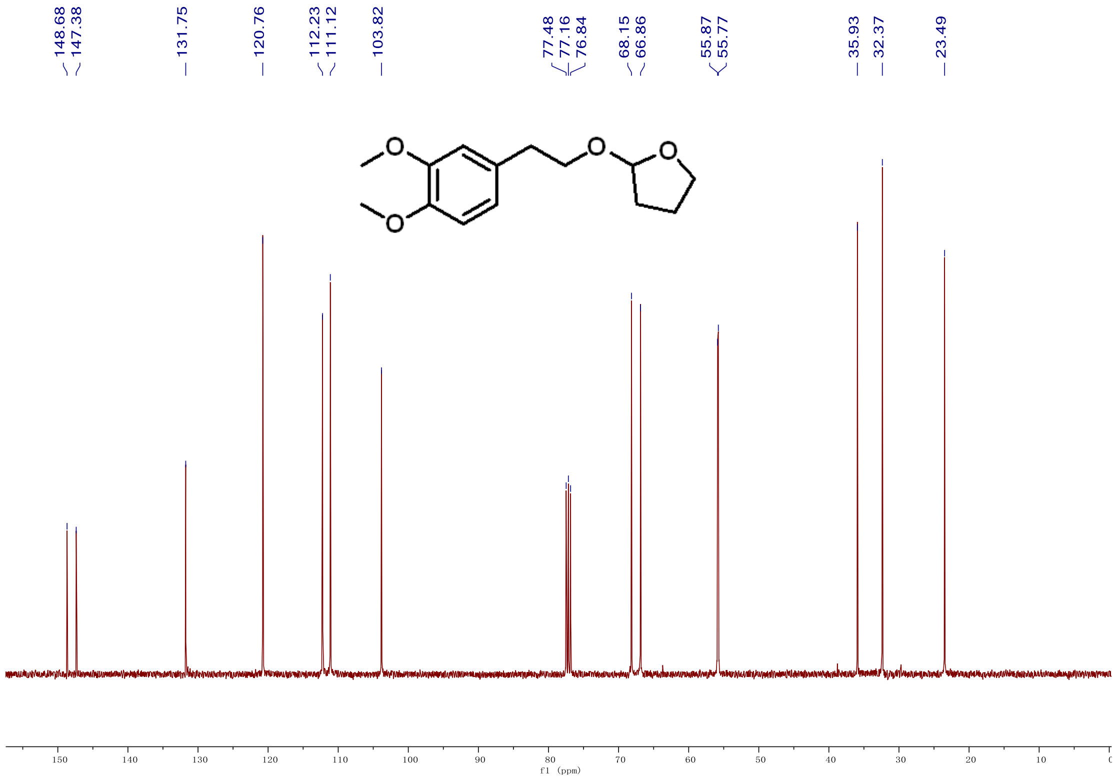
148.68
147.38
131.75
120.76
112.23
111.12
103.82
77.48
77.16
76.84
68.15
66.86
55.87
55.77
35.93
32.37
23.49
f1 (ppm)

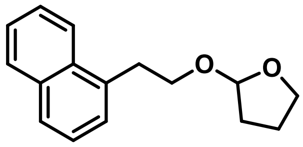

2ao

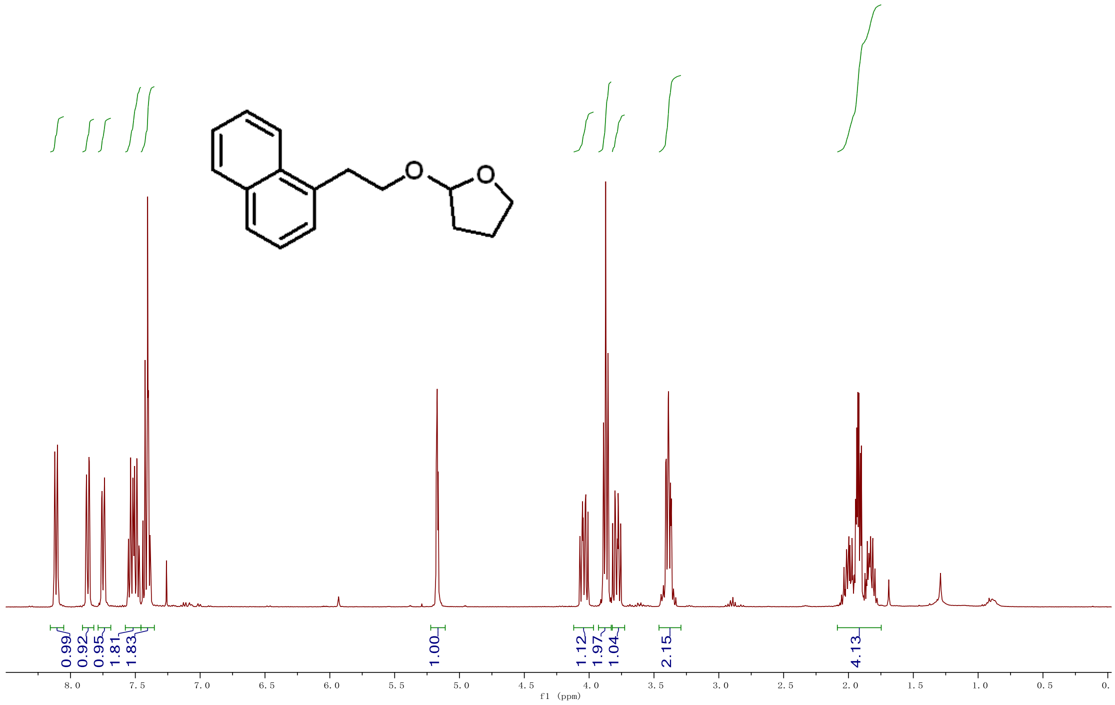
0.99
0.92
0.95
1.81
1.83
1.00
1.12
1.97
1.04
2.15
4.13
f1 (ppm)

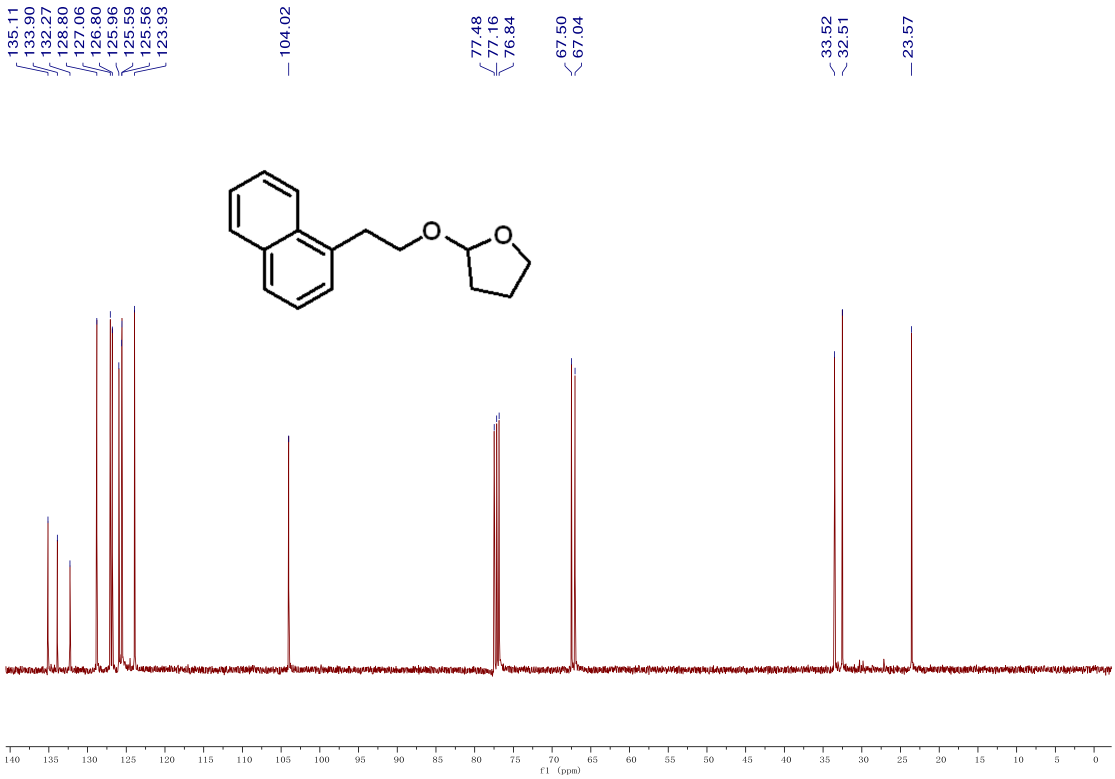
135.11
133.90
132.27
128.80
127.06
126.80
125.96
125.59
125.56
123.93
104.02
77.48
77.16
76.84
67.50
67.04
33.52
32.51
23.57
140 135 130 125 120 115 110 105 100 95 90 85 80 75 70 65 60 55 50 45 40 35 30 25 20 15 10 5 0
f1 (ppm)

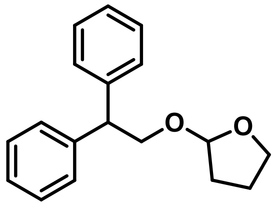

2ap

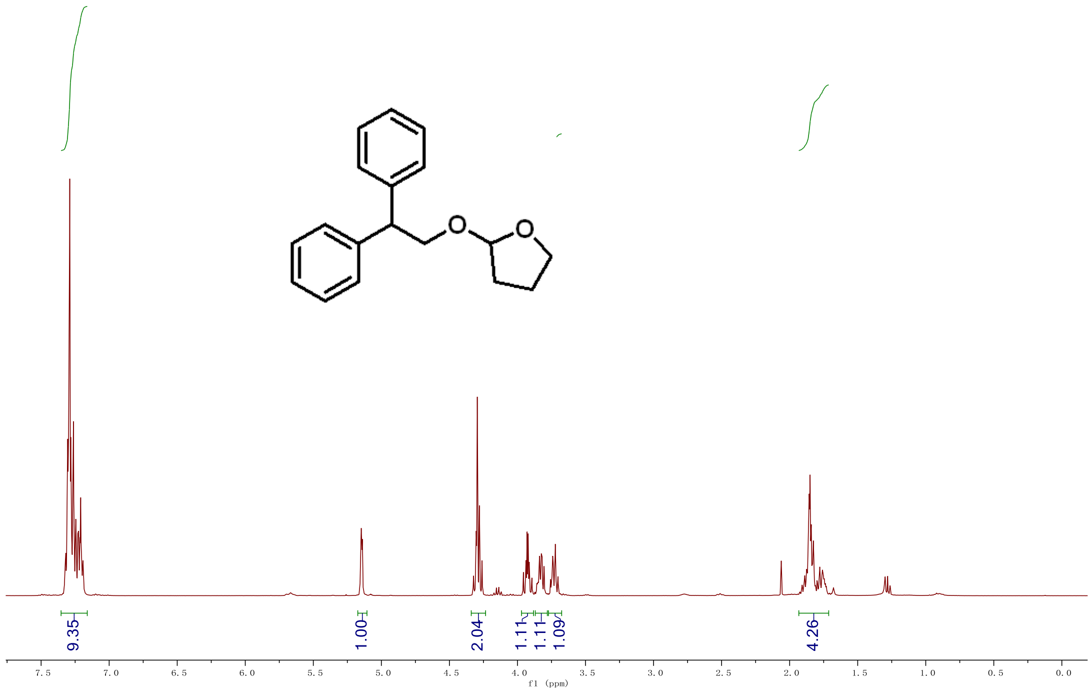
9.35
1.00
2.04
1.11
1.11
1.09
4.26
7.5 7.0 6.5 6.0 5.5 5.0 4.5 4.0 3.5 3.0 2.5 2.0 1.5 1.0 0.5 0.0
f1 (ppm)

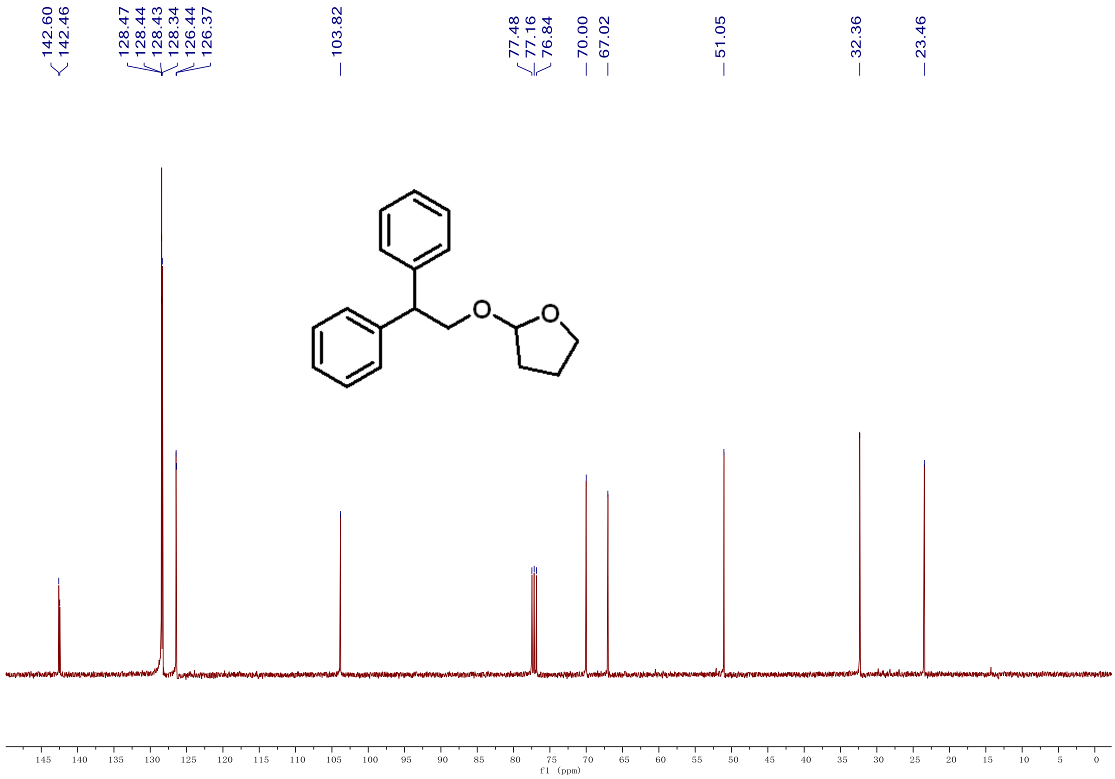
142.60
142.46
128.47
128.44
128.43
128.34
126.44
126.37
103.82
77.48
77.16
76.84
70.00
67.02
51.05
32.36
23.46
O
O
f1 (ppm)

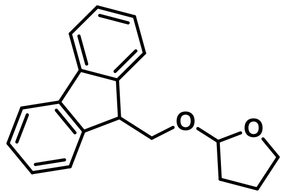
O
O

2aq

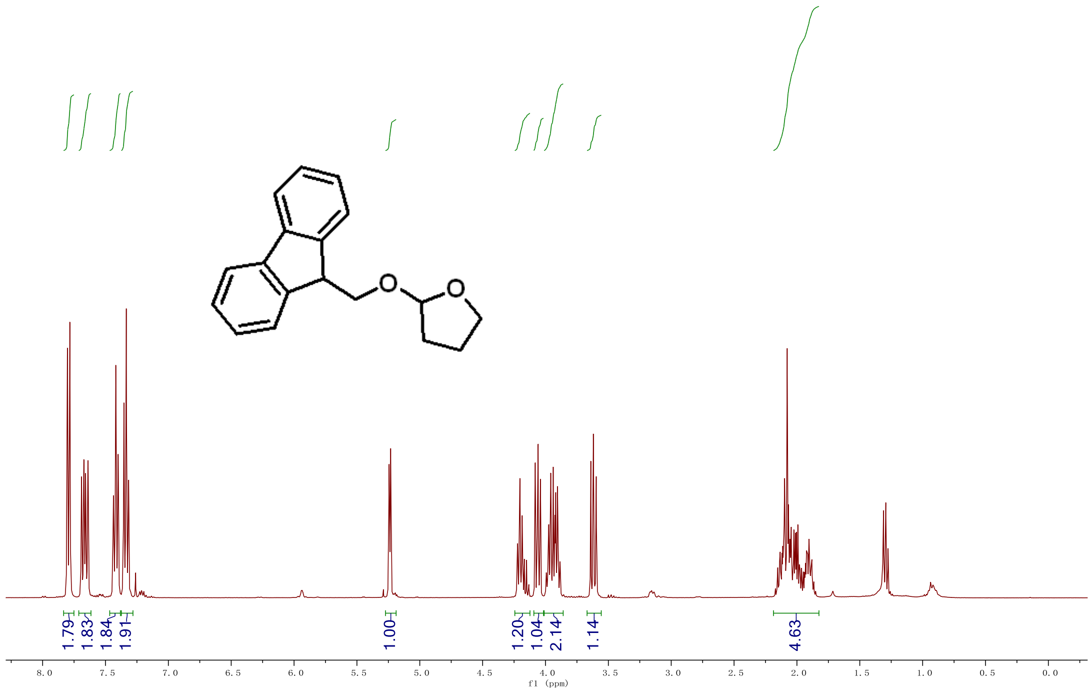
O
O
1.79
1.83
1.84
1.91
1.00
1.20
1.04
2.14
1.14
4.63
f1 (ppm)

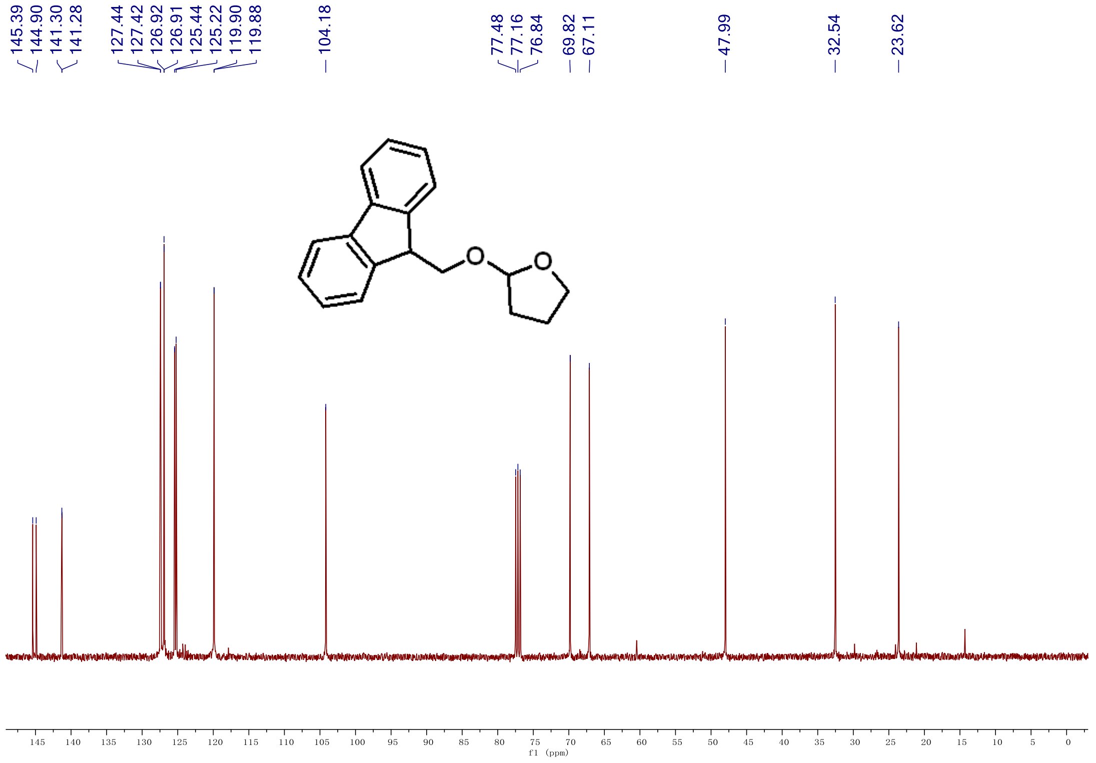
145.39
144.90
141.30
141.28
127.44
127.42
126.92
126.91
125.44
125.22
119.90
119.88
104.18
77.48
77.16
76.84
69.82
67.11
47.99
32.54
23.62
f1 (ppm)

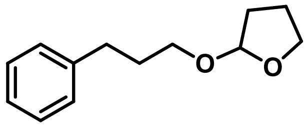

2ar

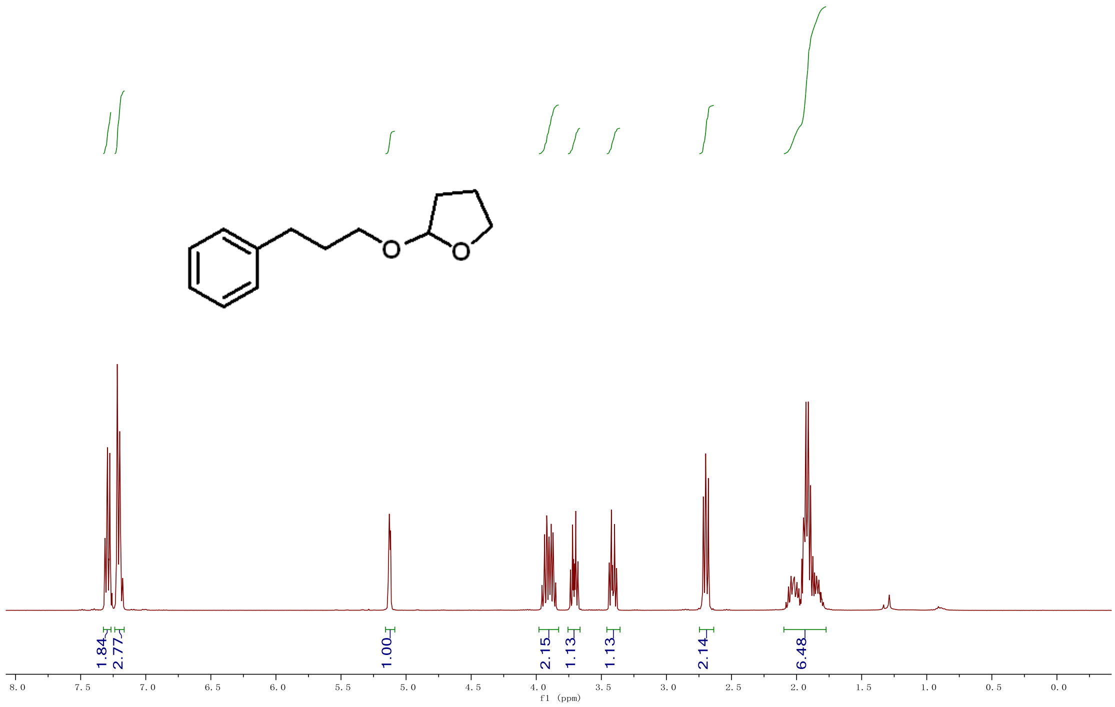
1.84
2.77
1.00
2.15
1.13
1.13
2.14
6.48
f1 (ppm)

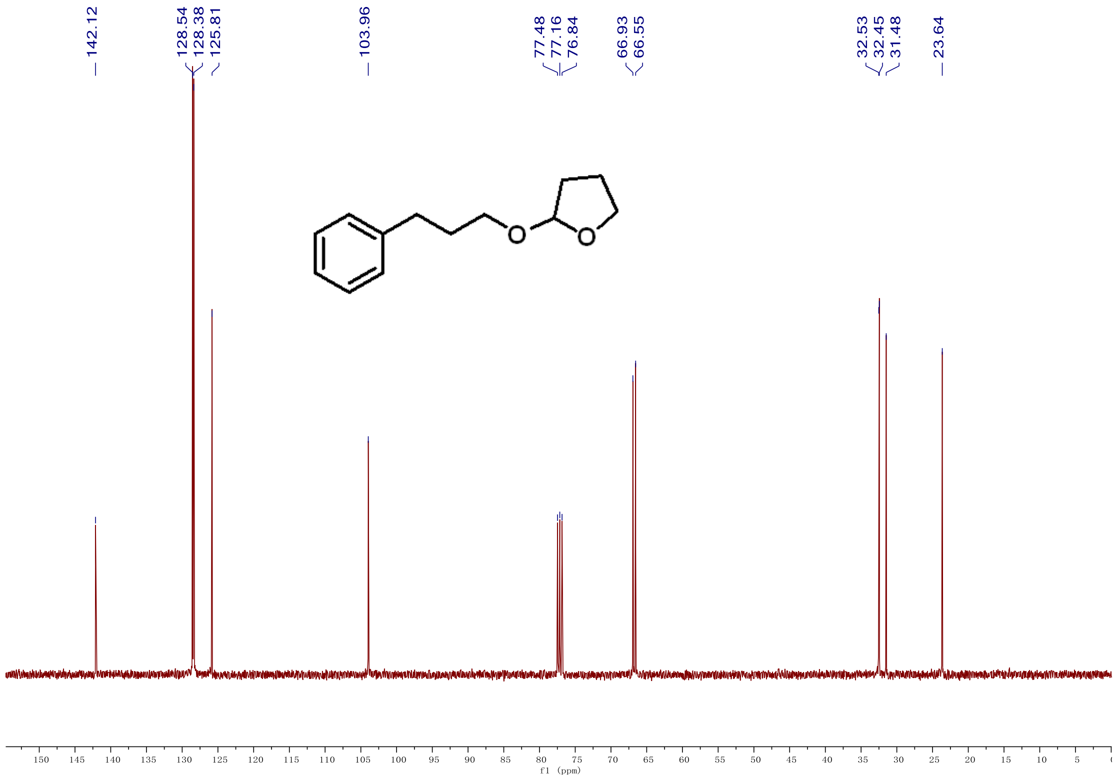
142.12
128.54
128.38
125.81
103.96
77.48
77.16
76.84
66.93
66.55
32.53
32.45
31.48
23.64
f1 (ppm)

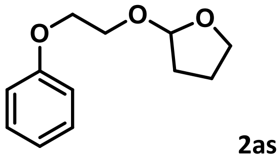

2as

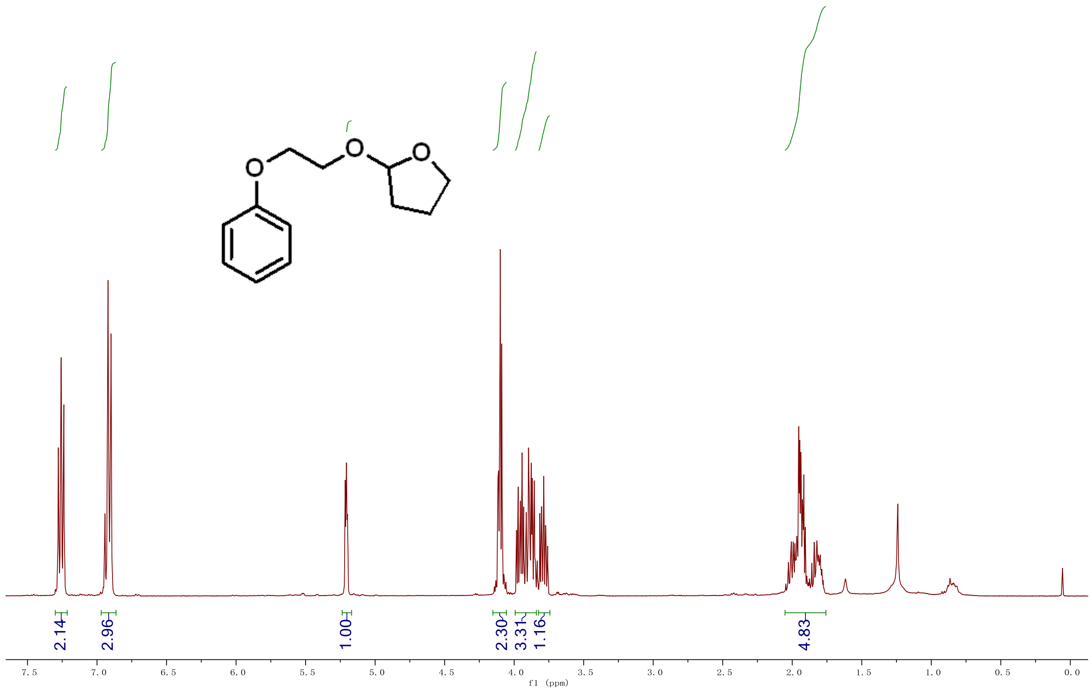
2.14
2.96
1.00
2.30
3.31
1.16
4.83
f1 (ppm)

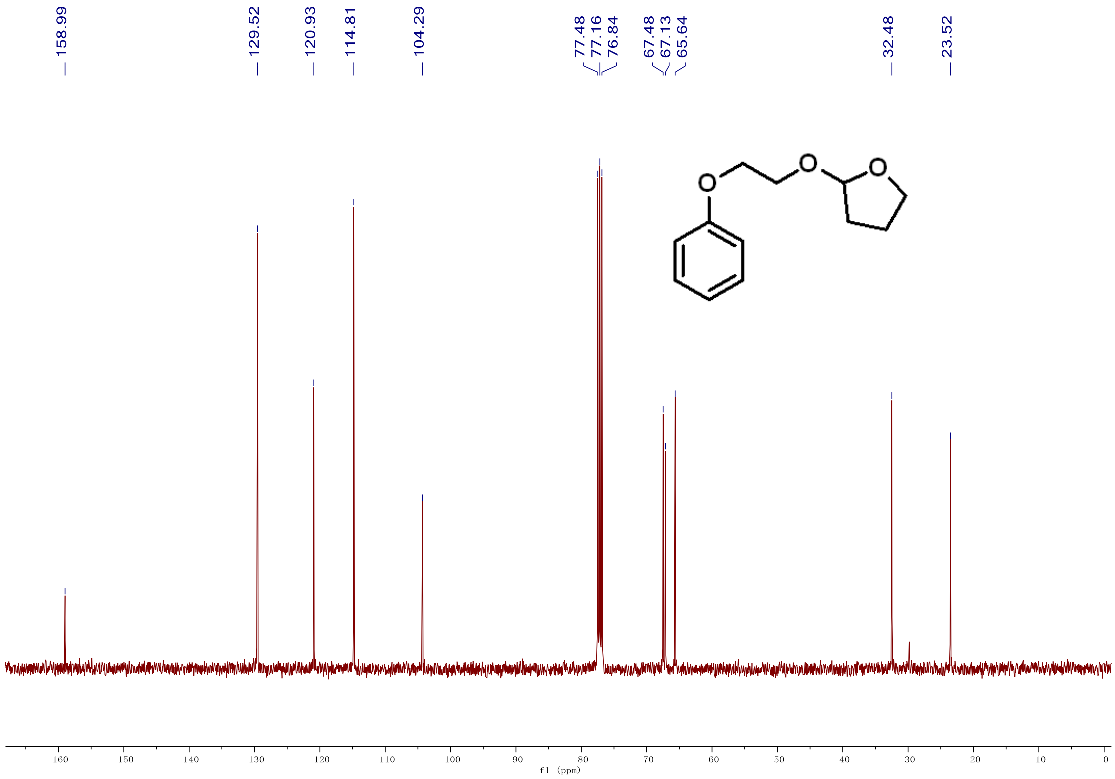
158.99
129.52
120.93
114.81
104.29
77.48
77.16
76.84
67.48
67.13
65.64
32.48
23.52
O
O
O
160 150 140 130 120 110 100 90 80 70 60 50 40 30 20 10 0
f1 (ppm)

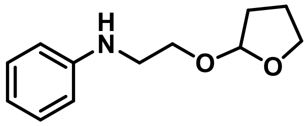
H
N
O
O

2at

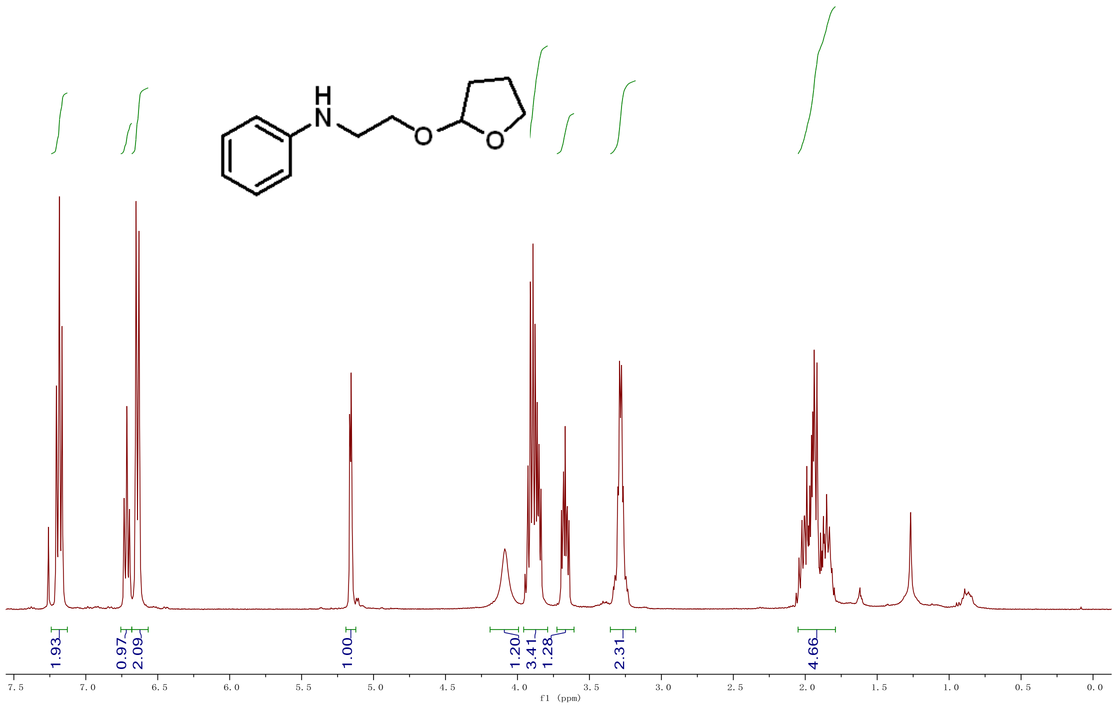
H
N
O
O
1.93
0.97
2.09
1.00
1.20
3.41
1.28
2.31
4.66
7.5 7.0 6.5 6.0 5.5 5.0 4.5 4.0 3.5 3.0 2.5 2.0 1.5 1.0 0.5 0.0
f1 (ppm)

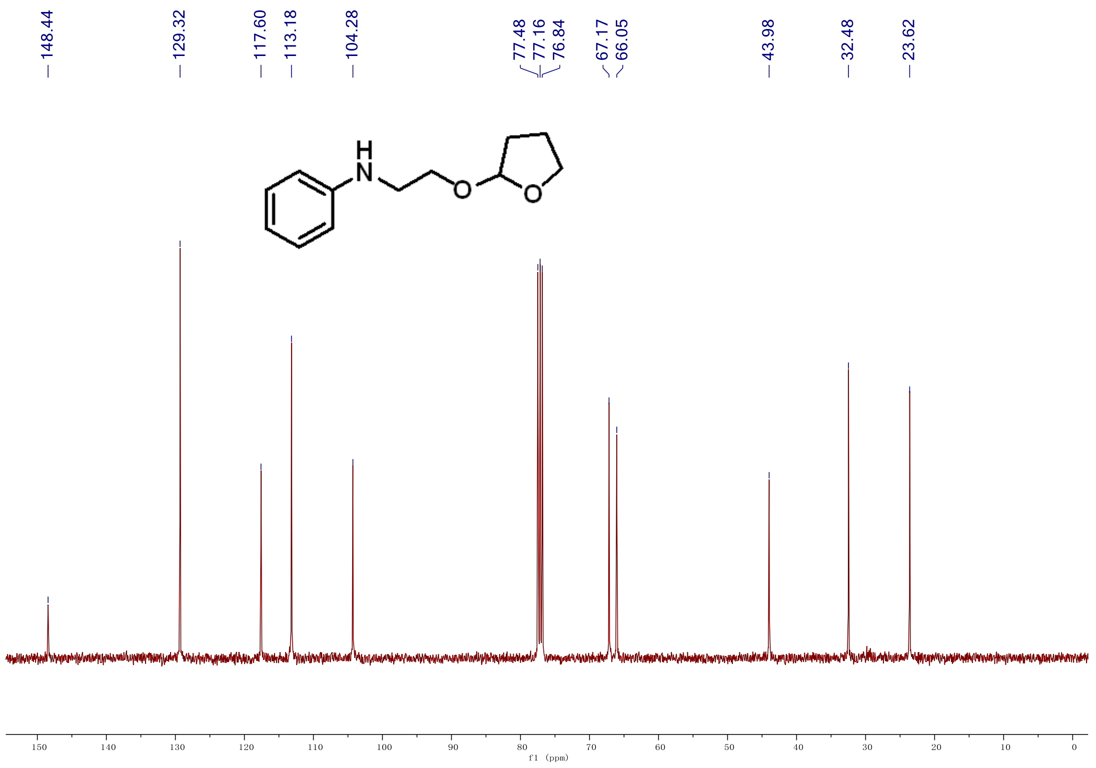
148.44
129.32
117.60
113.18
104.28
77.48
77.16
76.84
67.17
66.05
43.98
32.48
23.62
f1 (ppm)

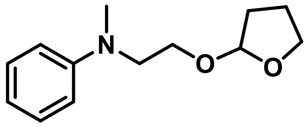

2au

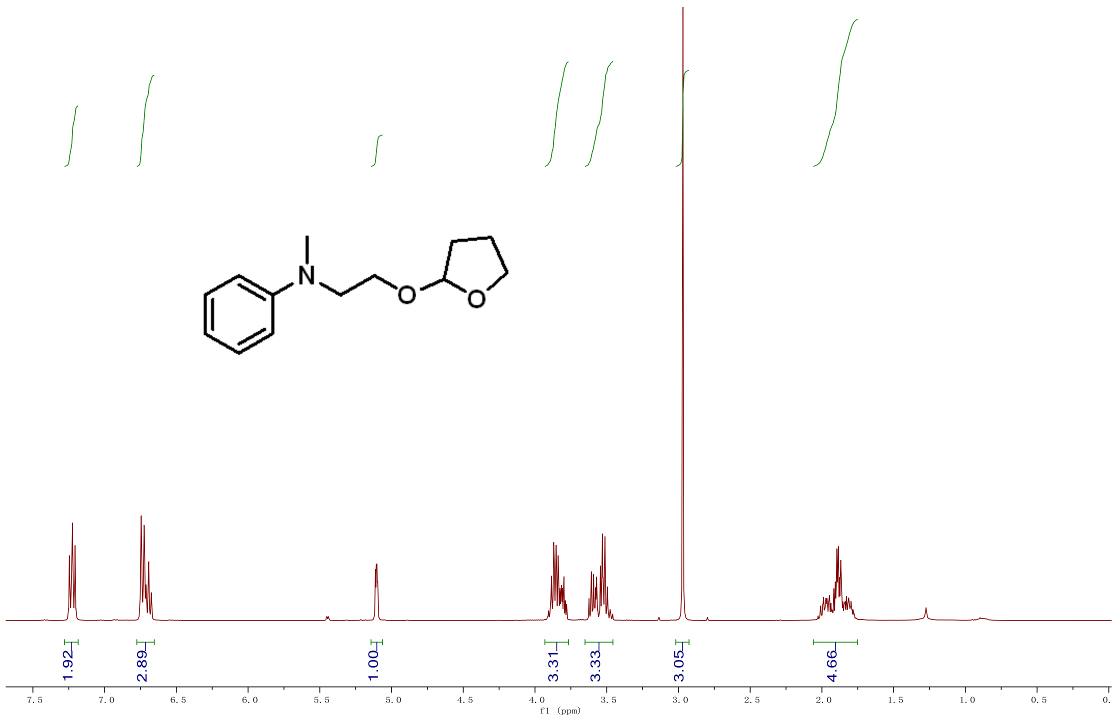
1.92
2.89
1.00
3.31
3.33
3.05
4.66
f1 (ppm)

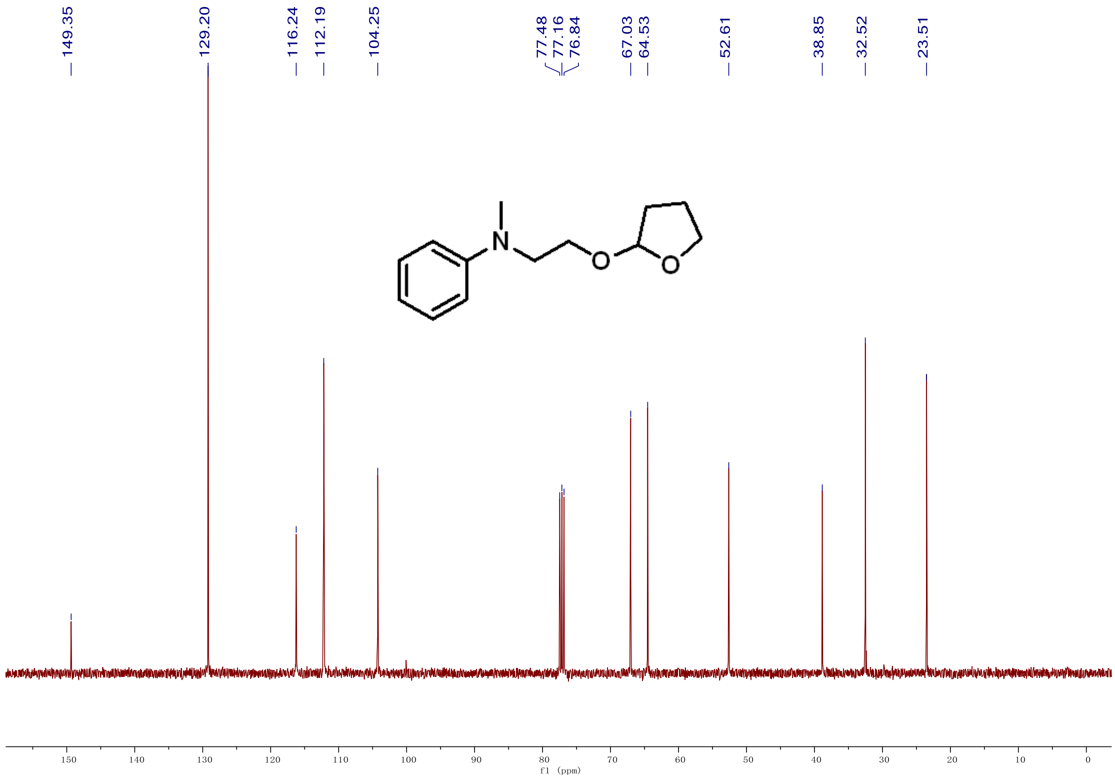
149.35
129.20
116.24
112.19
104.25
77.48
77.16
76.84
67.03
64.53
52.61
38.85
32.52
23.51
N
O
O
f1 (ppm)

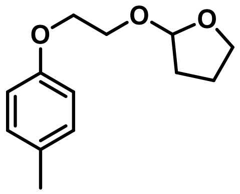
O
O
O

2av

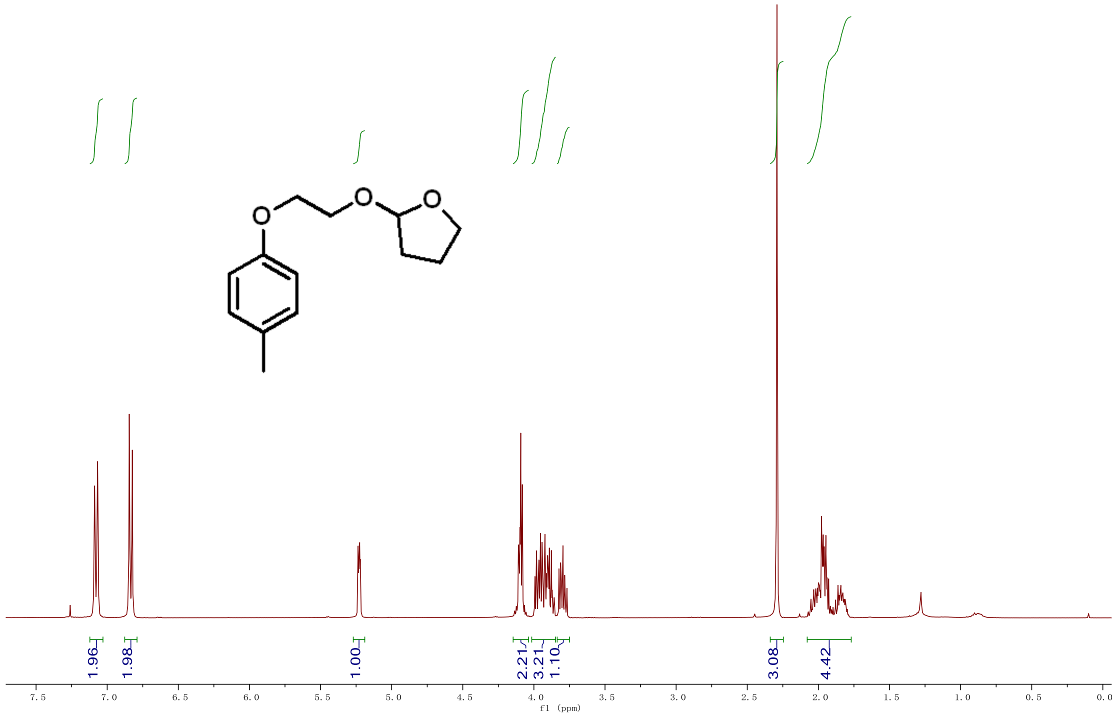
O
O
O
1.96
1.98
1.00
2.21
3.21
1.10
3.08
4.42
f1 (ppm)

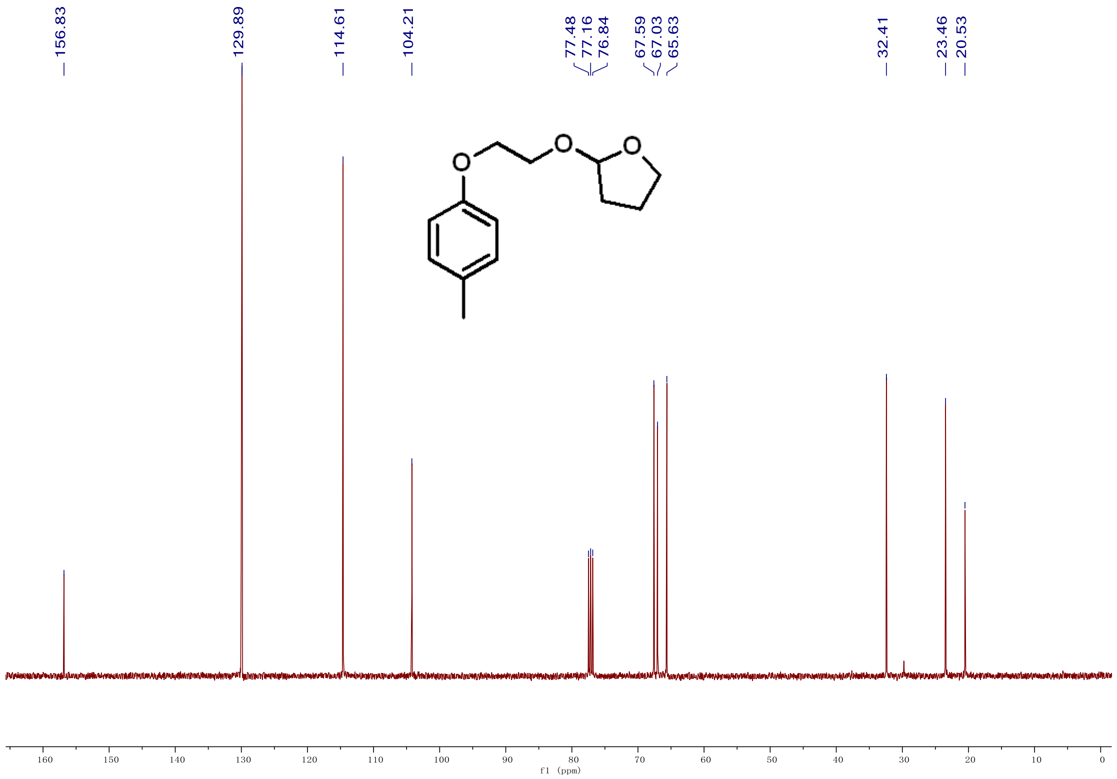
156.83
129.89
114.61
104.21
77.48
77.16
76.84
67.59
67.03
65.63
32.41
23.46
20.53
f1 (ppm)

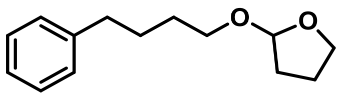

2aw

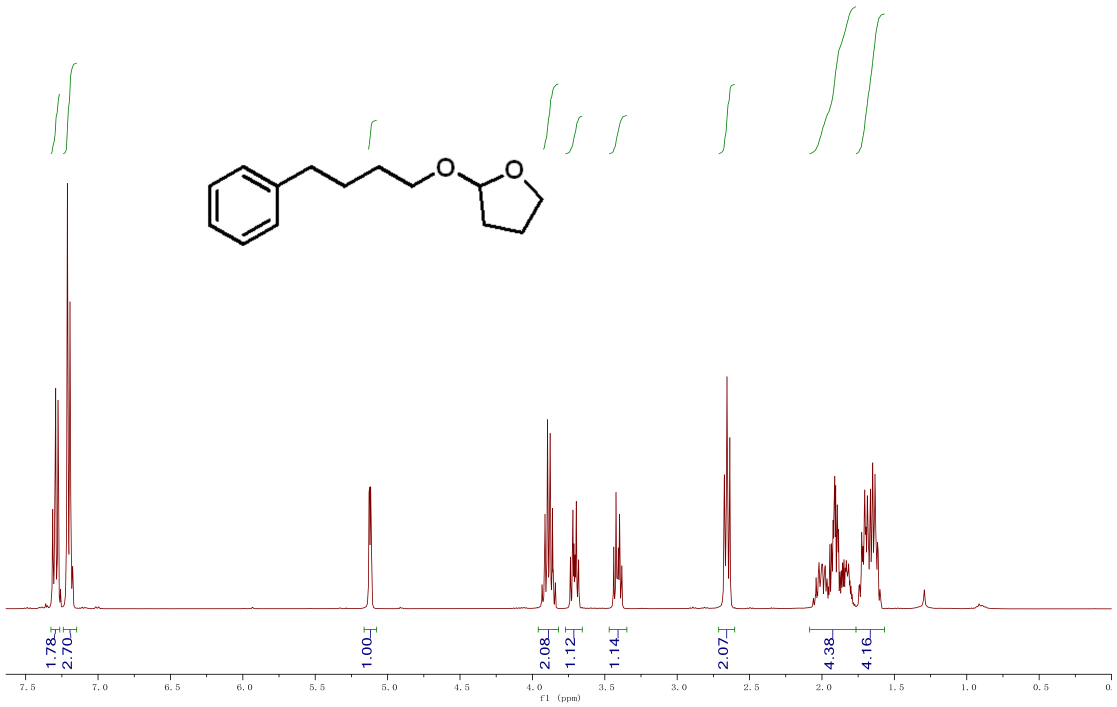
1.78
2.70
1.00
2.08
1.12
1.14
2.07
4.38
4.16
f1 (ppm)

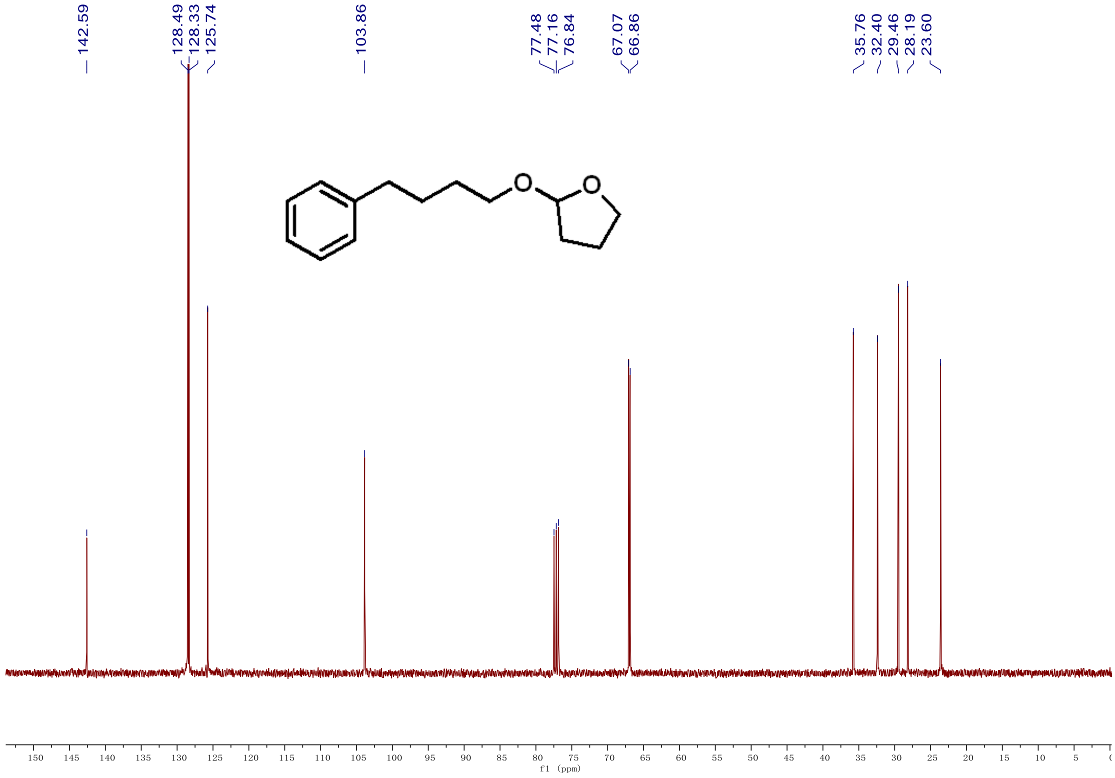
142.59
128.49
128.33
125.74
103.86
77.48
77.16
76.84
67.07
66.86
35.76
32.40
29.46
28.19
23.60
f1 (ppm)

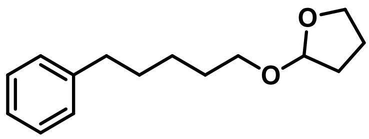

2ax

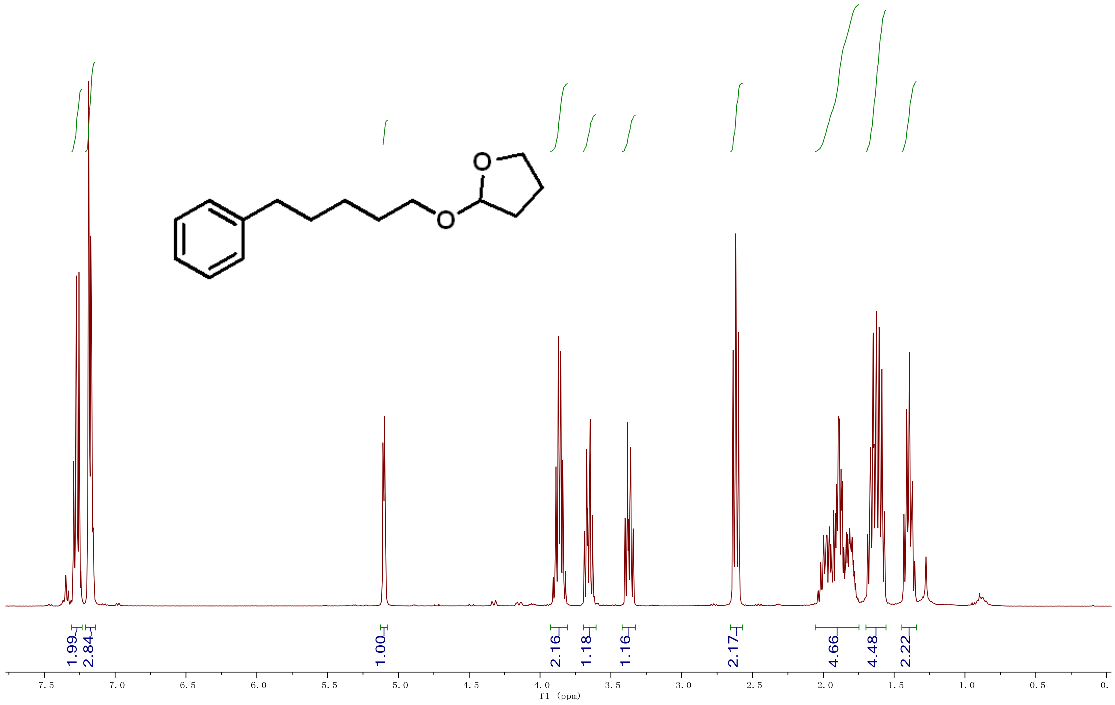
1.99
2.84
1.00
2.16
1.18
1.16
2.17
4.66
4.48
2.22
f1 (ppm)

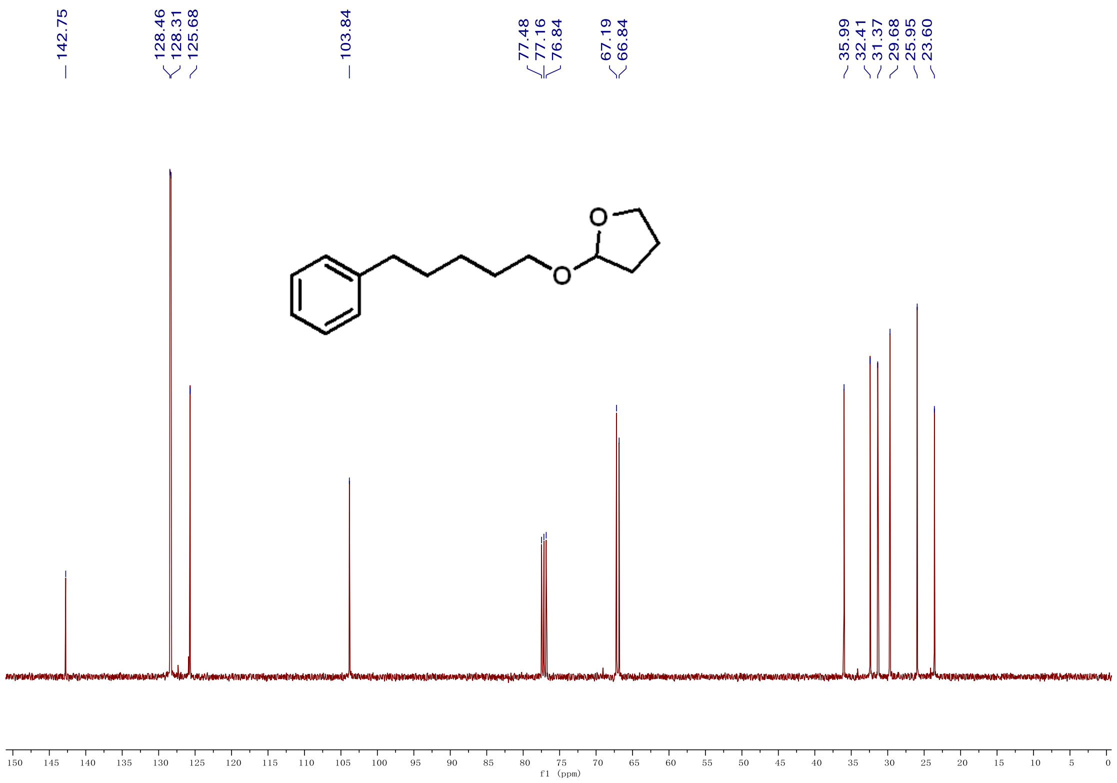
142.75
128.46
128.31
125.68
103.84
77.48
77.16
76.84
67.19
66.84
35.99
32.41
31.37
29.68
25.95
23.60
O
O
f1 (ppm)

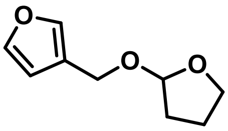
O
O
O

2ay

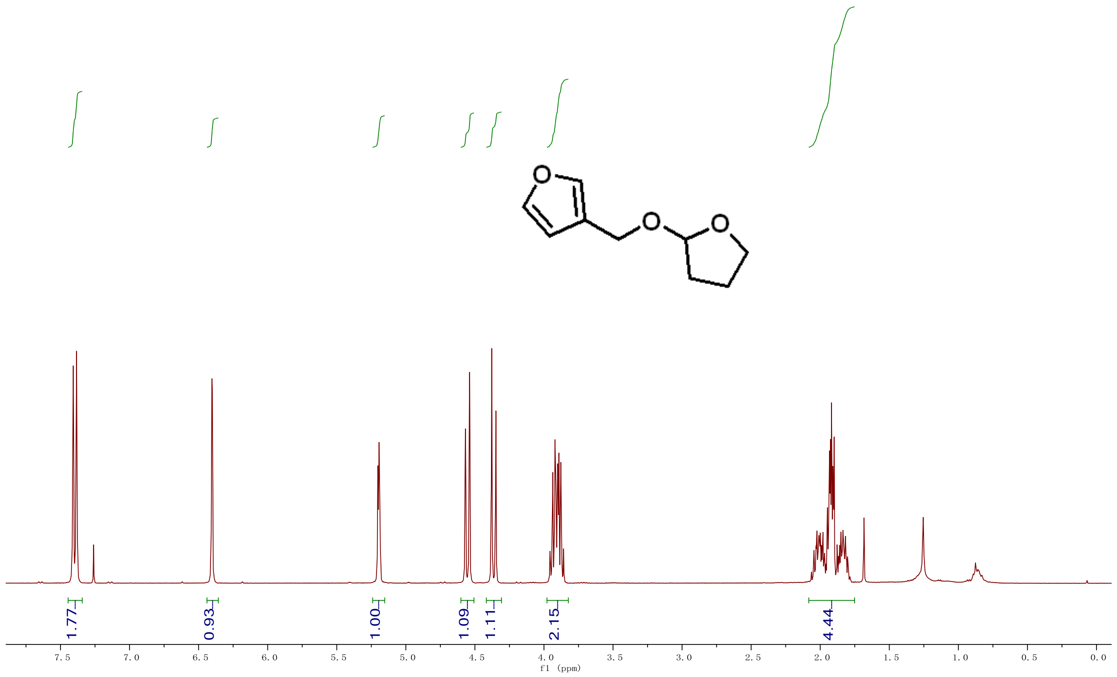
O
O
O
1.77
0.93
1.00
1.09
1.11
2.15
4.44
f1 (ppm)

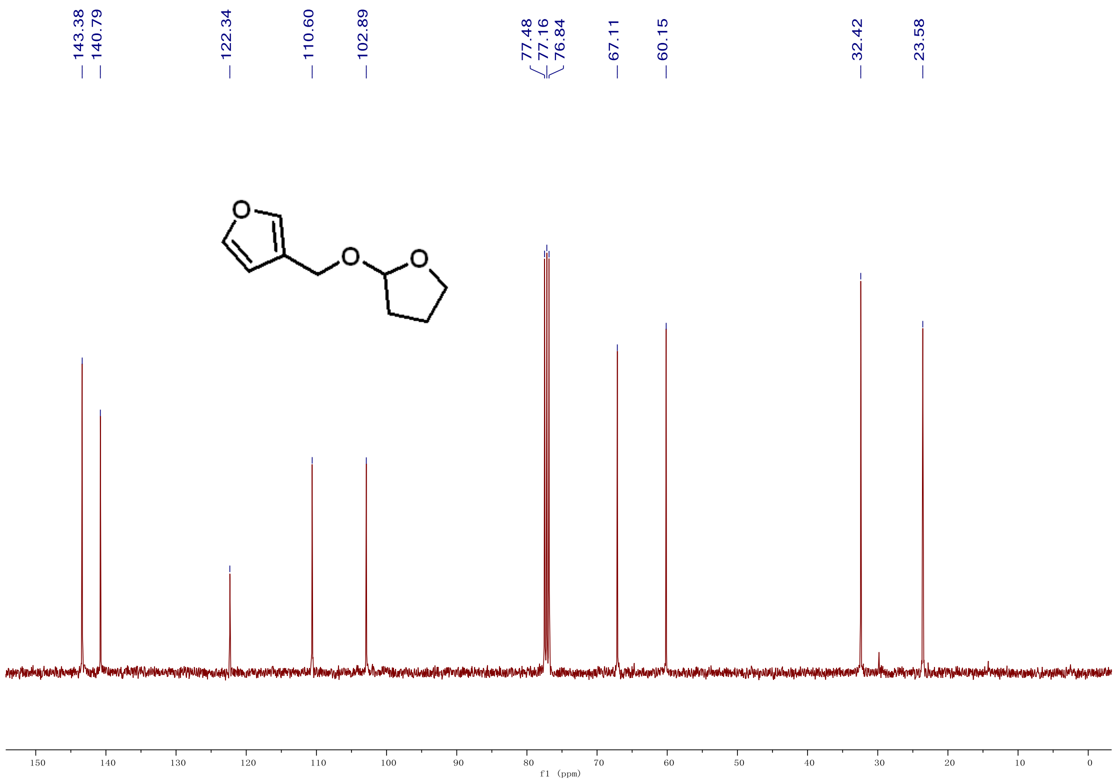
143.38
140.79
122.34
110.60
102.89
77.48
77.16
76.84
67.11
60.15
32.42
23.58
f1 (ppm)

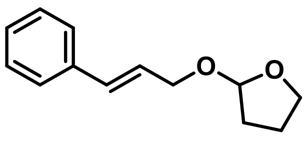

2az

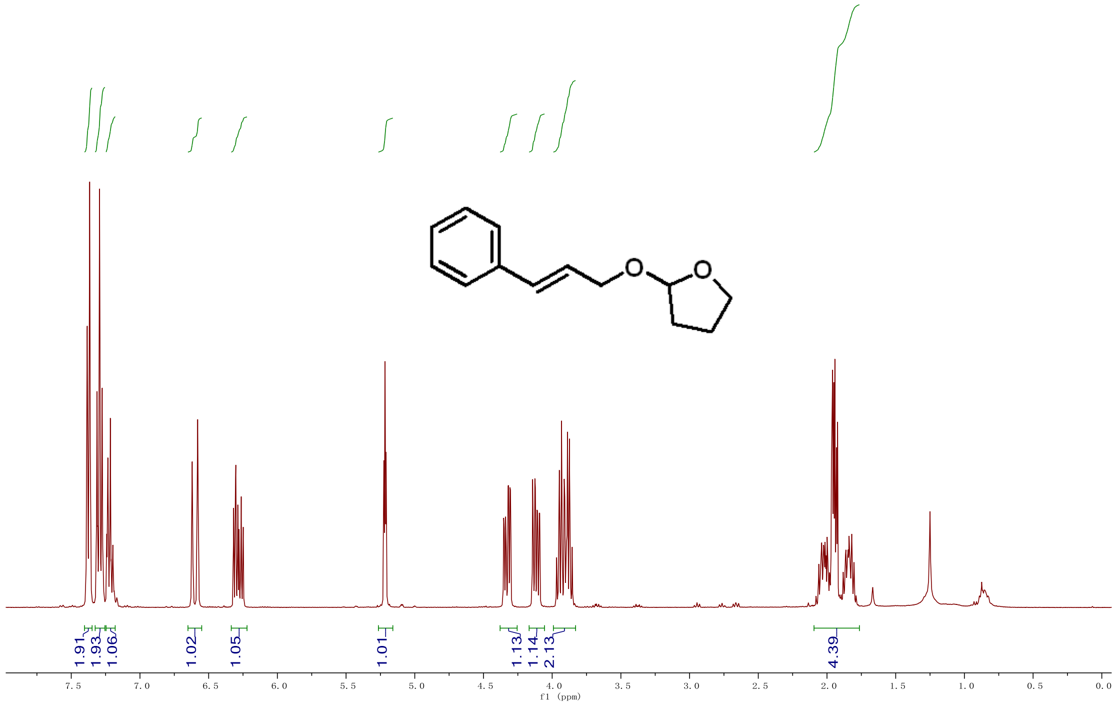
1.91
1.93
1.06
1.02
1.05
1.01
1.13
1.14
2.13
4.39
f1 (ppm)

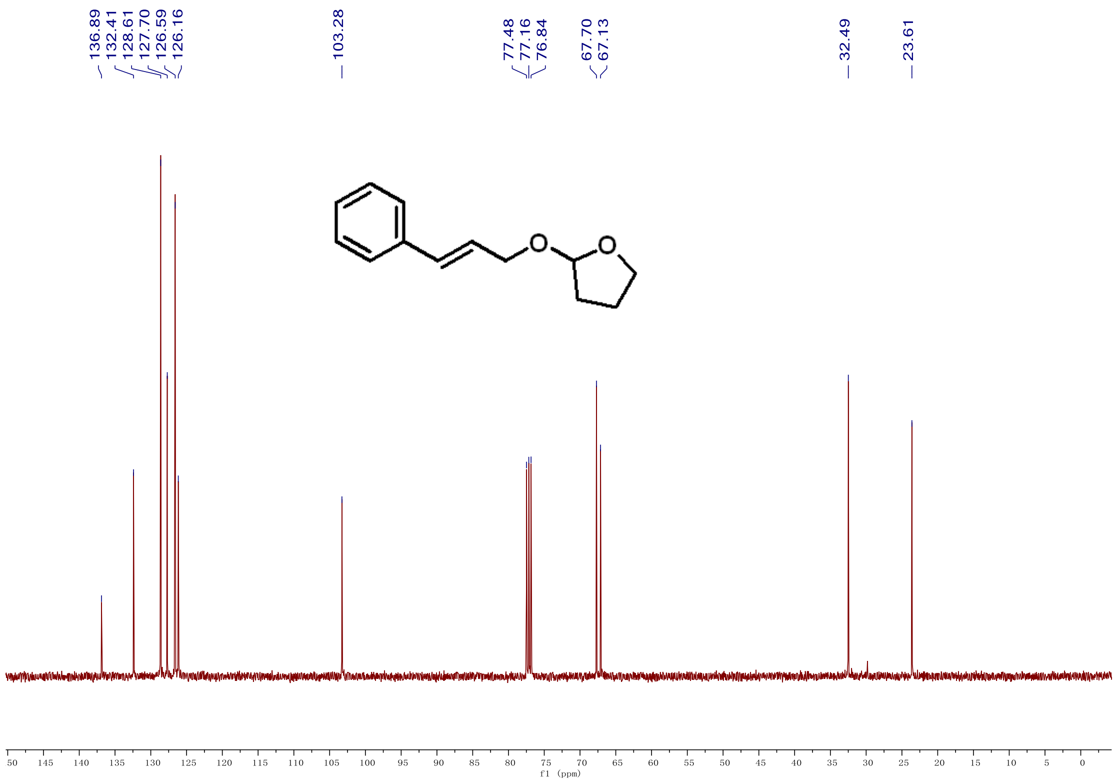
136.89
132.41
128.61
127.70
126.59
126.16
103.28
77.48
77.16
76.84
67.70
67.13
32.49
23.61
f1 (ppm)

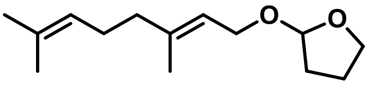

2ba

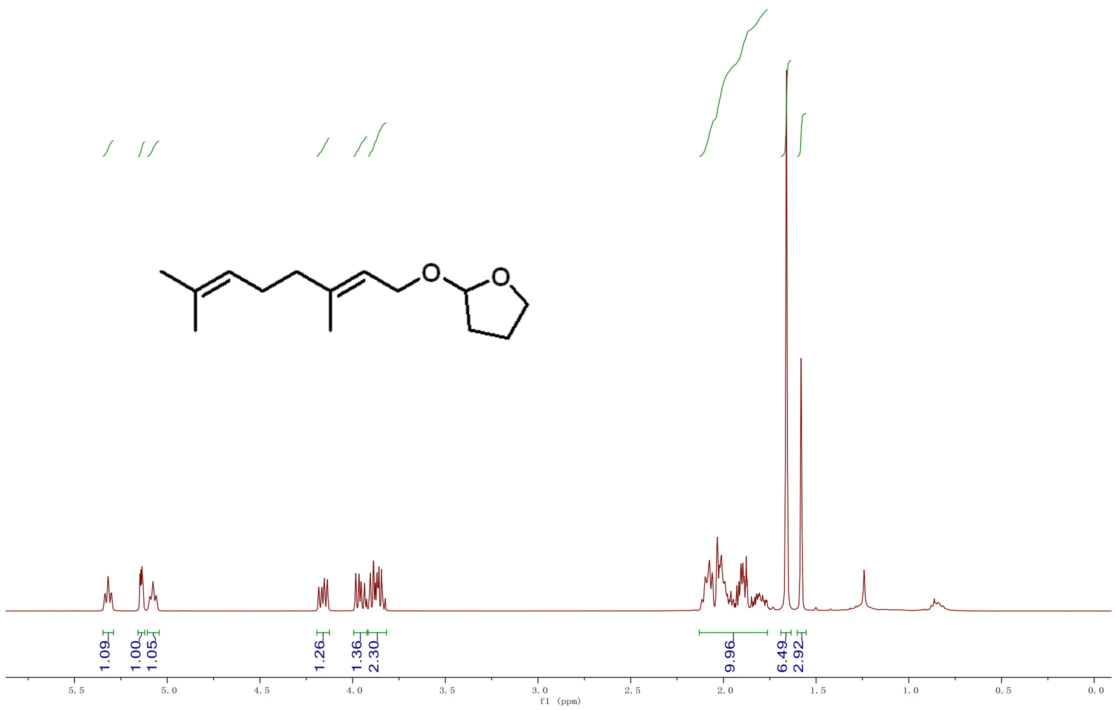
1.09
1.00
1.05
1.26
1.36
2.30
9.96
6.49
2.92
f1 (ppm)

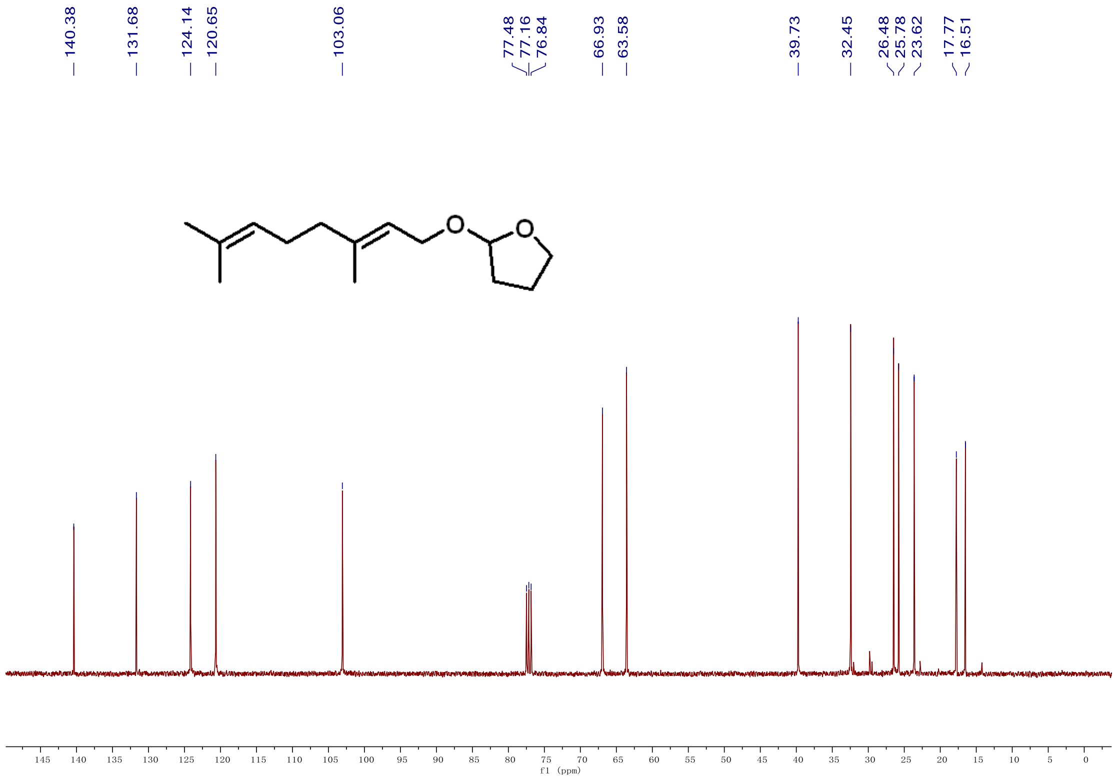
140.38
131.68
124.14
120.65
103.06
77.48
77.16
76.84
66.93
63.58
39.73
32.45
26.48
25.78
23.62
17.77
16.51
f1 (ppm)

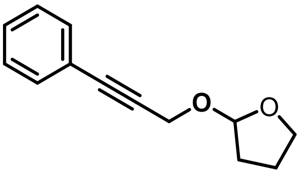

2bb

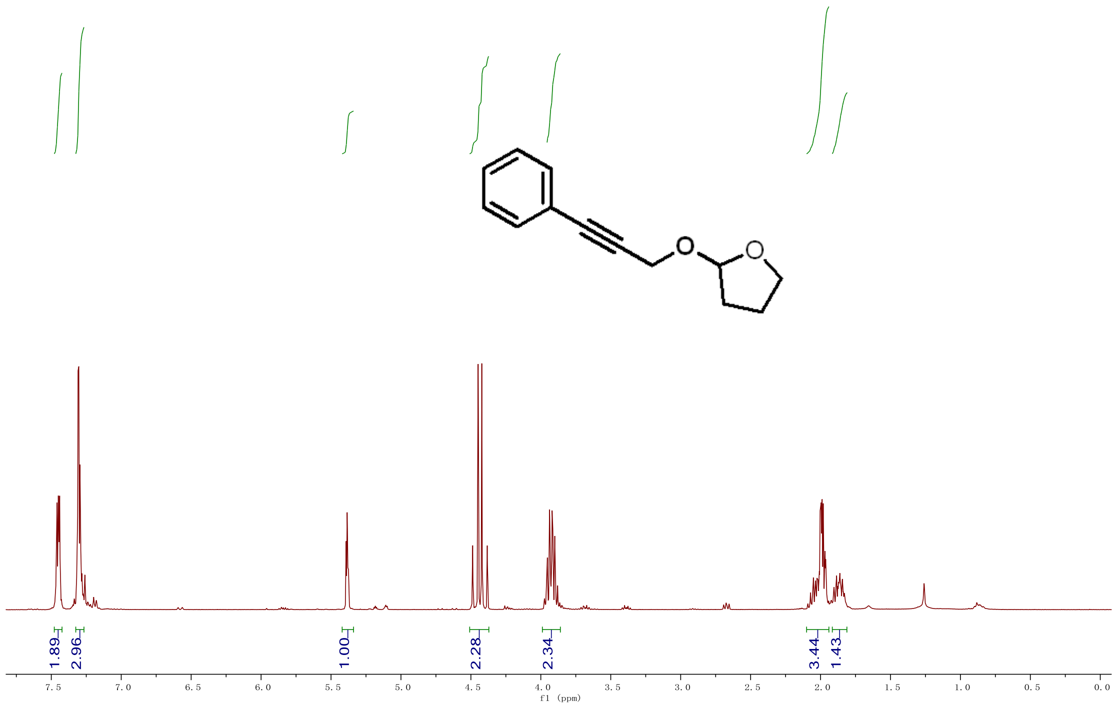
1.89
2.96
1.00
2.28
2.34
3.44
1.43
f1 (ppm)

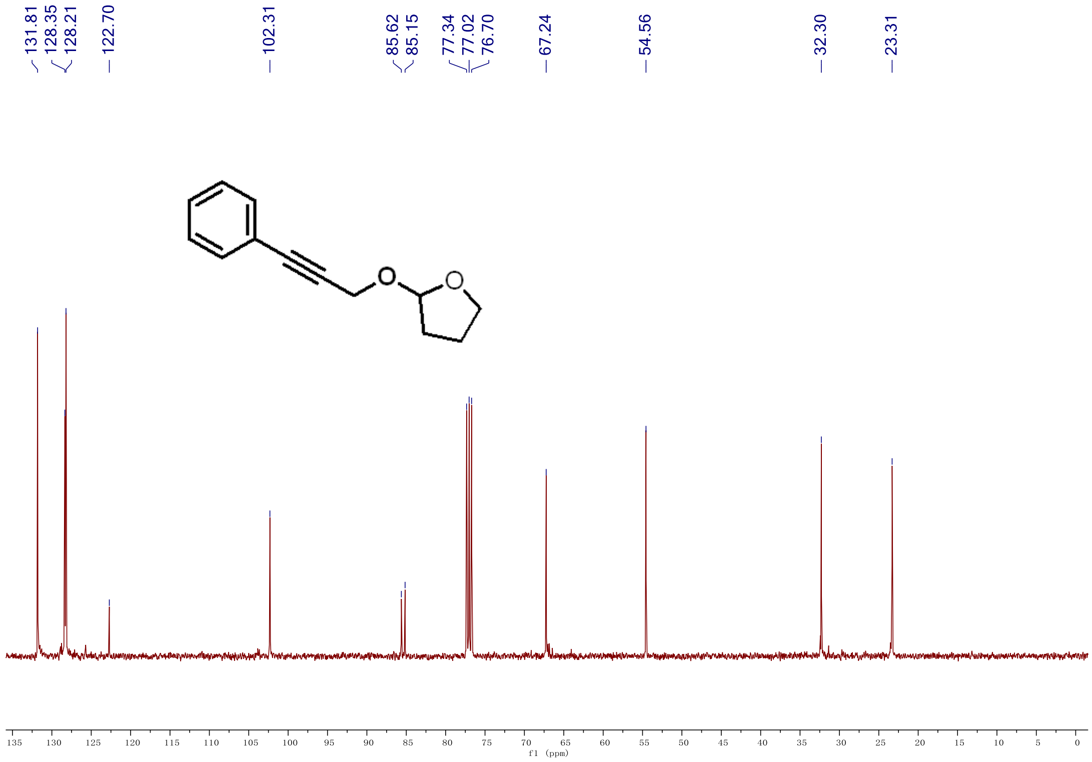
131.81
128.35
128.21
122.70
102.31
85.62
85.15
77.34
77.02
76.70
67.24
54.56
32.30
23.31
f1 (ppm)

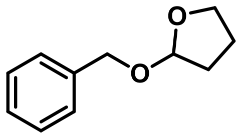

2bc

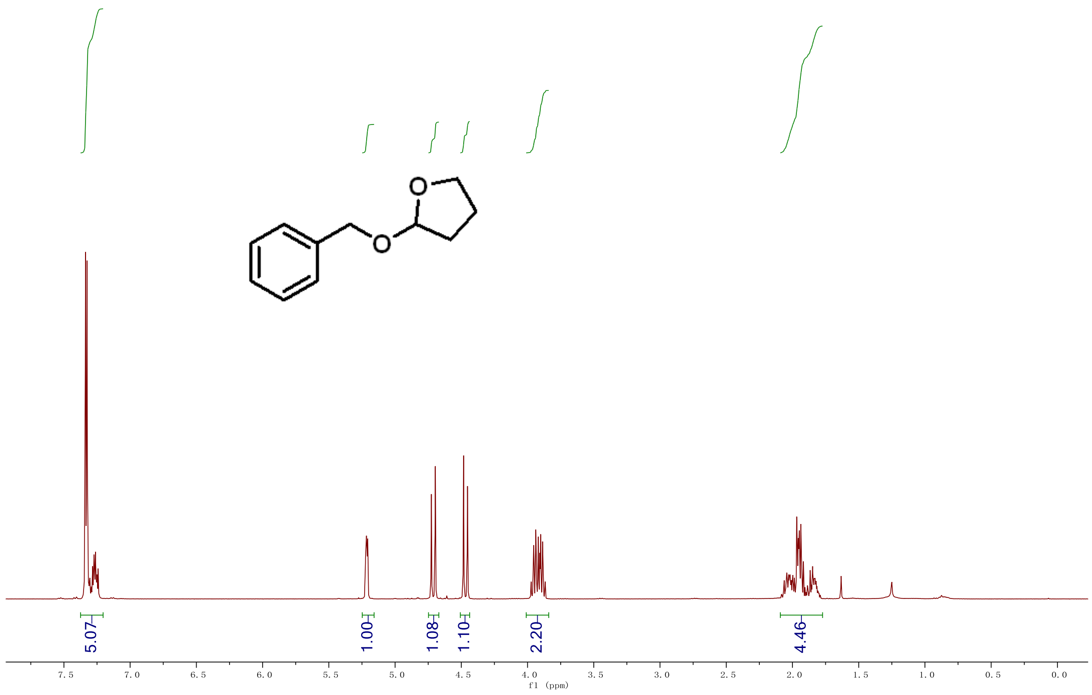
5.07
1.00
1.08
1.10
2.20
4.46
f1 (ppm)

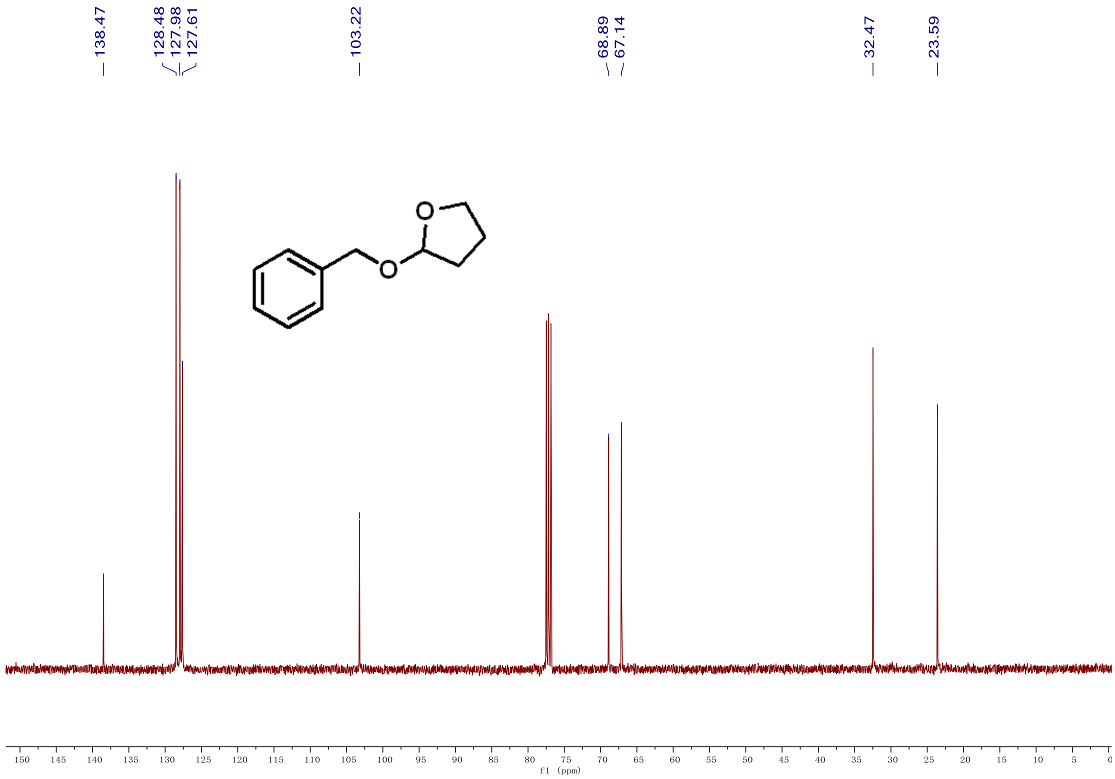
138.47
128.48
127.98
127.61
103.22
68.89
67.14
32.47
23.59
O
O
150 145 140 135 130 125 120 115 110 105 100 95 90 85 80 75 70 65 60 55 50 45 40 35 30 25 20 15 10 5 0
f1 (ppm)

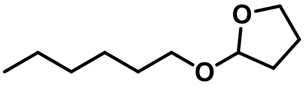
O
O

2bd

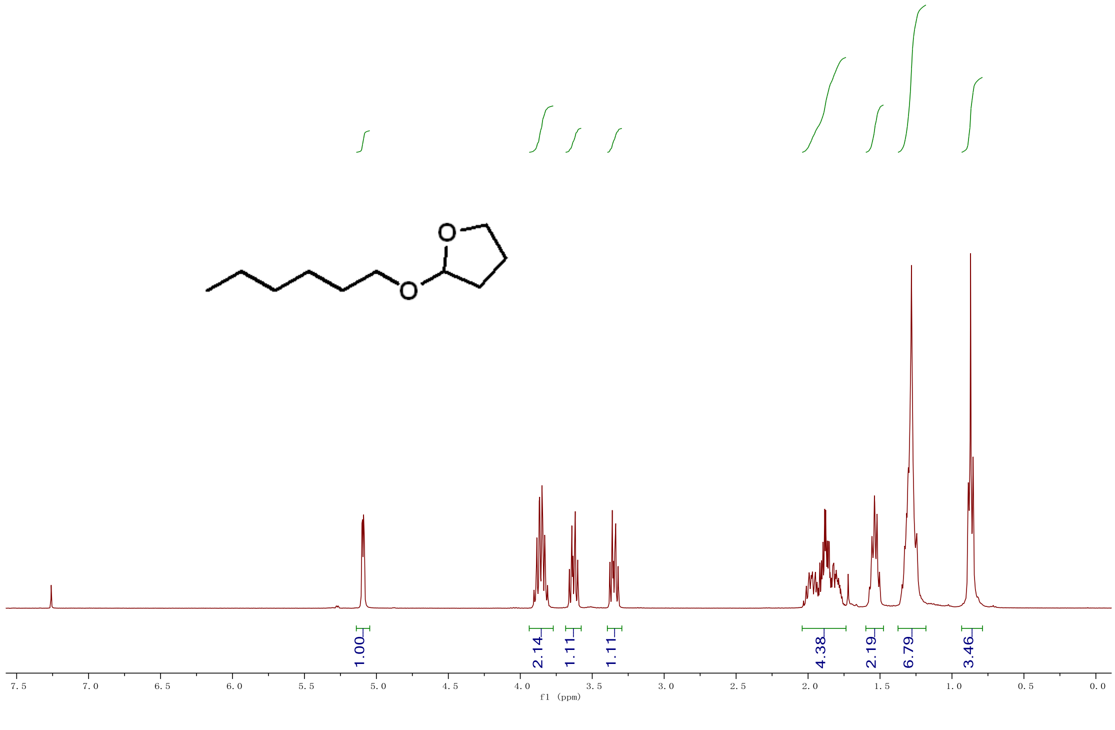
O
O
1.00
2.14
1.11
1.11
4.38
2.19
6.79
3.46
7.5 7.0 6.5 6.0 5.5 5.0 4.5 4.0 3.5 3.0 2.5 2.0 1.5 1.0 0.5 0.0
f1 (ppm)

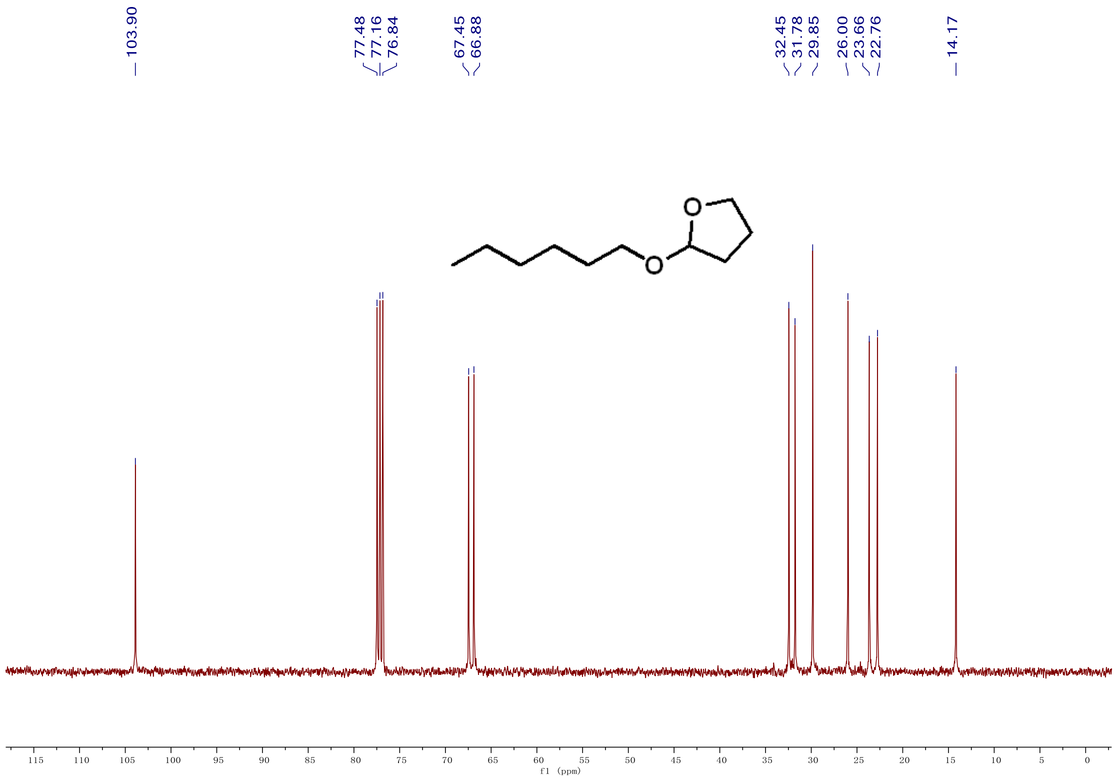
103.90
77.48
77.16
76.84
67.45
66.88
32.45
31.78
29.85
26.00
23.66
22.76
14.17
f1 (ppm)

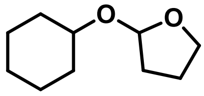

2be

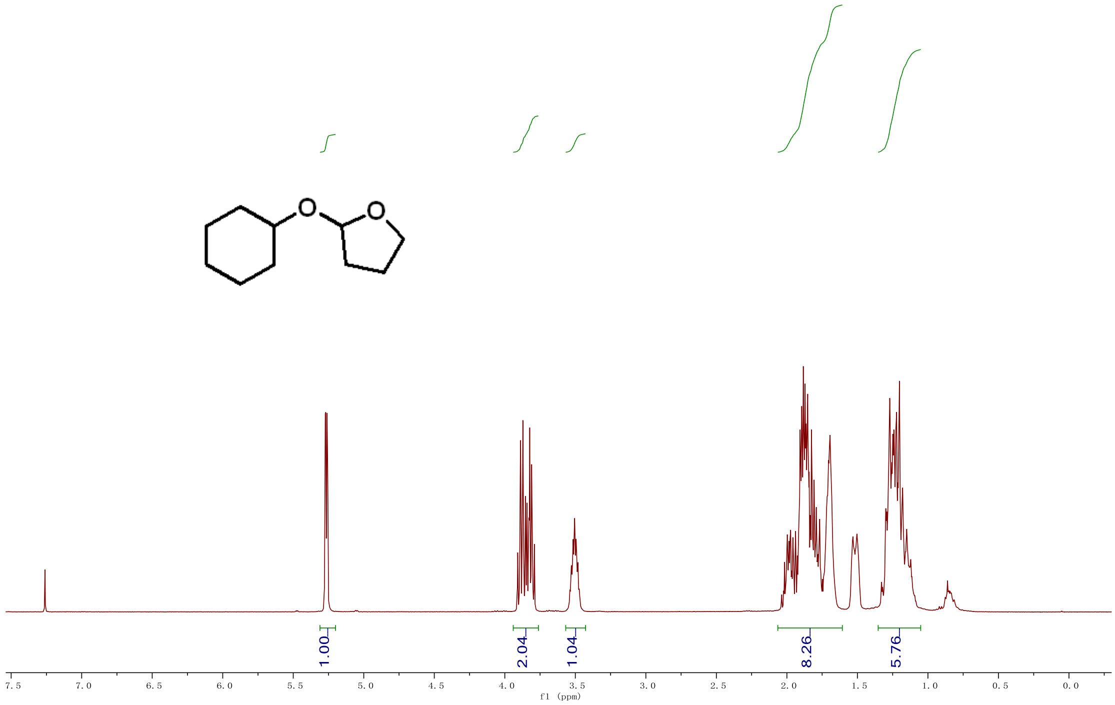
1.00
2.04
1.04
8.26
5.76
f1 (ppm)

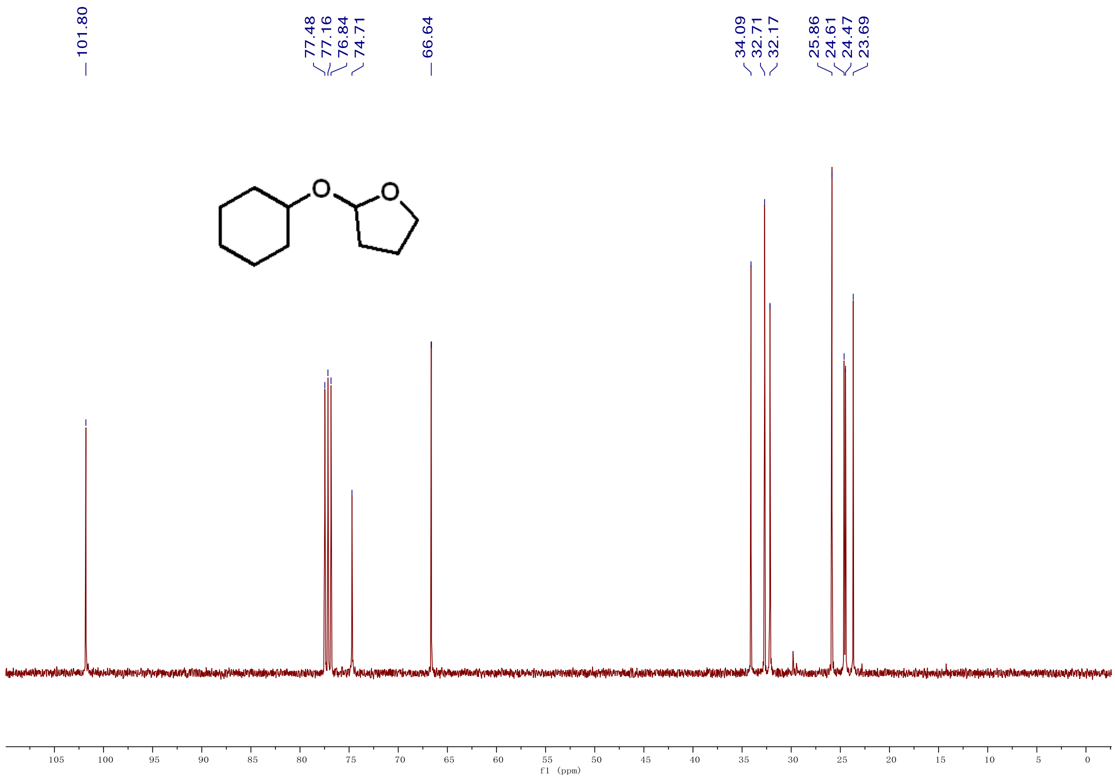
101.80
77.48
77.16
76.84
74.71
66.64
34.09
32.71
32.17
25.86
24.61
24.47
23.69
f1 (ppm)

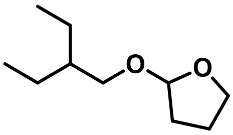

2bf

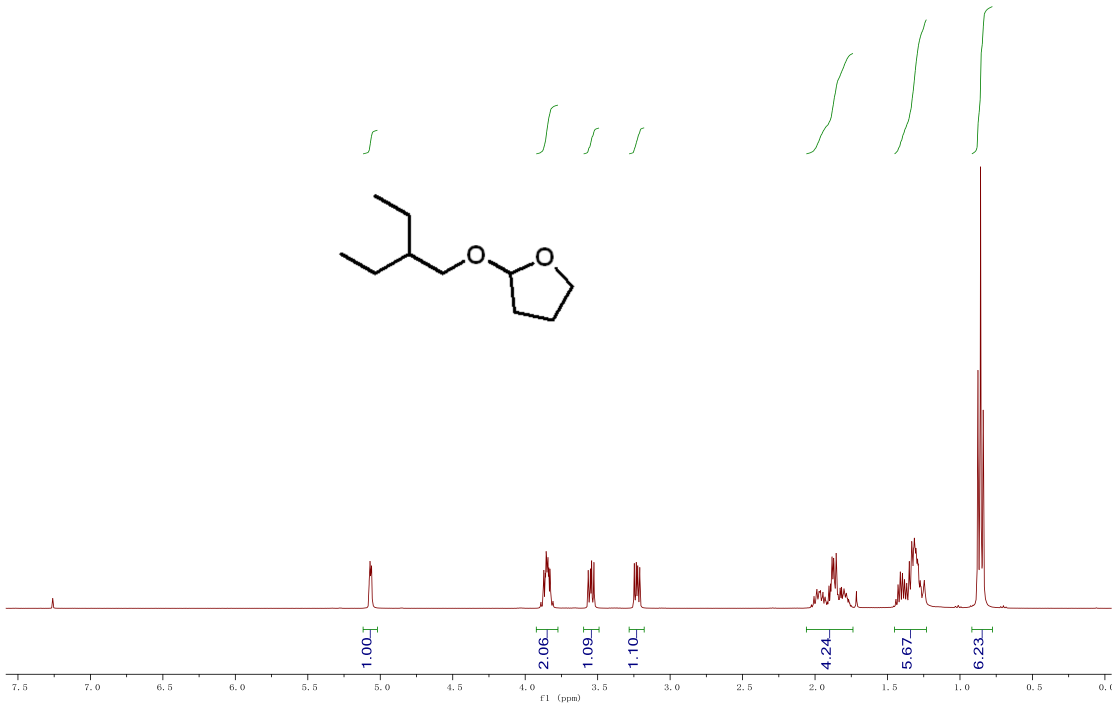
1.00
2.06
1.09
1.10
4.24
5.67
6.23
f1 (ppm)

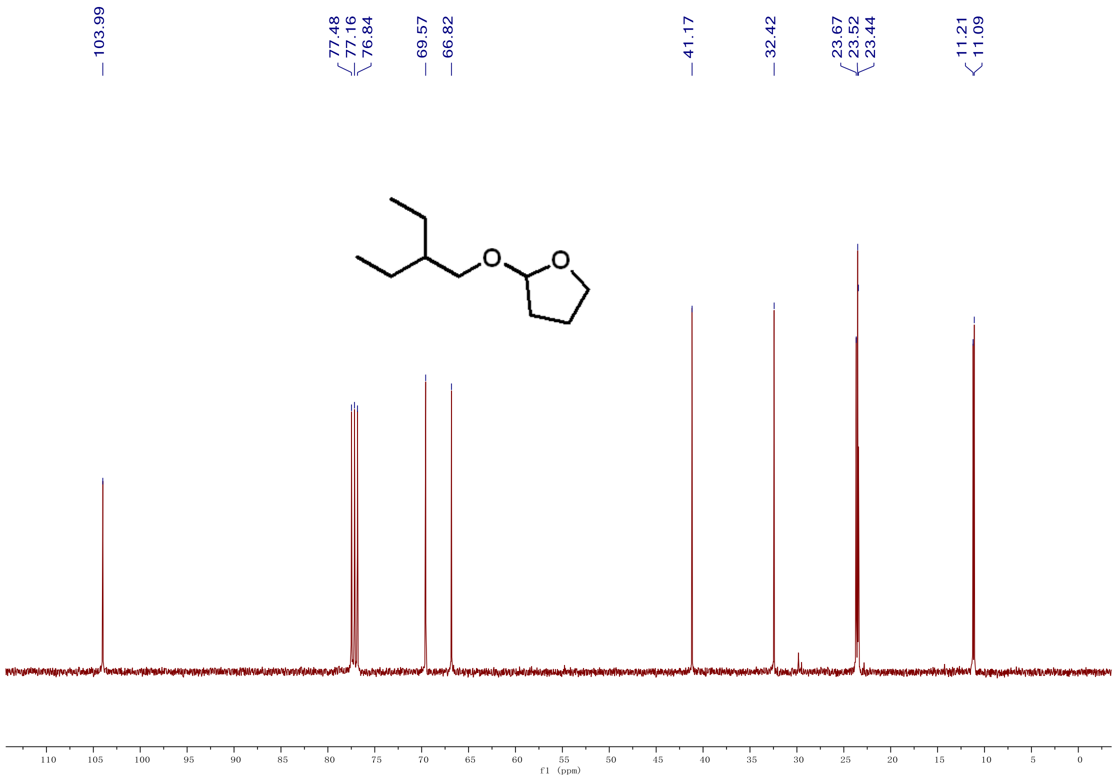
103.99
77.48
77.16
76.84
69.57
66.82
41.17
32.42
23.67
23.52
23.44
11.21
11.09
O
O
f1 (ppm)

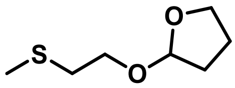
S
O
O
2bg

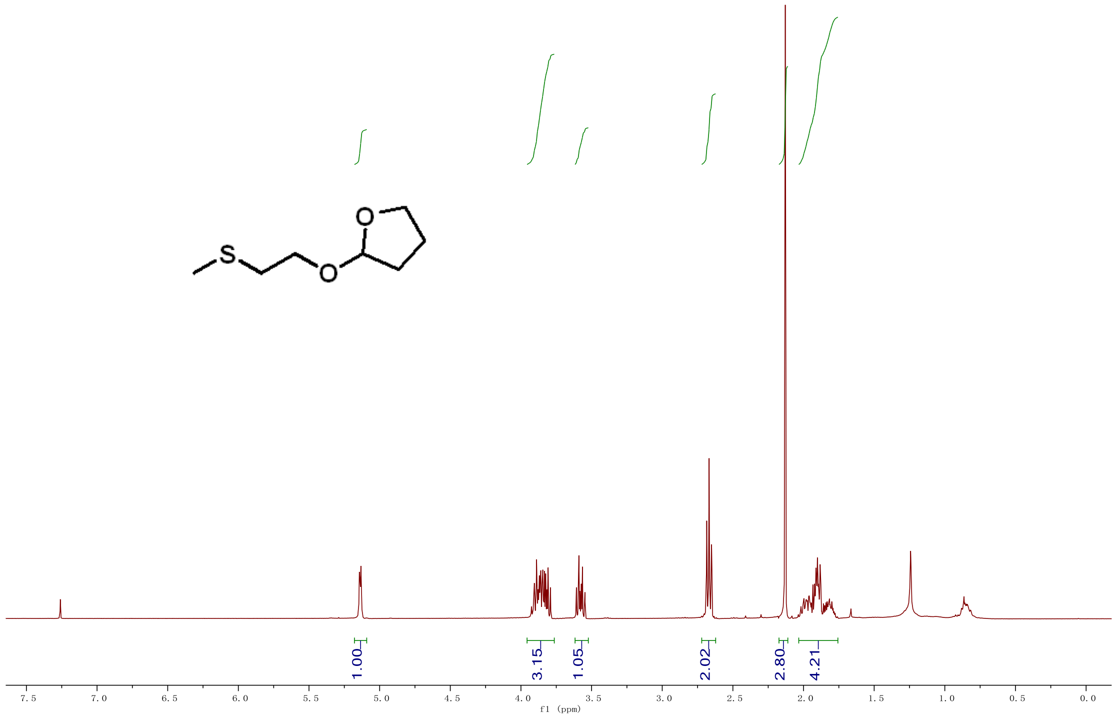
S
O
O
1.00
3.15
1.05
2.02
2.80
4.21
f1 (ppm)

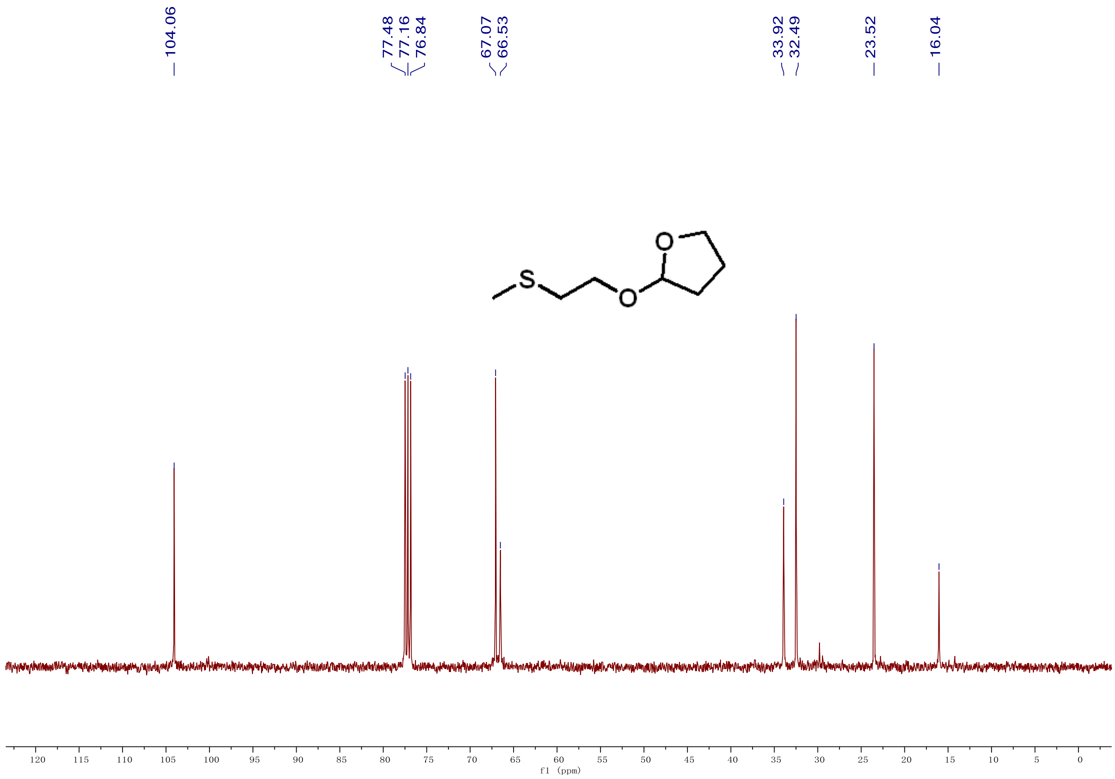
104.06
77.48
77.16
76.84
67.07
66.53
33.92
32.49
23.52
16.04
S
O
O
f1 (ppm)

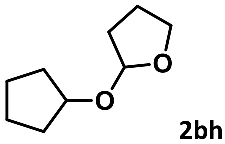
O
O

2bh

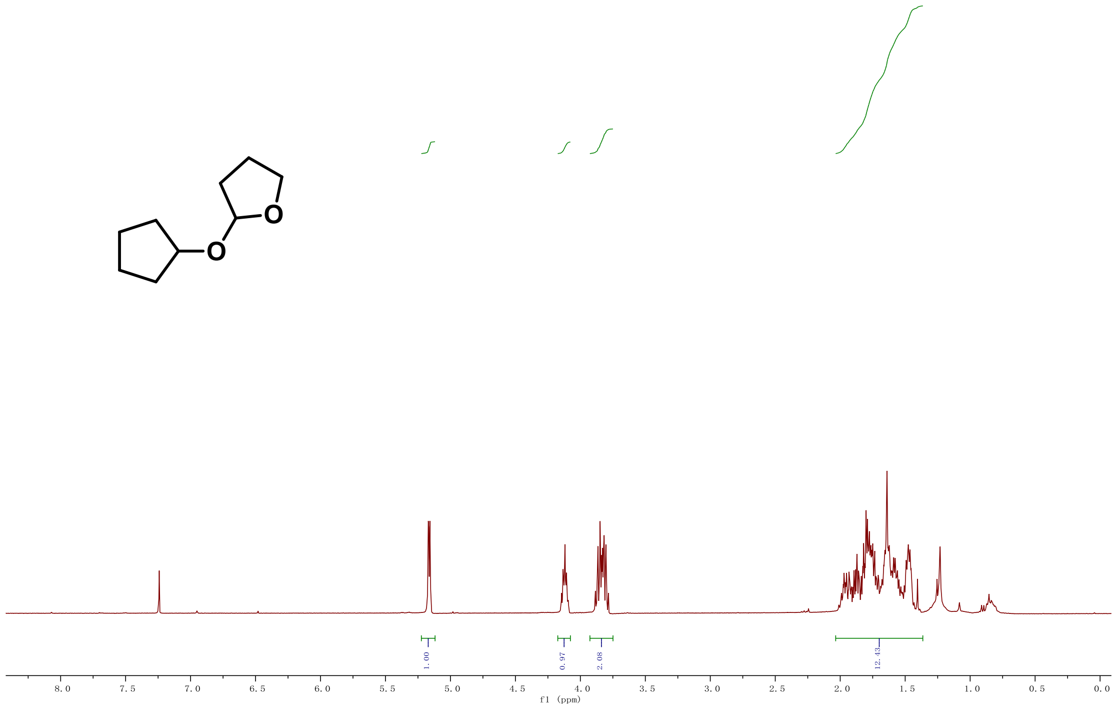
O
O
1.00
0.97
2.08
12.43
f1 (ppm)

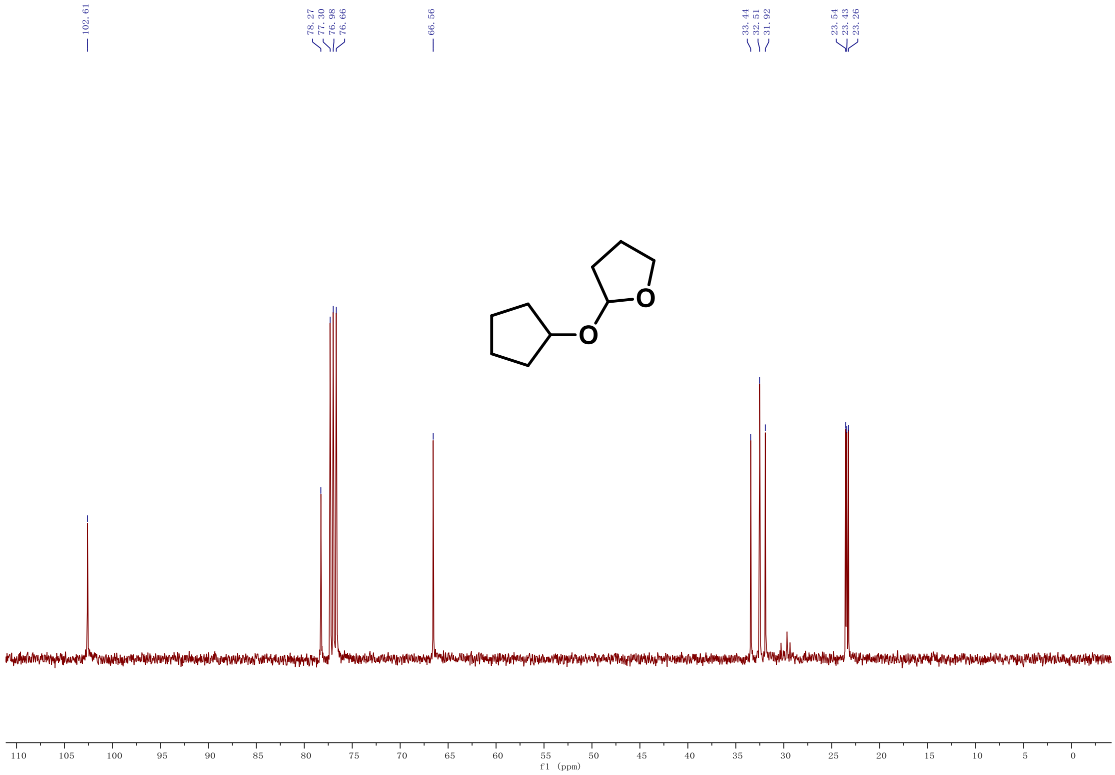
102.61
78.27
77.30
76.98
76.66
66.56
33.44
32.51
31.92
23.54
23.43
23.26
O
O
f1 (ppm)

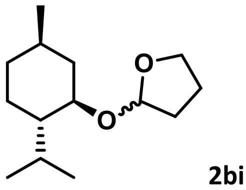

2bi

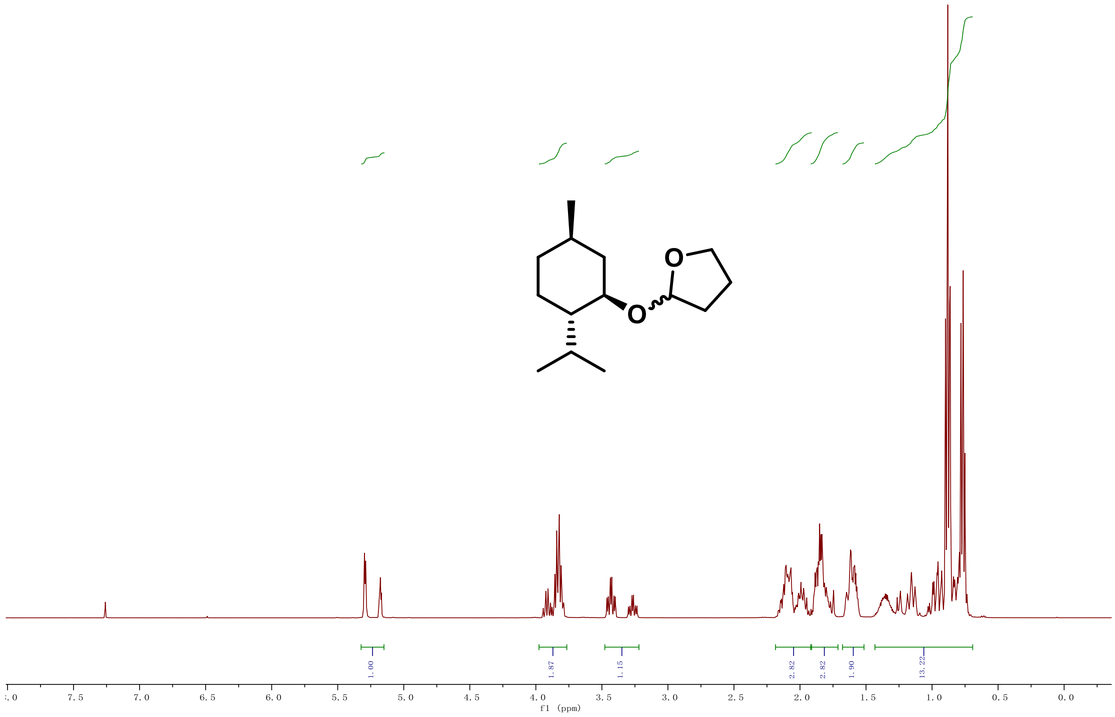
O
O
1.00
1.87
1.15
2.82
2.82
1.90
13.22
f1 (ppm)

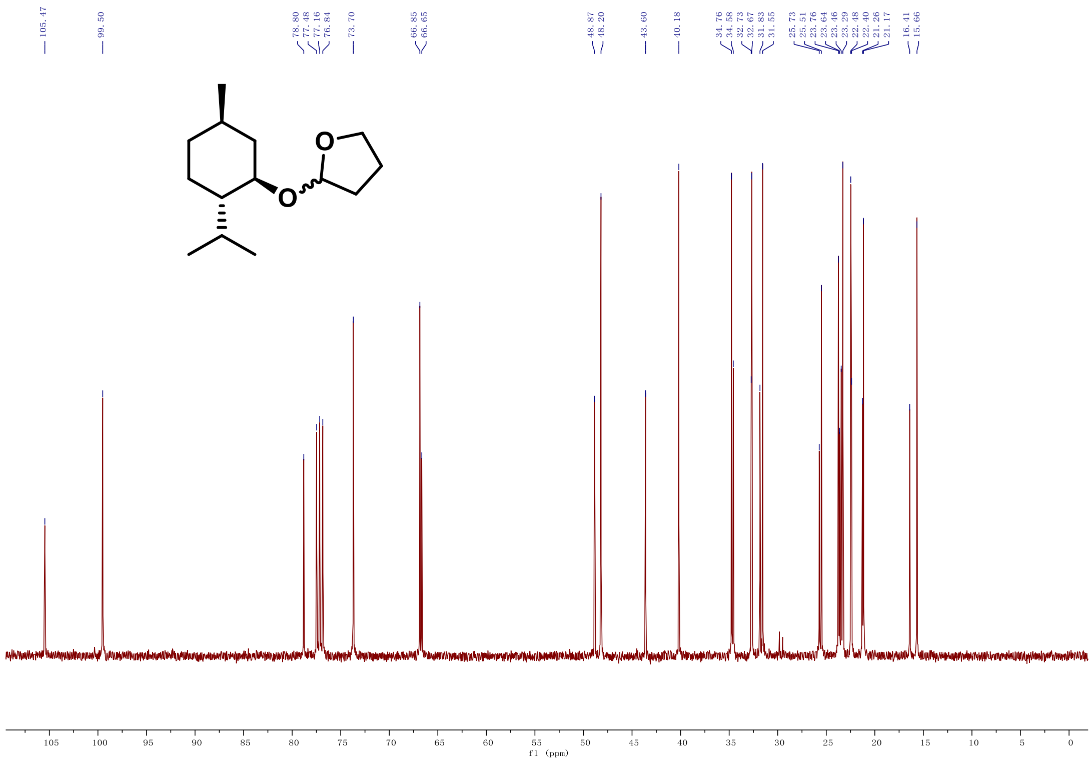
105.47
99.50
78.80
77.48
77.16
76.84
73.70
66.85
66.65
48.87
48.20
43.60
40.18
34.76
34.58
32.73
32.67
31.83
31.55
25.73
25.51
23.76
23.64
23.46
23.29
22.48
22.40
21.26
21.17
16.41
15.66
O
O
f1 (ppm)

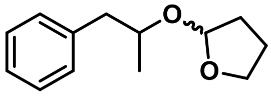
O
O

2bj

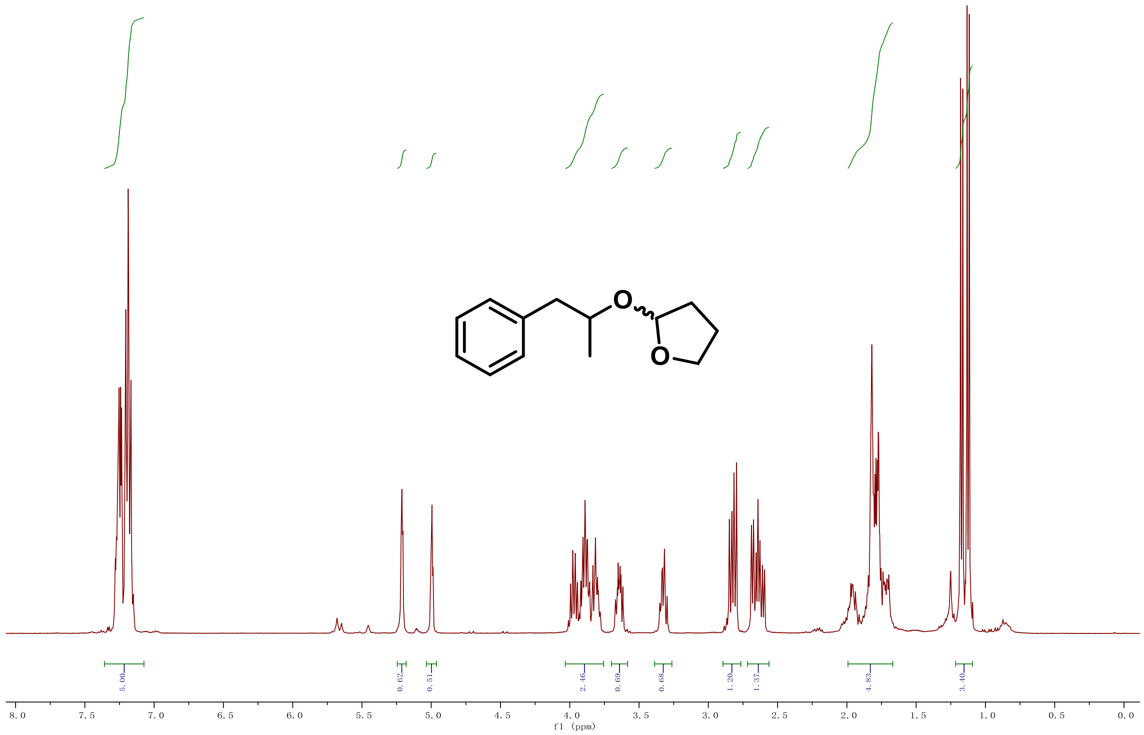
O
O
5.00
0.62
0.51
2.46
0.69
0.68
1.20
1.37
4.83
3.40
f1 (ppm)

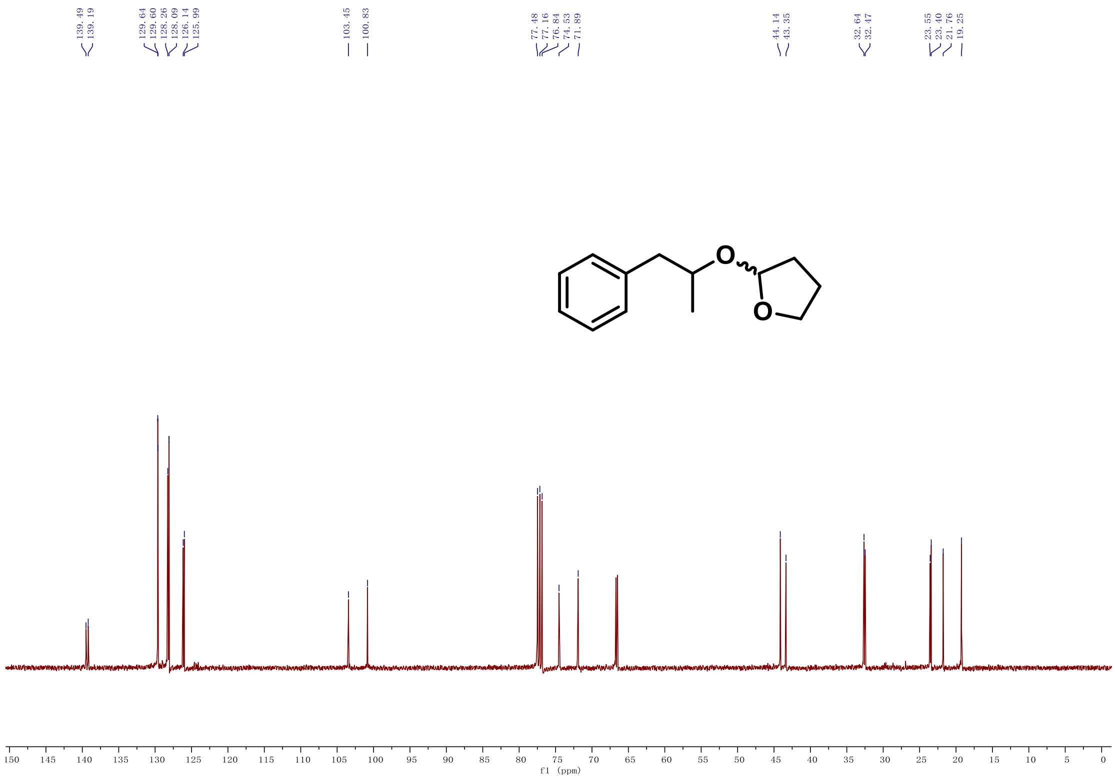
139.49
139.19
129.64
129.60
128.36
128.09
126.14
125.99
103.45
100.83
77.48
77.16
76.84
74.53
71.89
44.14
43.35
32.64
32.47
23.55
23.40
21.76
19.25
O
O
f1 (ppm)

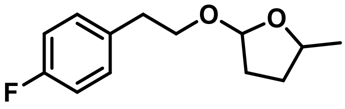
F
O
O

3ad

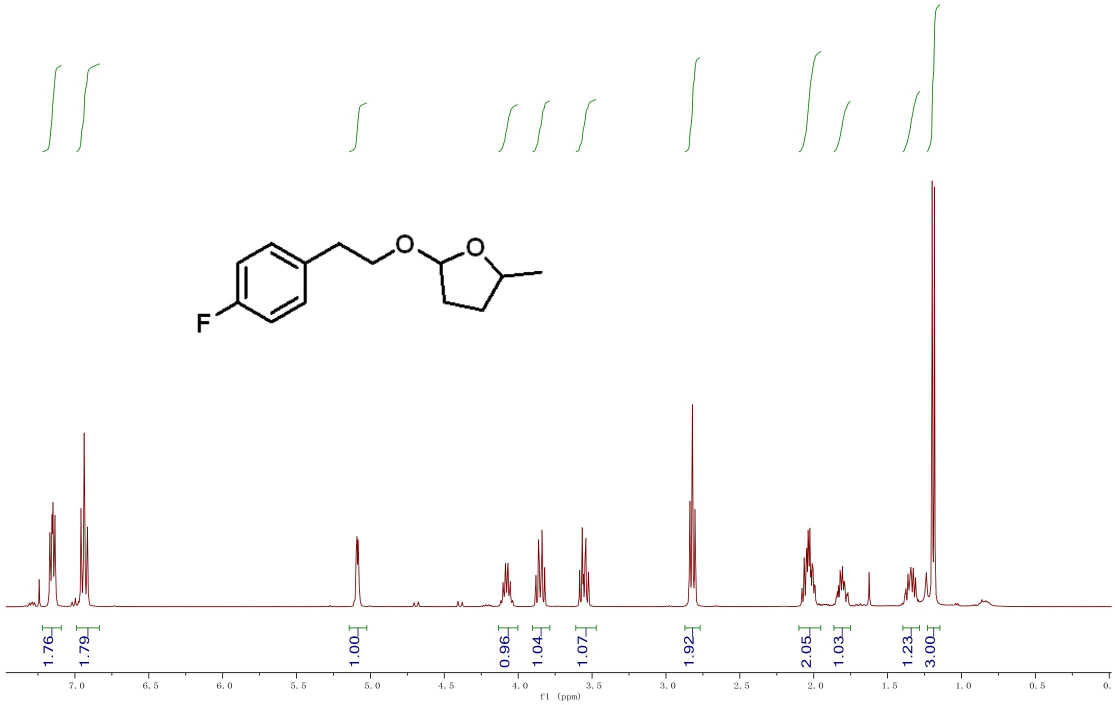
F
O
O
1.76
1.79
1.00
0.96
1.04
1.07
1.92
2.05
1.03
1.23
3.00
f1 (ppm)

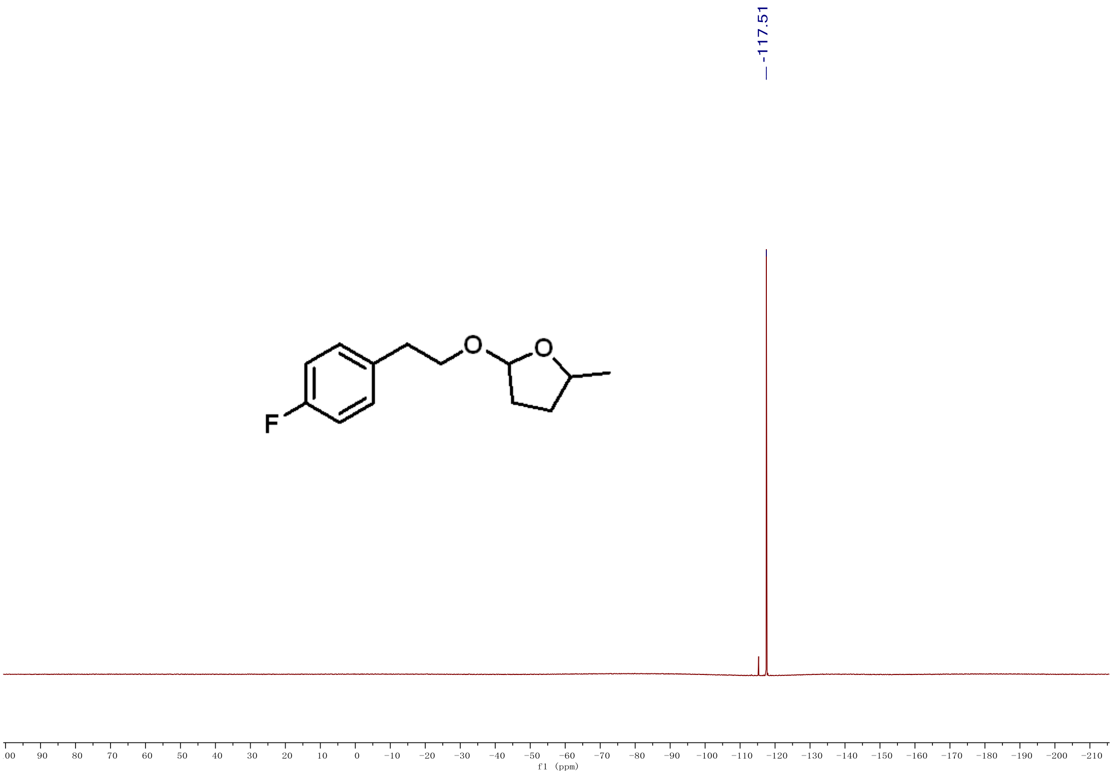
-117.51
F
O
O
f1 (ppm)

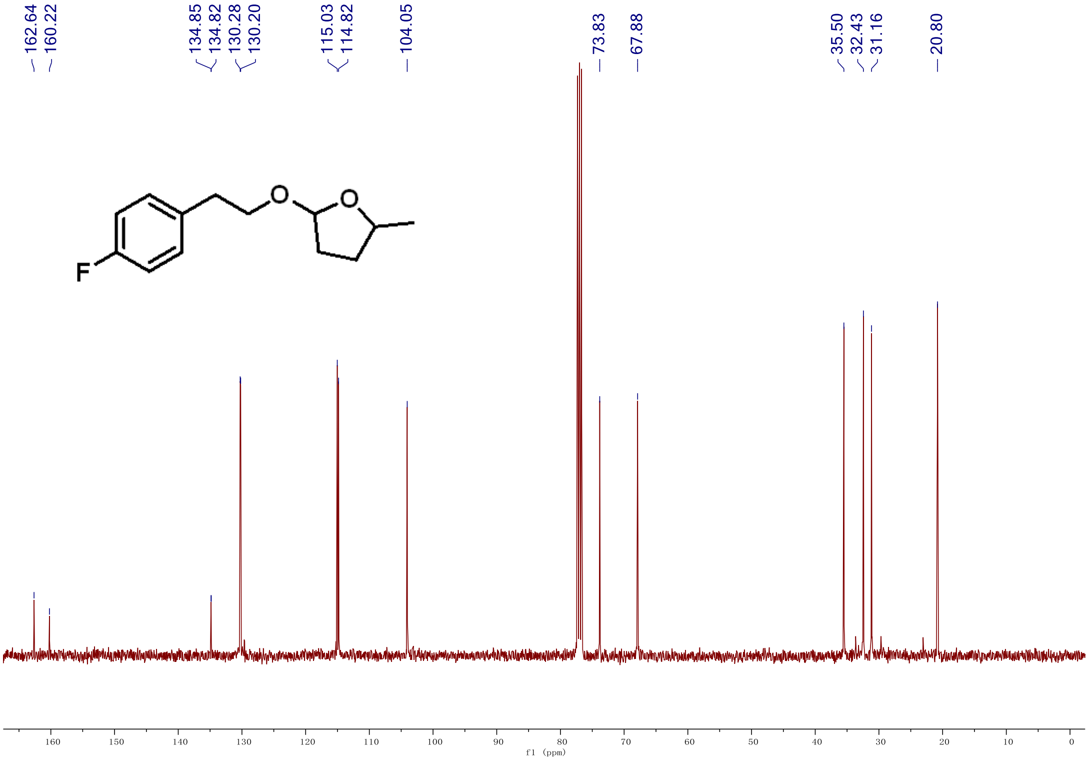
162.64
160.22
134.85
134.82
130.28
130.20
115.03
114.82
104.05
73.83
67.88
35.50
32.43
31.16
20.80
F
O
O
f1 (ppm)

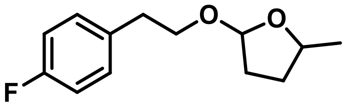
F
O
O

3ad’

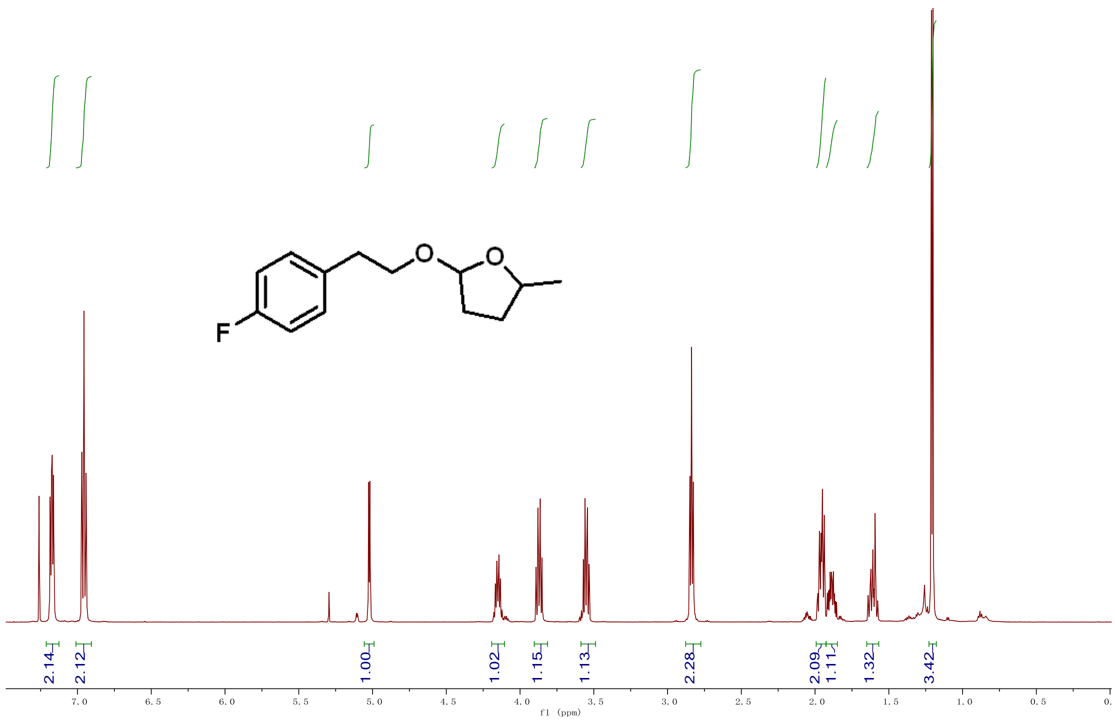
F
O
O
2.14
2.12
1.00
1.02
1.15
1.13
2.28
2.09
1.11
1.32
3.42
f1 (ppm)

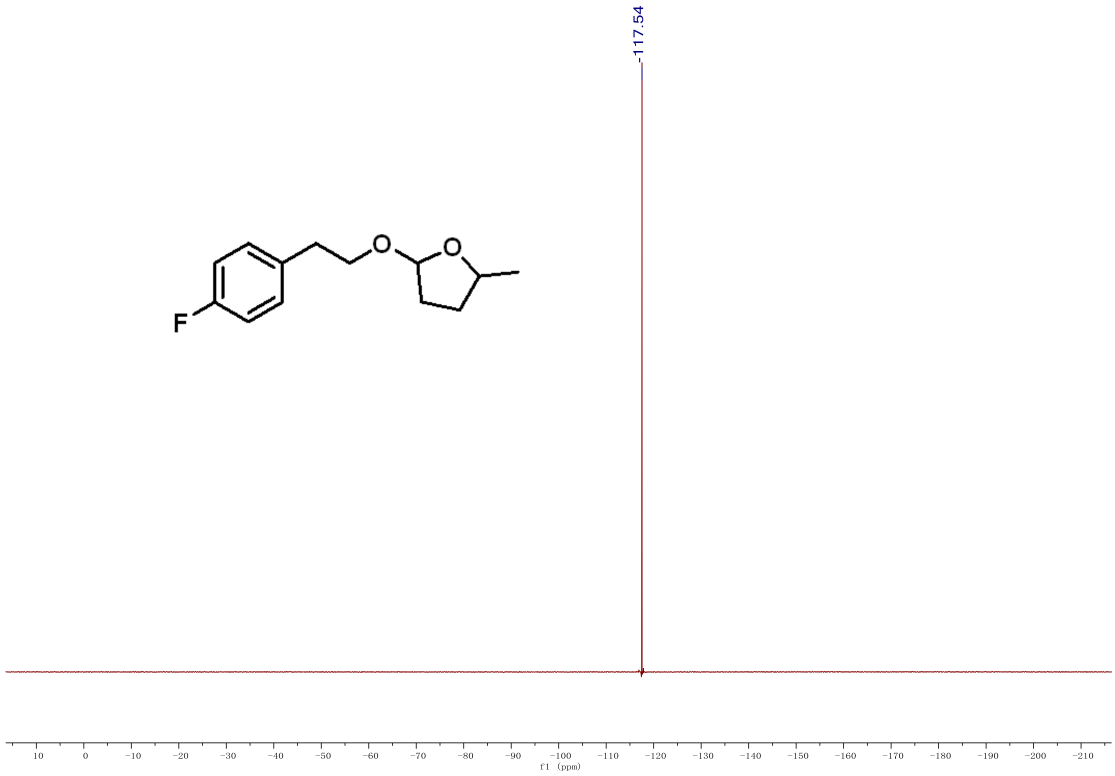
-117.54
F
O
O
f1 (ppm)

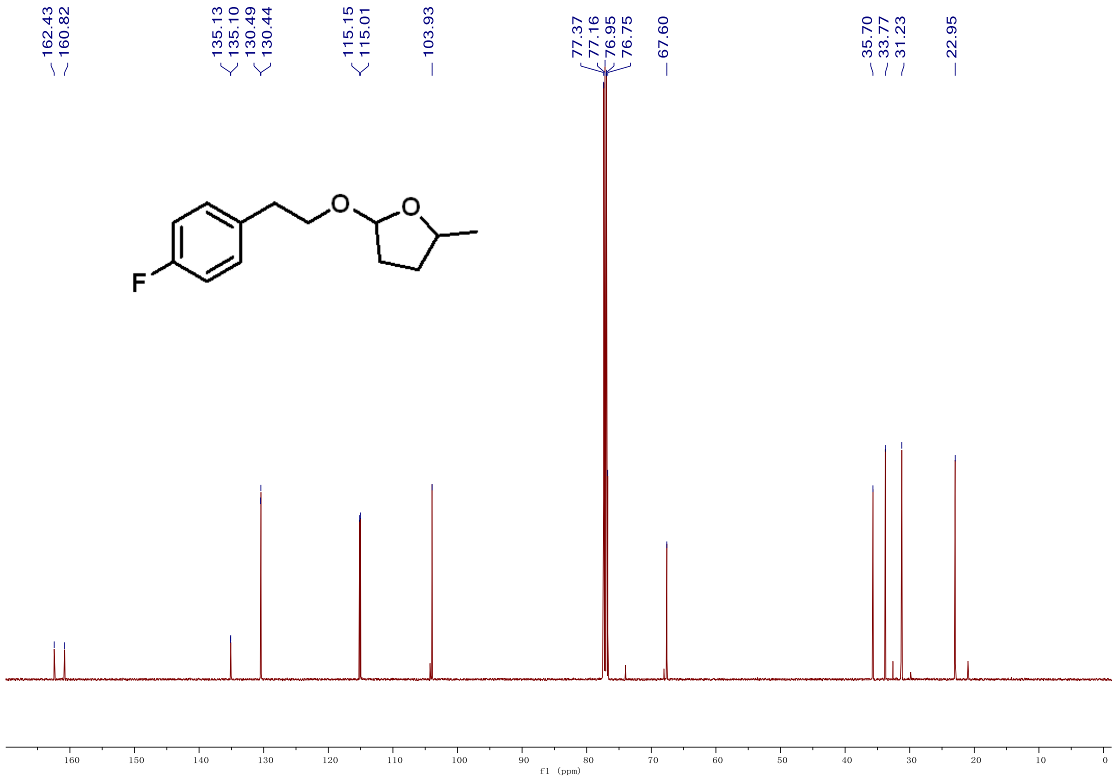
162.43
160.82
135.13
135.10
130.49
130.44
115.15
115.01
103.93
77.37
77.16
76.95
76.75
67.60
35.70
33.77
31.23
22.95
F
O
O
160
150
140
130
120
110
100
90
80
70
60
50
40
30
20
10
0
f1 (ppm)
